# Resilience of floral scent emission after florivory

**DOI:** 10.1111/plb.70209

**Published:** 2026-03-25

**Authors:** P. Tunes, Y. Canaveze, S. R. Machado, S. Dötterl, E. Guimarães

**Affiliations:** ^1^ Graduate Program in Plant Biology, Institute of Biosciences São Paulo State University Botucatu Brazil; ^2^ Laboratory of Ecology and Evolution of Plant–Animal Interactions, Department of Biodiversity and Biostatistics, Institute of Biosciences São Paulo State University Botucatu Brazil; ^3^ Department of Botany, Centro de Ciências Biológicas e da Saúde Universidade Federal de São Carlos (UFSCar) São Carlos Brazil; ^4^ Electron Microscopy Center of the Institute of Biosciences of Botucatu São Paulo State University Botucatu Brazil; ^5^ Department of Environment & Biodiversity, Plant Ecology University of Salzburg Salzburg Austria

**Keywords:** Chemical signalling, florivory, florivory‐induced chemical response, osmophores, pollination, volatile organic compounds

## Abstract

- Throughout evolutionary time, florivory has always represented an important source of selective pressure on flower evolution. When feeding, florivores remove corolla portions and undoubtedly change floral visual traits; however, we are in the early stages of understanding the effects of florivory on floral olfactory signalling. Florivores may jeopardize floral scent emission by feeding on flower parts responsible for volatile synthesis/emission, or by inducing plant physiological responses that alter floral scent. Thus, after florivory, flowers may no longer attract pollinators or might even repel them due to local short‐term changes in floral scent emission. Here, we investigate whether damage by florivores alters the amount and composition of floral scent.
- We describe natural florivory patterns and anatomically characterize the corolla portions potentially involved in scent emission in seven plant species pollinated by bees, hummingbirds, hawkmoths, or butterflies, which have diverse florivores, in a neotropical savanna. Moreover, we performed natural experiments to test whether florivory leads to short‐term local changes in floral scent phenotype.
- We found that even when florivores consumed 15% of the corolla portions with anatomical scent‐emitting features, there was no reduction in total amount of floral scent, except in a hawkmoth‐pollinated species, which showed a reduction in scent emission although only 3% of the osmophore‐bearing areas were consumed. Florivory did not affect scent composition in any species.
- Our data suggest a stability in post‐florivory scent emission, which may guarantee the maintenance of pollinator visitation regardless of florivory, indicating a resilience of natural systems with multiple and simultaneous interactions.

## Graphical abstract

Florivory is thought to affect floral traits, impacting pollination. However, our data suggest a stability in post‐florivory scent emission, which may guarantee the maintenance of pollinator visitation regardless of florivory, indicating a resilience of natural systems with multiple and simultaneous interactions. Created in BioRender. Tunes, P. (2026) https://BioRender.com/4n6apzg.

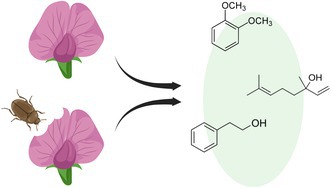

## INTRODUCTION

Florivory has been reported to occur since the Early Cretaceous (~103 Ma), evidenced by fossils from 30 million years after the earliest emergence of angiosperms (Xiao *et al*. 2021). Thus, this plant–animal interaction is older than the latest radiation of pollinators (Peris & Condamine 2024) and was already an established interaction during the Angiosperm Terrestrial Revolution, which occurred from c. 100 to 50 million years ago (Benton *et al*. 2022). Additionally, many (pollination) mutualisms might have evolved from antagonisms (*e.g*., Co‐Opted Antagonist Hypothesis, Johnson *et al*. 2021; Etl *et al*. 2022). Although both pollinators and florivores are attracted to flowers (Sasidharan *et al*. 2023), their ecological roles are typically conflicting (Kessler *et al*. 2013; Schiestl 2015). Florivores can reduce plant reproductive success by feeding on the reproductive organs, but also by altering floral cues (McCall & Irwin 2006; Zangerl & Berenbaum 2009) and interfering with the communication between the plant and the pollinator(s) (Chittka & Thomson 2001; Schaefer & Ruxton 2011). Florivory inherently affects floral visual traits (Mothershead & Marquis 2000; Malo *et al*. 2001), if florivores remove, for example, portions of the perianth; however, how florivory affects floral scent traits, which act in olfactory signalling to pollinators (Raguso 2008; Dötterl & Gershenzon 2023), is still poorly understood. However, the few studies available show that florivory can trigger physiological responses in flowers, as is well known for herbivory in leaves (Kessler & Baldwin 2002; Erb *et al*. 2008; Dicke & Baldwin 2010; Rusman *et al*. 2019), resulting in qualitative and quantitative changes in floral scent emissions (Farré‐Armengol *et al*. 2015). Since florivory is a type of herbivory, one could expect that floral tissue removal would also trigger changes in floral volatiles including changes in total scent emission and scent blend.

Additionally, florivores may not only consume tissues that are not involved in the biosynthesis and emission of floral scents, but also specific floral areas bearing tissues with scent‐emitting features (*sensu* Effmert *et al*. 2006; Knudsen & Gershenzon 2020). In this case, florivores may reduce the area of biosynthesis and the physical surface of the flower that is responsible for scent emission and, consequently, one could expect that they would reduce the amount/blend of scent emitted by flowers. Overall plant–pollinator communication pathways can be affected by florivory due to various means, and consequently, pollinators may avoid flowers damaged by florivores due to changes of the floral scent phenotype (Zangerl & Berenbaum 2009; Kessler *et al*. 2011; Schiestl *et al*. 2014; Vega‐Polanco *et al*. 2020).

Therefore, this study aims to contribute to an integrated understanding of the interference of florivory on floral scent emission, focusing on scenarios in which florivores do not neuter flowers. Specifically, we selected seven plant species with different pollination systems and florivores to (i) identify the areas of the corolla that show features associated with scent emission and investigate whether florivores feed on these corolla portions; and (ii) investigate whether florivory leads to changes in the total amount and relative composition of floral scent. We test the hypothesis that florivores directly reduce the amount of scent emission due to the removal of corolla areas with scent‐emitting features.

## MATERIAL AND METHODS

To achieve our goals, we selected seven plant species from savanna physiognomy of Cerrado that are pollinated by bees, butterflies, hawkmoths, or hummingbirds (Table 1), and whose flowers are consumed by beetles, caterpillars, and grasshoppers. Six of the species are pollinated by strongly scent‐oriented animals (bees, butterflies, and hawkmoths) and one species is pollinated by animals that do typically not rely strongly on scent (hummingbirds). *Amphilophium mansoanum* (Bignoniaceae), *Lantana camara* (Verbenaceae), *Tocoyena formosa* (Rubiaceae), and *Zeyheria montana* (Bignoniaceae) are allogamous and rely completely on pollinators for fruit production (Silberbauer‐Gottsberger 1972; Barrows 1976; Bittencourt & Semir 2004, *unpublished data* for *A. mansoanum*), whereas *Byrsonima intermedia* (Malpighiaceae) and *Lippia alba* (Verbenaceae) are predominantly allogamous and insect‐pollinated (Schocken 2007; Boas *et al*. 2013; Balestra *et al*. 2014), and *Centrosema pubescens* (Fabaceae) has a mixed mating system (Penteado *et al*. 1996; Sousa *et al*. 2011). Field experiments were performed from March to November of 2018, and anatomical and histochemical studies were conducted in 2022. All plant experiments were conducted in accordance with relevant institutional, national, and international guidelines and legislation. The authorization for collection of biological samples is registered on Sisgen under #A90A83C and #A8C28D6.

**Table 1:** Plant species and their pollinators.

plant species (Family)	pollinators	references	sampling sites
*Amphilophium mansoanum* (DC.) L.G.Lohmann (Bignoniaceae)	Large bees	Balduino *et al*. (2023)	22°49′S 49°13′W
*Byrsonima intermedia* A. Juss. (Malpighiaceae)	Oil‐collecting bees	Boas *et al*. (2013)	22°48′S 49°11′W
*Centrosema pubescens* Benth. (Fabaceae)	Large bees	Borges (2006)	22°49′S 49°13′W
*Lantana camara* L. (Verbenaceae)	Butterflies	Schemske (1976)	22°47′S 49°14′W
*Lippia alba* (Mill.) N.E.Br. ex P. Wilson (Verbenaceae)	Butterflies	Venâncio *et al*. (2016)	22°53′S 48°27′W
*Tocoyena formosa* (Cham. & Schltdl.) K.Schum. (Rubiaceae)	Hawkmoths	Silberbauer‐Gottsberger & Gottsberger (1975)	22°47′S 49°14′W
*Zeyheria montana* Mart. (Bignoniaceae)	Hummingbirds	Bittencourt & Semir (2004)	22°54′S 48°30′W

### Characterization of corolla portions potentially involved in scent emission

We characterized corolla portions potentially involved in scent emission by using a combination of three complementary approaches. First, we used Vogel's neutral red test (Dafni *et al*. 2005) to obtain an overview of the potential scent‐emitting corolla portions. Second, we employed histological analyses to complement Vogel's test, as they allow us to identify whether the stained areas do indeed correspond to tissues with scent‐emitting features, such as osmophores that have a specific cellular organization (Effmert *et al*. 2006). Third, we used histochemistry to confirm the involvement of these portions of the flowers in scent biosynthesis/emission, considering that the accumulation of starch grains, lipophilic compounds, and terpenes are associated with the secretion of floral scent (Stern *et al*. 1986; Ascensão *et al*. 1999; Effmert *et al*. 2006). In addition to these three analyses, we performed surface analysis to obtain an overview of the overall surface appearance (indumentum, texture, and microstructure) of the corolla portions involved in scent emission. Combining all these methods and approaches increases the confidence with which we can determine whether (or not) the damage caused by florivores is affecting portions of the corolla that are involved in scent production and emission.

For the initial test with Vogel's neutral red, we sampled three to five recently opened flowers/inflorescences per species (Table 1) and maintained every unit in an aqueous solution of neutral red (0.01%, pH 8) for 30–45 min. Thereafter, we washed every unit in water, analysed it carefully, described the location of the stained area and photographed the material. The results of the Vogel's neutral red test showed which areas of the corolla would be used for the anatomical analysis and histolocalization of the main classes of compounds associated with scent emission. Thus, for anatomical analysis, we sampled the central upper portion of the corolla tube for *Amphilophium mansoanum*, the medium portion of the upper petal for *Byrsoima intermedia*, the central portion of the standard petal for *Centrosema pubescens*, the corolla tube entrance for *Lantana camara*, petal lobes for *Lippia alba*, the central portion of a petal lobe for *Tocoyena formosa*, and the corolla tube entrance for *Zeyheria montana*. The samples obtained from recently opened flowers were fixed in Karnovsky's reagent (Karnovsky 1965) for 48 h, dehydrated in an alcoholic series and embedded in glycol‐methacrylate resin (Historesin, Leica Instruments®). Transverse and longitudinal sections, approximately 5 μm thick, were obtained using a semi‐automated rotating microtome (Leica Biosystems RM2245, Wetzlar, Germany). Sections were stained with 0.05% toluidine blue, pH 4.7, for histological characterization (O'Brien *et al*. 1964). For histolocalization of groups of substances commonly associated with floral scent‐emitting tissues, we used Lugol reagent to detect starch grains (Johansen 1940), Sudan IV to detect lipidic substances (Johansen 1940) and NADI reagent to detect terpenoids (David & Carde 1964). We ran appropriate controls simultaneously for each test. We analysed and documented slides for anatomical characterization and histochemical tests under a light microscope (Olympus BX 41) equipped with a digital camera (Olympus C 5050).

For surface analysis, samples measuring approximately 1 cm^2^, obtained from recently opened flowers, were immediately fixed in Karnovsky's reagent (Karnovsky 1965) for 24 h, dehydrated in an acetonic series, and dried in critical point equipment (Bal‐Tec CPD 030). The samples were glued to aluminium sample holders and vacuum metallized (Bal‐Tec SCD 050) with about 10 nm of gold. The analyses were performed using a scanning electron microscope (SEM) Quanta 200 ‐ FEI (FEI, Hillsboro, OR, USA) at 20 kv.

### Florivores and florivory patterns

We performed approximately 80 h (10–15 h per species) of field observations during the day and night (0600 to 2400 h) to characterize the most frequent florivore feeding on each plant species. The observations were performed by two people, on five individuals per plant species, during 20 min per hour. The morphotypes of florivores were identified in the field at order level. We registered the florivores feeding on flowers, described the patterns of florivory, and visually estimated the flower areas removed. Additionally, we later cross‐referenced the areas of the corolla stained by Neutral red and the areas consumed by florivores to estimate how much of the scent‐emitting tissue was removed by florivores.

### Effects of florivory on floral scent

**Fig. 1:**
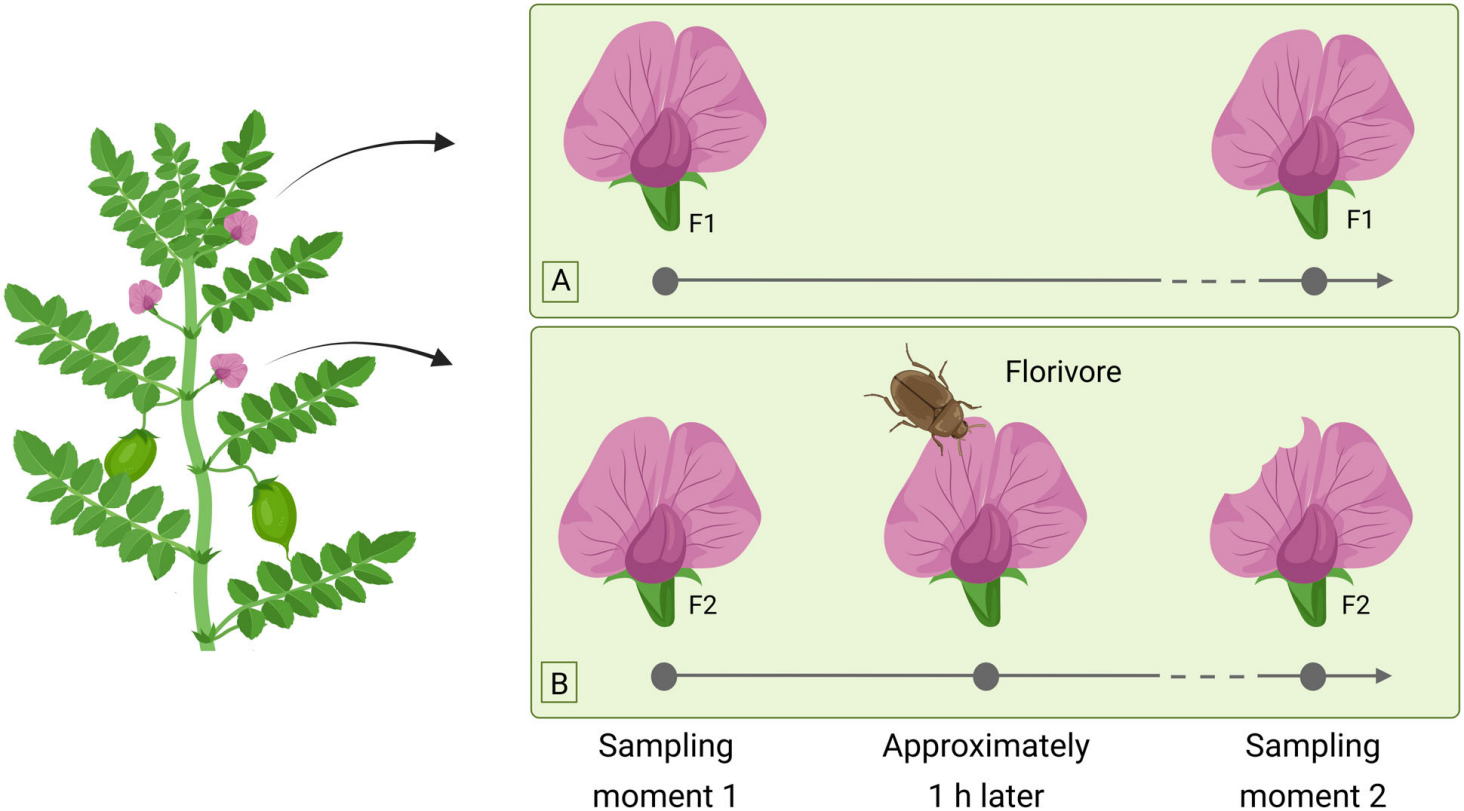
Schematic representation of the experimental design used to determine if florivory alters the emission of floral volatiles. (A) Flowers that remained intact during the whole experiment without ever being exposed to florivores. (B) Flowers that were initially intact and were later subjected to damage by florivores. Floral scent was collected in situ from flowers/inflorescences of study plant individuals at Sampling moment 1, when both flowers (F1 and F2) were intact, and at Sampling moment 2, when one flower was kept intact (F1) and the other was fed on by florivores (F2). Florivores were inserted on F2 just after the first scent sampling and the time elapsed between florivore insertion and the Sampling moment 2 was variable and is shown in Table S4. Created in BioRender. Tunes *et al*. (2022) https://BioRender.com/nt30dbr.

We characterized floral scent before and after damage by florivores of the same flower. As scent is known to vary among flowers within an individual (*e.g*., Ayasse *et al*. 2000) and throughout the flower lifetime (Delle‐Vedove *et al*. 2017), we characterized the scent of other non‐damaged flowers of the same age in the same plant in parallel. This experimental design allowed us to isolate the effect of florivory from the effect of the passage of time. Initially (Sampling moment 1 in Fig. 1), we collected volatiles from two recently opened intact flowers/inflorescences per plant, *in situ*, at the time of pollinator activity (see Table S1). Immediately thereafter, we placed 1–3 individuals of the main florivore of each plant species on one of the two flowers/inflorescences approximately 1 h after the first sampling of floral scent, and bagged the flower with the florivore (Fig. 1). The florivores had been previously collected in the field and kept without food for approximately 3 h prior to being added to the flowers. We kept the florivores on the flowers during enough time to allow them to cause the natural feeding pattern observed in nature (previously determined through field observations). Florivore activity coincided with pollinator activity but was not restricted to it. Some florivores are naturally more voracious and feed on a flower as soon as they arrive, while others demand longer periods of time to feed. Thus, our criterion was to ensure that the natural feeding pattern was performed in each plant species, regardless of how long the florivore needed to do so. After the florivores fed on the flower/inflorescence, we again collected volatiles from both flowers/inflorescences (Sampling moment 2 in Fig. 1). The period during which florivores fed on flowers/inflorescences was variable, reflecting their period of activity observed in the field, and is shown in Table S1, and Sampling moment 2 started immediately after the florivores were removed from the flowers. In the Sampling moment 1, we sampled two flowers each from seven plants of *A. mansoanum*, five plants of *B. intermedia*, five of *C*. *pubescens*, two of *L. camara*, three of *L. alba*, seven of *T. formosa*, and 10 of *Z. montana*. Performing the second sampling of the flowers sometimes led to the flowers breaking from the plants. Additionally, sometimes florivores did not feed on the flowers. In cases where this happened, we discarded the second scent samples from these plants. We used the first samples to increase our number of samples of the control. Therefore, in the Sampling moment 2, we had fewer plants, and for some species, the number of Control and Damaged flowers sampled was not the same (see Table S2).

We collected volatile samples following the protocol by Dötterl *et al*. (2005). The flowers (or inflorescences, see Table S2) were wrapped with polyethylene bags (the size of the bags used varied from 7 × 7 cm to 8 × 15 cm according to flower/inflorescence size, but was the same for control and treatment flowers/inflorescences within species), and the volatiles that accumulated inside the bag were collected on adsorbent tubes with the aid of a vacuum pump (flow of 200 ml/min). The duration of collections varied from 15 to 60 min (Table S2) depending on the strength of the scent as perceived to the human nose.

The adsorbent tubes, 20 mm in length and 2 mm in internal diameter, were filled with a mixture of 1.5 mg Tenax‐TA (60–80 mesh) and 1.5 mg Carbotrap B (20–40 mesh; both Supelco, Germany), contained by glass wool. Volatiles collected from leaves served as negative controls. The samples were stored in a freezer at −20 °C until the moment of analysis in an automated thermodesorption system (TD 20, Shimadzu, Tokyo, Japan) attached to a gas chromatograph coupled to a mass spectrometer (GC/MS‐QP2010 Ultra, Shimadzu, Tokyo, Japan). This setup was equipped with a ZB‐5 fused silica column (60 m long, 0.25 mm of inner diameter, 0.25 μm of film thickness), and a constant 1.5 mL/min flow of helium as the carrier gas was maintained. The injector temperature was 200 °C and the samples were injected in variable split modes (split 1, 5, 10, 25, and 50), based on preliminarily analyses of test samples of each species. The oven temperature started at 40 °C, then increased by 6 °C/min to 250 °C, when it was kept constant for 1 min. The MS interface was set at 250 °C. Mass spectra were taken at 70 eV (in EI mode), with a scanning range of 30–350 m/z. The data were analysed using the GCMSolution package, Version 4.41 (Shimadzu). We identified volatile compounds by comparing their linear retention indices *sensu* van den Dool & Kratz (1963), based on a series of n‐alkanes, and mass spectra to data available in the databases ADAMS (Adams 2007), ESSENTIALOILS‐23P, FFNSC 2, Wiley 9 and Nist11.

We confirmed the identities of some compounds by comparing their retention indices and mass spectra to those of authentic reference standards available in the collection of the Plant Ecology lab of the University of Salzburg. For quantitative analysis of VOCs, we injected 100 ng of each of about 150 compounds, including monoterpenes, aliphatic, and aromatic compounds, into the GC–MS system. We used the mean peak area (total ion current) of these compounds to estimate the total amount of floral odour available in each sample, according to Etl *et al*. (2016). Compounds that did not occur in the control samples or occurred in at least five times higher amounts in floral than control samples were considered as floral compounds.

We used PERMANOVAs (9999 permutations) based on Euclidean distances and Bray–Curtis dissimilarities to assess whether there are differences in the total amount of scent and the relative amounts of compounds, respectively, between intact and damaged flowers/inflorescences. We also used PERMANOVAs (9999 permutations) based on Euclidean distances to assess whether there are differences in the relative amounts of each individual scent compound between intact and damaged flowers/inflorescences. For these analyses, we used a factorial sampling design, considering the treatment applied to the flower (intact or damaged by florivores) and the Sampling moment (1 or 2) as fixed factors, and plant individuals as a random factor. A significant Treatment*Sampling moment interaction would indicate a local short‐term effect of florivory on floral scent emissions. Data obtained from *L*. *camara* and *L*. *alba* were not analysed by PERMANOVA because the number of possible unique permutations was too low (18 and 214, respectively) for obtaining robust outcomes, due to low sample sizes.

We used non‐metric multidimensional scaling (NMDS), based on the Bray–Curtis dissimilarities, to visualize similarities and dissimilarities in relative amounts of compounds among the samples within the different species. All PERMANOVA and NMDS analyses were performed with Primer 6 v. 6.1.15 with PERMANOVA+ v. 1.0.5 (Clarke & Gorley 2006). To create violin plots and graphically compare the total amount of volatile compounds emitted by intact and damaged flowers, we used R v. 4.0.2 (R Core Team 2020) with the package ggplot2 (Wickham 2016).

## RESULTS

### Characterization of corolla tissues potentially involved in scent emission

**Fig. 2:**
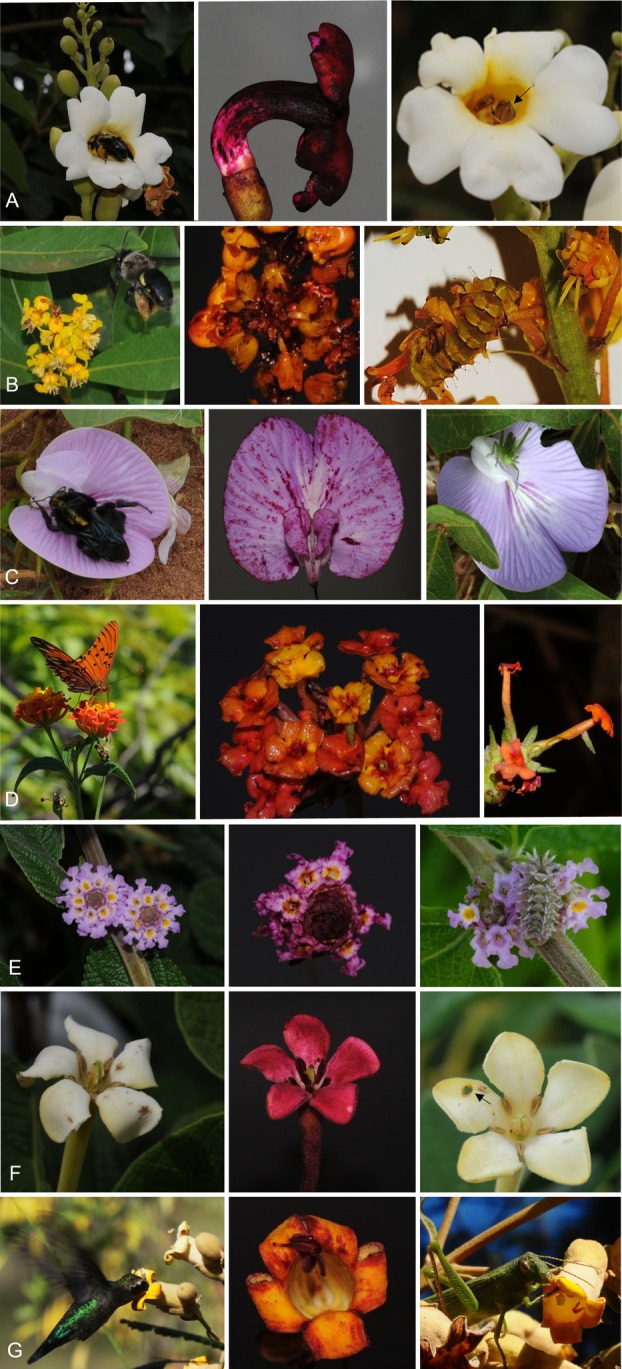
Flowers, pollinators, flower portions stained by neutral red solution, florivory patterns and florivores interacting with the study plant species. Row A: *Amphilophium mansoanum*. Row B: *Byrsonima intermedia*. Row C: *Centrosema pubescens*. Row D: *Lantana camara*. Row E: *Lippia alba*. Row F: *Tocoyena formosa*. Row G: *Zeyheria montana*. [Column on the left] Intact flowers in field conditions with their respective pollinators (in rows A–D, G). [Central column] Flowers stained with Vogel's neutral red solution. [Column on the right] Flowers that were fed on by florivores, showcasing the most frequent florivory pattern registered in the field and florivores (in rows A–C, E, G).

**Fig. 3:**
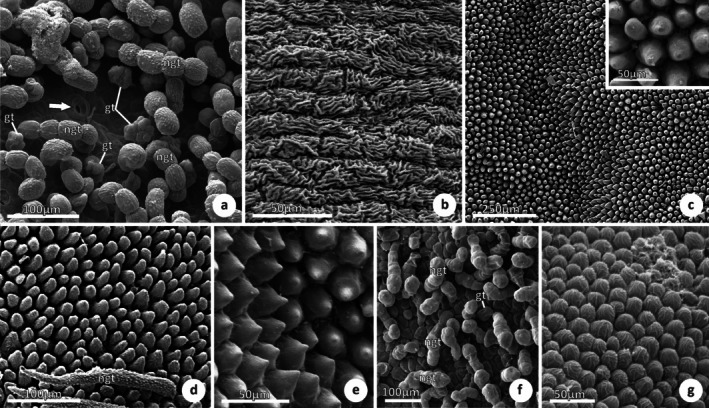
Surface characterization of floral scent‐emitting tissues of the study plant species; Scanning electron micrographs. (A) *Amphilophium mansoanum*; central upper portion of the corolla tube. (B) *Byrsonima intermedia*; medium portion of the upper petal. (C) *Centrosema pubescens*; central portion of the standard petal. (D) *Lantana camara*; corolla tube entrance. (E) *Lippia alba*; petal lobes. (F) *Tocoyena formosa*; central portion of a petal lobes. (G) *Zeyheria montana*; petal lobes. (A) Adaxial flattened and smooth surface showing glandular and non‐glandular trichomes and stomata (arrow). (B) Adaxial flattened surface with deeply striated cuticle. (C) Adaxial papillose surface with smooth cuticle. Detail showing the conical cells of the epidermis. (D) Adaxial papillose surface comprised by conical cells of the epidermis and non‐glandular trichomes. (E) Adaxial papillose surface comprised by conical cells of the epidermis. (F) Adaxial flattened surface with smooth cuticle and numerous prostrate glandular and non‐glaVndular trichomes. (G) Adaxial papillose surface comprised by globose epidermal cells with striate cuticle. Symbols: gt: glandular trichome; ngt: non‐glandular trichome.

**Fig. 4:**
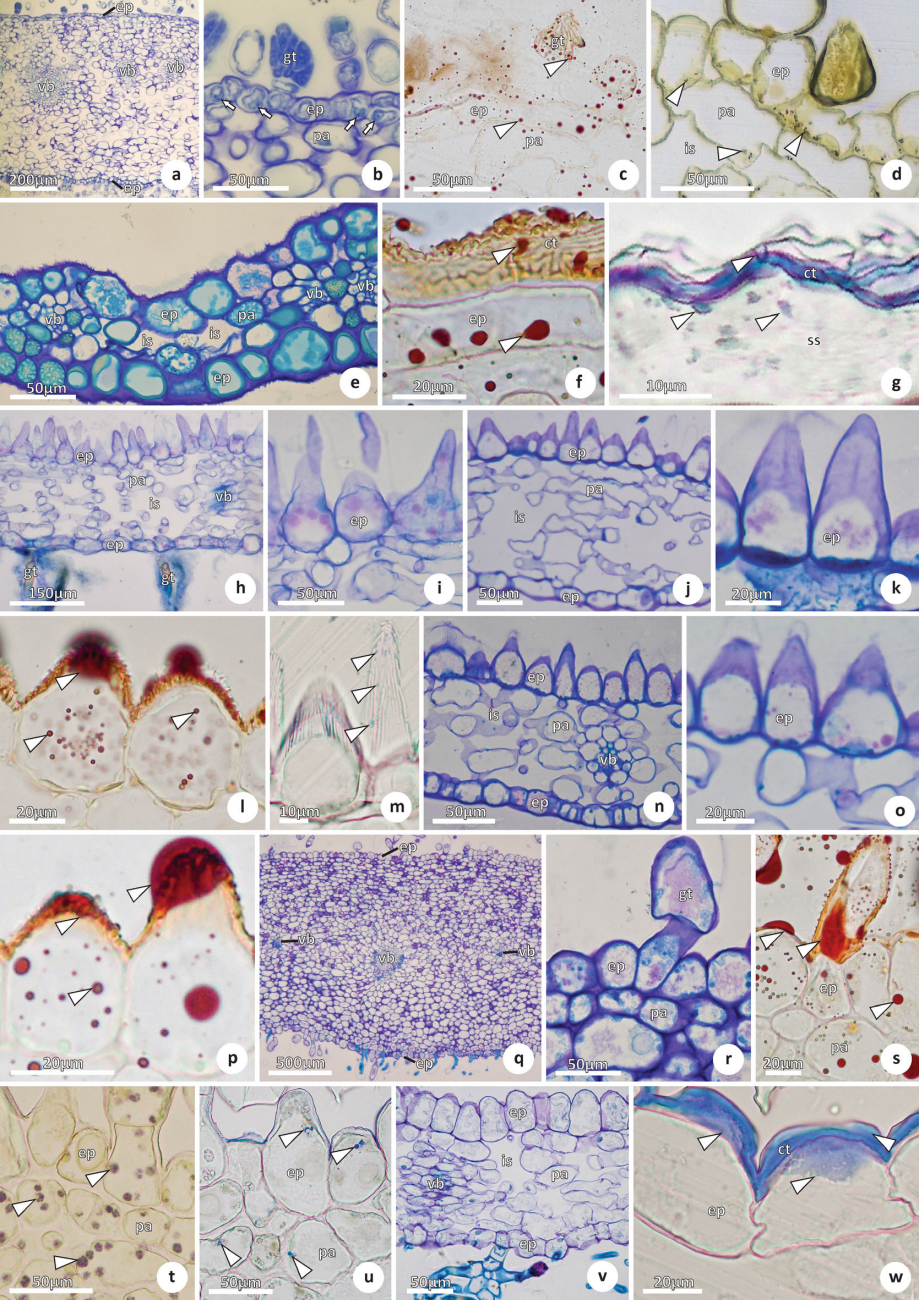
Anatomical and histochemical characterization of scent‐emitting tissues of the corolla of flowers from the study plant species. Transversal sections; A–B, E, H–K, N–O, Q–R, V, toluidine blue. (A–D). *Amphilophium mansoanum*; central upper portion of the corolla tube. (E–G) *Byrsonima intermedia*; medium portion of the upper petal. (H–I) *Centrosema pubescens*; central portion of the standard petal. J‐m. *Lantana camara*; corolla tube entrance. (N–P). *Lippia alba*; petal lobes. (Q–U) *Tocoyena formosa*; central portion of a petal lobes. (V–W). *Zeyheria montana*; petal lobes. (A) General view showing uniseriate epidermis in both surfaces, mesophyll composed by several parenchyma layers and vascular bundles. (B) Adaxial side exhibiting trichomes and a uniseriate secretory epidermis composed by flattened cells, with dense content and large nuclei (arrows), and subepidermal secretory parenchyma layers. (C) Lipid droplets (arrowheads) in the epidermal and subepidermal cells and glandular trichome. (D) Starch grains (arrowheads) in the epidermal and subepidermal cells. (E) General view showing uniseriate epidermis, loosely arranged parenchyma and vascular bundles. Note dense content in the adaxial epidermal cells. (F) Lipid droplets (arrowheads) on and immersed in the cuticle, and in the epidermal cells. (G) Terpene bodies (arrowheads) on the cuticle and subcuticular space. (H) General view showing adaxial secretory epidermis composed of tall conical cells, loosely arranged parenchyma with large intercellular spaces, vascular bundle, and flattened abaxial epidermis with glandular trichomes. (I) Conical epidermal cells with large vacuoles and dense inclusions. (j) General view showing adaxial secretory epidermis composed of conical cells, loosely arranged parenchyma with large intercellular spaces, and flattened abaxial epidermis. (K). Conical epidermal cells with large vacuoles and flocculate inclusions. (L) Lipid droplets (arrowheads) in the secretory epidermal cells. (M) Terpene bodies (arrowhead) in the epidermal cells. (N) General view showing adaxial secretory epidermis composed of conical cells, loosely arranged parenchyma with large intercellular spaces, vascular bundle, and flattened abaxial epidermis. (O) Conical epidermal cells with large vacuoles and dense inclusions. (P) Lipid droplets (arrowheads) in the secretory epidermal cells. (Q) General view showing uniseriate epidermis, tightly arranged parenchyma without intercellular spaces, vascular bundle, and trichomes in both surfaces. (R) Adaxial secretory epidermis and subepidermal layers composed of tightly arranged flattened cells with dense content and large vacuoles. (S) Lipid droplets (arrowheads) in the trichome, secretory epidermis and subepidermal layers. (T) Starch grains (arrowheads) in the trichome, epidermal and subepidermal cells. (U) Terpene bodies (arrowhead) in the epidermal and subepidermal cells. (V) General view showing adaxial uniseriate epidermis composed by globose secretory cells, loosely arranged parenchyma with large intercellular spaces, vascular bundle, and flattened abaxial epidermis with branched trichome. (W) Terpene bodies (arrowhead) in the protoplast of epidermal cells and on the cuticle. Symbols: ct: cuticle; ep: epidermis; gt: glandular trichome; is: intercellular space; pa: parenchyma; vb: vascular bundle.

In all species, neutral red solution stained portions of the corolla, variable among species in location, size and shape, indicating the presence of potential scent‐emitting areas (Table 2; Fig. 2A–G). The micromorphological (Fig. 3) and anatomical analyses (Fig. 4) showed that the regions stained with neutral red (Fig. 2A–G) had typical features of scent‐emitting tissues.

**Table 2:** Morphological and anatomical characteristics of floral scent‐emitting areas for the seven studied species.

plant species	cuticle	epidermis surface	scent‐emitting tissues	glandular trichomes
*Amphilophium mansoanum* (DC.) L.G.Lohmann (Bignoniaceae)	Smooth	Slightly papillose	Uniseriate epidermis	Present
*Byrsonima intermedia* A. Juss. (Malpighiaceae)	Deeply striate	Flattened	Uniseriate epidermis	Absent
*Centrosema pubescens* Benth. (Fabaceae)	Smooth	Papillose	Uniseriate epidermis	Present
*Lantana camara* L. (Verbenaceae)	Striate	Papillose	Uniseriate epidermis	Present
*Lippia alba* (Mill.) N.E.Br. ex P. Wilson (Verbenaceae)	Striate	Papillose	Uniseriate epidermis	Present
*Tocoyena formosa* (Cham. & Schltdl.) K.Schum. (Rubiaceae)	Smooth	Flattened	Epidermis and subepidermal layers	Present
*Zeyheria montana* Mart. (Bignoniaceae)	Striate	Papillose	Uniseriate epidermis	Present

In the tissues with scent‐emitting features, the cuticle ranged from smooth to wrinkled and to deeply striated (Table 2; Fig. 3A–G). Additionally, the epidermis surface varied among flattened in *B*. *intermedia* and *T*. *formosa* (Fig. 3B,F), slightly papillose in *A*. *mansoanum* (Fig. 3A), and papillose in the other four species (Table 2), with epidermal cell shape varying from conical in *C*. *pubescens*, *La*. *camara*, and *Li*. *alba* (Fig. 3C–E) to globose in *Z*. *montana* (Fig. 3G). Additionally, we verified the presence of stomata (Fig. 3A) and glandular trichomes in tissues with scent‐emitting features in most species (Fig. 3A,F; Fig. 4B,H,R–T, Table 2), except for *B*. *intermedia* (Fig. 3B).

Typical osmophores, composed of epidermis and subjacent parenchyma cell layers, were observed in *A*. *mansoanum* (Fig. 4A–D) and *T*. *formosa* (Fig. 4Q–U). In *B*. *intermedia*, *C*. *pubescens*, *L*. *camara*, and *L*. *alba*, and *Z*. *montana*, only cells of the epidermis showed scent‐emitting features (Table 2, Fig. 4E–P,V–W). In the adaxial surface of all species, the epidermal cells were voluminous, had large nuclei (Fig. 4B), and had dense content (Fig. 4I,K,O,V). The five species that did not show typical osmophores had loosely arranged parenchyma with smaller cells and large intercellular spaces (Fig. 4E,H,J,N,V). *Amphilophium mansoanum* and *T*. *formosa* showed tightly arranged subepidermal layers, with large cells with dense content (Fig. 4A,B,Q,R) and large nuclei (Fig. 4B).

Histochemical analyses showed positive reactions for starch grains, total lipids, and terpenoids. With the exception of *B. intermedia*, all species showed glandular trichomes located in the neutral red stained regions with a positive reaction to NADI reagent suggesting that they participate in terpenoid synthesis and emission. Terpenoids (Fig. 4G,M,U,W) were detected in the epidermal cells of all species and in glandular trichomes and subepidermal cells in *T*. *formosa* (Fig. 4U). We found lipid droplets (Fig. 4C,F,L,P,S) in the epidermal cells of most plant species, except for *Z*. *montana*. We also found lipid droplets in the trichomes of *A*. *mansoanum* (Fig. 4C), *C*. *pubescens* (Fig. 4R), and *T*. *formosa* (Fig. 4S). Lastly, we detected starch grains in the epidermal and subepidermal cells in *A*. *mansoanum* (Fig. 4D) and in *T*. *formosa* (Fig. 4T).

### Florivores and florivory patterns

We registered a variety of florivores in the field, including insects (beetles, butterfly caterpillars, grasshoppers), and birds (passerine birds) (Table 3). We verified that in six of the seven sampled species, the florivores fed on perianth portions with scent‐emitting features (Table 3, Fig. 2A–C,E–G). The only exception was *L. camara* that had tissues with scent‐emitting features placed at the entrance of the corolla tube, showing a ring‐like shape, but florivory was registered only on lobe margins and at the base of the corolla tube (Table 3, Fig. 2D). The damages caused by florivores in our experimental setting were similar to those observed in natural conditions.

**Table 3:** Plant species, flower portions stained by neutral red indicating the presence of floral scent‐emitting tissues, florivores and their feeding patterns with focus on flower portions commonly fed on by them, and visually estimated area of the flower that was consumed by florivores, and stained area (neutral red test) typically removed by the most common florivore.

plant species	flower portions stained by neutral red	florivores and feeding pattern	area of the flower/inflorescence removed (%)	stained area removed by florivores (%)
*Amphilophium mansoanum* (DC.) L.G. Lohmann (Bignoniaceae)	Corolla tube until close to the constriction at the bottom of the floral tube, corolla lobe margins	**Beetles** made holes on the dorsal and lateral portions of corolla tube, just above the tube curve	5–6	8–9
*Byrsonima intermedia* A. Juss. (Malpighiaceae)	Petals and styles	Beetles and **caterpillars** fed on petals and stamens	18–20	9–10
*Centrosema pubescens* Benth. (Fabaceae)	Entire margin and spots spread all over the petals	**Beetles** made small holes spread on banner and grasshoppers fed on the banner margin	4–7	8–14
*Lantana camara* L. (Verbenaceae)	Ring at the corolla tube entrance	**Beetles** fed on the margins of corolla lobes and the basis of the corolla tube	1–2	0
*Lippia alba* (Mill.) N.E.Br. ex P. Wilson (Verbenaceae)	Distal portions of the corolla lobes, spots on the floral tube entrance, bracts at the centre of the inflorescence	**Caterpillars** fed on the floral buds covered by bracts at the centre of inflorescence and inner flowers; beetles fed on the corolla lobes	20–30	10–15
*Tocoyena formosa* (Cham. & Schltdl.) K.Schum. (Rubiaceae)	Entire corolla, anthers, stigma margins	**Beetles** made small holes in the petal lobes	2–3	2–3
*Zeyheria montana* Mart. (Bignoniaceae)	Margins of corolla lobes, anthers, stigma	Beetles, **grasshoppers**, and birds removed part of the corolla lobes, and beetles additionally made holes in floral tube	16–30	12–15

The most frequent or the only florivore group observed for each plant species is in bold.

### Evaluating the effects of florivory on floral scent

Floral scent was detected in all the studied species (see Table S3 for detailed composition), with 4–52 compounds recorded per species. Some species presented weak scents, lower than 100 ng of scent/flower/h (see Table S3), such as *B. intermedia* (43–47 compounds, mainly terpenoids, but also with aliphatic and aromatic compounds, dominated by (*E*)‐β‐caryophyllene and α‐humulene), *L. alba* (41–44 compounds, mainly terpenoids, but also with aliphatic and aromatic compounds, dominated by linalool), and *Z. montana* (13–20, mainly aromatic compounds, but also terpenoids and aliphatic compounds, dominated by benzyl alcohol). Other species presented strong scents, higher than 400 ng of scent/flower/h, such as *A. mansoanum* (30–43 compounds, mainly terpenoids, but also aliphatic and aromatic compounds, dominated by (*E*)‐β‐ocimene) and *T. formosa* (27–29, mainly aromatic compounds and terpenoids, also N‐bearing compounds, dominated by methyl benzoate and (*E*)‐linalool oxide pyranoid). The amount of scent of *L. camara* (4–8 compounds, mainly terpenoids, but also aliphatic and aromatic compounds, dominated by linalool and methyl octanoate) and *C. pubescens* (37–52 compounds, mainly terpenoids, but also with aliphatic and aromatic compounds, dominated by 2‐phenylethanol) was in between the weak‐ and strong‐scented species (100–200 ng of scent/flower/h).

**Fig. 5:**
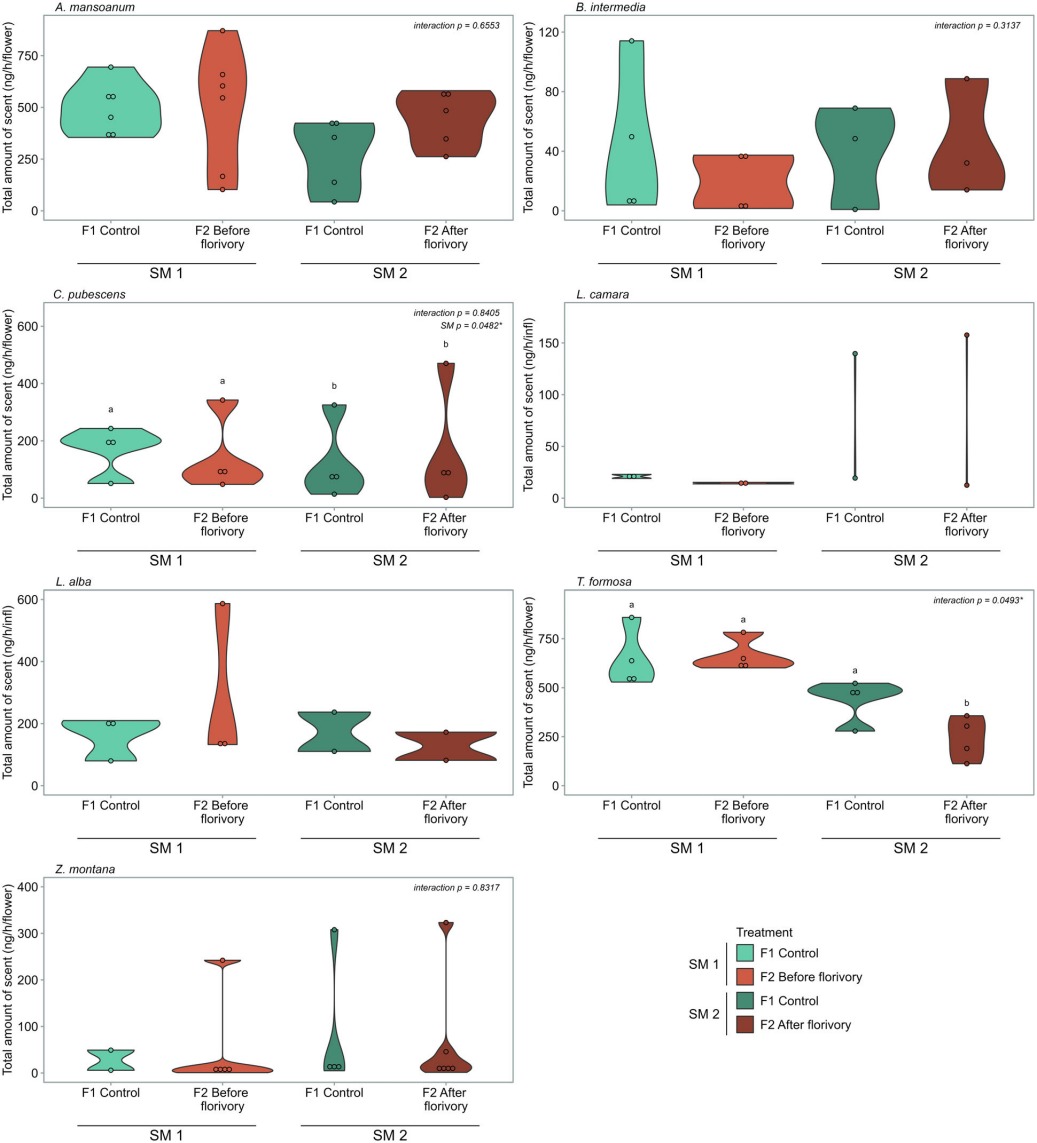
Violin plots of the total amount of scent emitted by florivore‐damaged and non‐damaged flowers/inflorescences (see Table S3) of the studied plant species (ng.flower or inflorescence^−1^.h^−1^) at the Sampling moment 1 and 2 (SM1 and SM2). The p‐values of the ‘Treatment*Sampling moment’ interaction effect obtained by PERMANOVA analyses are indicated, and when significative, ‘Sampling moment’ effect is indicated.

In *T. formosa*, but not in the other four species used for PERMANOVA analyses, we found significant negative effects of natural florivory on the total amount of scent emitted by the damaged flower (Fig. 5, see Tables S4 and S5 for detailed statistics, PERMANOVA ‘Treatment*Sampling moment’).

**Fig. 6:**
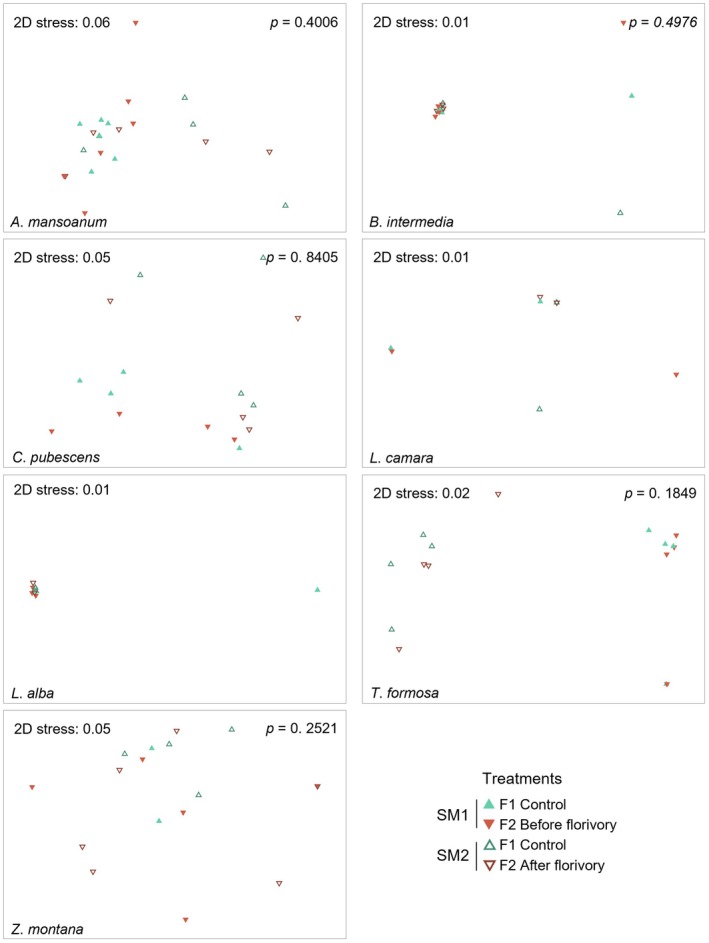
Non‐metric multidimensional scaling (NMDS) of floral scent samples collected from control and naturally damaged flowers/inflorescences at Sampling moment 1 and 2 (SM1 and SM2), based on Bray–Curtis similarities calculated on the relative amount of scent compounds. Control flowers/inflorescences were intact at both SM1 and SM2, fed flowers were intact at SM1, but naturally damaged by their respective florivores at SM2. The *p*‐values of the ‘Treatment × Sampling moment’ interaction effect obtained by PERMANOVA analyses are indicated.

We did not find an effect of natural florivory on the relative composition of floral scent in the five plant species analysed (*P* > 0.05, Fig. 6, see Table S4 for detailed statistics, PERMANOVA ‘Treatment*Sampling moment’). In *L*. *camara* and *L*. *alba*, for which the sample sizes were too small for performing robust PERMANOVA analyses, the NMDS does not show an obvious separation of the damaged flowers from those that were not damaged, regardless of the passage of time (Fig. 6).

We found that the relative amount of individual scent compound did not vary in *A*. *mansoanum*, *B*. *intermedia*, *L*. *camara* and *Z*. *montana* (*P* > 0.05, see Table S6 for detailed statistics). However, in the other three studied plant species, several scent compounds varied among flowers within a plant individual and/or were affected by the passage of time. In *C*. *pubescens*, 2‐phenylethanol showed lower relative amounts in SM2 than in SM1, regardless of florivory damages, whereas 4‐methoxyphenylethyl alcohol was higher in Control flowers regardless of sampling moment (Table S6). In *L*. *alba*, *(E)*‐linalool oxide furanoid was higher in SM2 flowers regardless of florivory (Table S6). In *T*. *formosa*, we detected that *allo*‐Ocimene varied among flowers within a plant individual and was affected by the passage of time, being lower in SM2 than in SM1, and lower in Control flowers. Several other compounds were affected by the passage of time, being that the relative amount of *syn*‐ and *anti*‐2‐methylbutylaldoxime, 1‐hexanol, methyl nicotinate, and (*E*)‐linalool oxide pyranoid increased with the passage of time; whereas methyl benzoate, 2,2,6‐trimethy‐6‐vinyldihydro‐2H‐pyran‐3(4H)‐one, and (*Z*)‐linalool oxide pyranoid decreased with the passage of time (Table S6). Moreover, the only compound that was specifically affected by florivory damages was 6‐methyl‐5‐hepten‐2‐one, which increased significatively in flowers damaged by florivores (Tables S6 and S7).

## DISCUSSION

The evaluated bee‐, butterfly‐, hawkmoth‐ and hummingbird‐pollinated species had variable areas of the corolla with scent‐emitting features. Florivores removed parts of these areas in all plant species, except in *L*. *camara*. The six species, from which corolla tissue with scent‐emitting features was removed, produced and emitted terpenes, aliphatic and aromatic compounds, and contrary to our expectations, the removal of corolla areas with scent‐emitting features neither reduced the total amount nor changed the relative composition of the floral scent in most species. Only *T*. *formosa*, which presented a few small holes in the areas corresponding to typical osmophores, emitted less scent after florivory, although scent composition remained similar.

The cells from tissues with scent‐emitting features were recognized by their relatively larger size compared with adjacent cells, thin cuticle, and dense cytoplasm, typical of volatile‐secreting cells and tissues (Vogel 1990). In addition to epidermal cells, we detected floral secretory trichomes in several species that showed a positive reaction for the presence of terpenoids. Floral secretory trichomes with scent‐emitting anatomical and cellular characteristics have been described in different families, including Bignoniaceae (Machado *et al*. 2006; Guimarães *et al*. 2008), Fabaceae (Marinho *et al*. 2014, 2016) and Orchidaceae (Vogel 1990; Adachi & Machado 2020), suggesting that these trichomes can participate in floral scent emission in the studied species. Terpenes, which are often responsible for the characteristic floral scent of a given species (Vogel 1983; Knudsen *et al*. 2006; Knudsen & Gershenzon 2020) were the main class of substances detected histochemically in the epidermis and secretory trichomes (Fig. 4A–D,F), and also in scent chemical analysis of the studied species. It is interesting to note that the main classes of substances detected by histochemical tests were indeed found in the floral scent through our chemical analytical analyses.

Floral scent composition was consistent with literature data for other bee‐, butterfly‐, and hawkmoth‐pollinated species, with floral scents of bee‐pollinated species dominated by terpenoids together with aromatic and aliphatic compounds; whereas butterfly‐pollinated species were dominated by benzenoids and terpenoids; and the hawkmoth‐pollinated species presented aromatic and N‐bearing compounds and terpenoids (Andersson & Dobson 2003; Dobson 2006). Additionally, the hummingbird‐pollinated species presented mainly terpenoids and a few aliphatic and aromatic compounds, presenting some compounds in common with other hummingbird‐pollinated species (Knudsen *et al*. 2004; Dellinger *et al*. 2019; Tunes *et al*. 2022). The area of the corolla removed by florivores was variable among species, ranging from 1% to 30%, and consequently, the corolla area bearing tissues with scent‐emitting features that was consumed by florivores was also variable among species, from 0% to 15%.

In the bee‐pollinated species, *A*. *mansoanum* and *C*. *pubescens*, the corolla area with scent‐emitting features removed by florivores corresponded to approximately 8% and 10%, respectively, which could be not enough to detect a potential reduction in the total amount of scent emitted by damaged flowers, assuming that the magnitude of response to florivory is dependent on the area associated to scent emission that was consumed. In *B*. *intermedia*, it was not possible to sample individual flowers; we sampled inflorescences containing from seven to 23 flowers and three to 17 floral buds. At the ‘Sampling moment 2’, we dealt with a scenario in which we have flowers consumed by florivores (ranging from one to four flowers per inflorescence) and new flowers that opened in the bagged inflorescence during the experiment (ranging from one to five). It is possible that any local effect of florivory on the damaged flower was surmounted by the scent emission of the newly opened flowers in the inflorescence. Thus, the results for this species would only allow us to identify a short‐term systemic response to florivory, which was not confirmed by our data.

The butterfly‐pollinated species presented very different levels of florivory. In *L*. *alba*, approximately 30% of the inflorescence and 15% of the stained portion of the inflorescences were fed on by florivores, whereas in *L*. *camara*, less than 1% of the inflorescence and 0% of the area of the corolla with scent‐emitting features were consumed by florivores. The remarkably low levels of florivory in *L*. *camara* are probably due to the presence of constitutive defensive compounds, such as anthocyanins (Goh *et al*. 2019), due to the massive presence of trichomes (Silva *et al*. 2016; Tozin *et al*. 2016), and due to the presence of toxic compounds, some with high insecticidal activity, which are present in leaf tissues (Kato‐Noguchi & Kato 2025) and could potentially be present in flower tissue as well. Although we did not have a large enough sample size for *L*. *camara* to support a statistical analysis, our data suggest that there was no response of *L*. *camara* floral scent to florivory, similar to what Goh *et al*. (2019) found, which was expected as florivores did not feed on corolla areas with scent‐emitting features. The maintenance of intact areas of the corolla with scent‐emitting features in this species could be important for pollinators to locate floral resource, as they form a ‘scent ring’ in the entrance of the corolla tube, which may act as a chemical guide, marking the path to the nectar (Dötterl & Jürgens 2005; Lawson *et al*. 2017; García *et al*. 2021). In *L*. *alba*, the caterpillars fed mostly on the central portions of the inflorescence, which comprised bracts and floral buds, and only on some parts of a few surrounding flowers. Thus, most of the functional flowers in the inflorescence remained intact and they may be responsible for the maintenance of floral scent amount and composition, considering that the peak of volatile emission usually occurs at the beginning of anthesis (Knudsen & Gershenzon 2020).

The only sampled hawkmoth‐pollinated species, *T*. *formosa*, showed low levels of florivory, corresponding to approximately 3% of the flower area corresponding to typical osmophores. For strongly scented flowers, such as *T*. *formosa*, it has been suggested that low levels of florivory indicate the presence of deterrent volatile compounds in floral scent (Kessler & Halitschke 2009 and references therein). There is no evidence that the main compounds in the scent of *T*. *formosa* (benzyl alcohol, methyl benzoate, (*E*)‐linalool oxide pyranoid) are repellent to florivores, however other aromatic compounds and monoterpenoids have been previously shown to repel florivores (Dötterl & Gershenzon 2023). In this species, florivory reduced the total amount of scent emitted by flowers by ~2.7‐fold, showcasing a local response triggered by florivory, as the flowers that were not consumed by florivores did not show the same reduction in scent amount, despite showing some reduction due to the passage of time. This response seems to not be area‐dependent, as *T*. *formosa*, the only species that showed a response to florivory, had a markedly smaller corolla area removed (2–3%), when compared with species that did not show a reduction in the total amount of floral scent, that is, *B*. *intermedia* (9–10%, bee‐pollinated), *C*. *pubescens* (8–14%, bee‐pollinated), *L*. *alba* (10–15%, butterfly‐pollinated), and *Z*. *montana* (12–15%, hummingbird‐pollinated). The reduction in scent emission in attacked *T*. *formosa* flowers could make them less conspicuous to florivores, minimizing the cost of florivory through a plant physiological response (Kessler *et al*. 2013; Boachon *et al*. 2015). On the other hand, this reduction in scent amount could also make the attacked flowers less attractive to pollinators, as higher amounts of floral scent are related to higher plant reproductive success (Majetic *et al*. 2009).

Finally, the hummingbird‐pollinated species, *Z*. *montana*, showed tissues with scent‐emitting features partially removed by florivores. Although hummingbirds have historically been regarded as not presenting a well‐developed sense of smell (Bang & Cobb 1968), recent studies have shown that they can detect (Steiger *et al*. 2008; Wester & Lunau 2017) and respond (Kessler *et al*. 2008) to floral scent, so the importance of the maintenance of scent emission in *Z*. *montana* flowers cannot be disregarded. Despite florivores having removed a moderate area bearing tissues with scent‐emitting features, the total amount of scent emitted and its composition remained similar in both scenarios, as reported for another hummingbird‐pollinated species (Tunes *et al*. 2022).

In the present study, the only species that showed any post‐florivory scent change was *T*. *formosa*. However, differently from what is reported for damaged vegetative organs (Kessler & Baldwin 2001; Perreca *et al*. 2020), we did not observe the induction of specific volatiles associated with plant defence in damaged flowers of *T*. *formosa* (as is true for the other six studied plant species as well). The absence of an induced chemical response to florivory, observed in most plant species, does not seem to be associated to plant species mating systems, because species that are exclusively allogamous, predominantly allogamous and have mixed mating systems showed similar results. Moreover, the only plant species that showed an effect of florivory on floral scent was one out of the four exclusively allogamous species. Moreover, this absence of induced responses cannot be attributed to any lack of biochemical or cellular machinery in terms of biosynthetic pathways, as we detected floral volatiles from every biosynthesis pathway that is also involved in the synthesis of leaf herbivory‐induced plant volatiles (Dudareva *et al*. 2004, 2013; Mumm & Dicke 2010). These results might suggest that leaves work differently from flowers in terms of short‐term herbivory response. Considering that the volatile synthesis is localized in specific floral tissues (Effmert *et al*. 2006; Dudareva *et al*. 2013; Knudsen & Gershenzon 2020) and that all biosynthesis pathways are present in those flowers, there is no feasible explanation for the absence of short‐term local response that we found here, other than that plants would not invest in defending such an ephemerous organ as a flower.

Besides the plants' ability to synthesize new scent compounds, herbivores can secrete salivary proteins that can inhibit or stimulate plant defences (Ma *et al*. 2024 and references therein). It could be argued that the absence of changes in scent due to florivory could be attributed to florivores that secrete salivary effectors that can attenuate the induction of jasmonic acid (JA) and salicylic acid (SA) pathways (Felton *et al*. 2014), reducing the potential plant responses to herbivory (Ruan *et al*. 2019 and references therein; Ma *et al*. 2024 and references therein). Such salivary effectors are common in many Lepidopteran species (Diezel *et al*. 2009) and sap suckers (Naessens *et al*. 2015; Su *et al*. 2019; Dong *et al*. 2020). However, in our study, only *L*. *alba* was fed on by Lepidopteran caterpillars and the other species were fed on by beetles or grasshoppers. Therefore, the absence of floral scent change in most of the studied plant species can likely not be attributed to salivary effectors of the florivores. As there are also no changes in floral scent composition, not even in the only plant species that showed an induced response to florivory, we cannot argue that the florivores might have secreted salivary elicitor proteins, which would have stimulated plant defence via JA and SA pathways, and Ca^2+^ influx, for example (Erb & Reymond 2019; Ma *et al*. 2024 and references therein).

Moreover, the florivores were left eating the flowers for 6–25 h, depending on the plant species (Table S1), which means that there was a substantial interval between when florivores started feeding on the flowers (1 h after the first scent sampling) and when we sampled floral scent after florivory. The species that were left with florivores for less than 24 h were *A. mansoanum*, *T. formosa* and *Z. montana*. However, *T*. *formosa* was the only species that showed any floral scent change, despite not being exposed to florivores for such a long time. Though one could argue that *A*. *mansoanum* and *Z*. *montana* could need more time to show any potential scent change due to florivory, studies show that plants can start to show responses from 5 min to 4 h after damage by herbivores (Schmelz *et al*. 2001; Lin *et al*. 2021), lasting for up to 48 h (Lin *et al*. 2021), and flowers also show response after 4 h of the application of JA (Jiang *et al*. 2011). Moreover, the flower of both of these species are only functional for approximately 24 h. Therefore, to affect pollination, florivory would have to have induced floral scent changes within this timeframe.

Most species did not show any change in individual scent compounds due to time and florivory, which was expected as they showed no changes in scent composition and total scent emission. *Centrosema pubescens* and *L*. *alba* showed changes in two scent compounds due to the passage of time, as was expected especially in *C*. *pubescens*, which showed a general decrease in total scent emission in SM2. This was likely due to the decrease in the relative amount of 2‐phenylethanol from SM1 to SM2. This benzenoid is a widespread floral scent compound (Knudsen *et al*. 2006; Knudsen & Gershenzon 2020) and elicits physiological and/or attractive responses in various pollinators (Dötterl & Gershenzon 2023). In *T*. *formosa*, several common and widespread floral scent compounds and one less common compound (Knudsen *et al*. 2006; Knudsen & Gershenzon 2020) also suffered changes in the relative importance between Sampling moments 1 and 2, which also reflects natural changes of this plant species and are not associated with florivory damages. However, the increase in relative importance of the common irregular terpene 6‐methyl‐5‐hepten‐2‐one (Knudsen *et al*. 2006; Knudsen & Gershenzon 2020), induced by florivory, might have relevant ecological roles, as this compound has been reported as being increased or induced by leaf herbivory (Tang *et al*. 2012; Yu *et al*. 2018; but see Dötterl & Gershenzon 2023) and by sap sucking (Higashida *et al*. 2022), and has also been reported as being associated with the attraction of parasitoids of herbivores (Yu *et al*. 2018). Therefore, it is possible that the change in this specific scent compound elicited by florivory might attract natural enemies of the florivores, while potentially increasing chemical signalling to pollinators, as this scent compound is attractive to moths (Tang *et al*. 2012; Li *et al*. 2024), which are the pollinators of this plant species.

In summary, we verified a very restricted effect of florivory on floral scent, with only one species having its total amount of scent negatively affected. Most plants did not show short‐term local chemical responses induced by the action of the florivores, which raises questions about how rare florivory‐induced responses are and how species‐specific those can be. Furthermore, our findings indicate that after damage caused by florivores, the scent blend of individual flowers remains similar, even in the hawkmoth‐pollinated species, which showed no change in relative scent composition despite the reduction in the amount of scent emitted. This means that the pollinators of the other plant species cannot chemically distinguish intact from damaged flowers by scent amount and scent composition. These plants increase the olfactory signalling, which may keep pollinator visits constant despite the flower visual modifications caused by florivores. The results of the present study highlight that what we know so far in terms of chemical response to leaf herbivory cannot be directly transposed to florivory‐based interactions. Emitting defensive compounds in leaves can repel leaf herbivores and attract parasites/ parasitoids thereof, but in flowers, it would potentially lead to a side effect and end up repelling pollinators and not just florivores (Kessler & Halitschke 2009; Kessler 2015). Besides, there is evidence that in some plant species, florivory might elicit a systemic response, but this response involves only the leaves, not floral volatile emission (Goh *et al*. 2019), ensuring the maintenance of chemical communication pathways with pollinators.

In a broad perspective, our results suggest that floral scent traits seem to be more stable and less responsive to natural levels of florivory than what we previously supposed. This could be interpreted in an evolutionary context as a result of a trade‐off between attracting pollinators and emitting repellent compounds that could protect flowers from florivores, as florivory in angiosperms is older than the latest mass radiation of pollinator lineages (~50 Ma) (Peris & Condamine 2024) and floral traits might have been concomitantly under selective pressures by both pollinators and florivores.

## AUTHOR CONTRIBUTIONS

PT and EG conceived the study, collected, and analysed the data and wrote the main manuscript text. SD contributed to the scent analyses and commented on earlier versions of the manuscript. YC and SRM contributed with anatomical and histochemical analysis and description. All authors contributed critically to the manuscript and approved the final version.

## FUNDING INFORMATION

This work was supported by the São Paulo Research Foundation (FAPESP) [Grant #2018/14146‐0 and Grant #2009/17611‐7 to E.G., Grant #2021/10639‐5 to E.G. and P.T., Grant #2021/13392‐0 to S.R.M.]; by the National Council of Technological and Scientific Development (CNPq) [Proc. 312799/2021‐7 to E.G. and Proc. 308982/2020‐7 to S.R.M.]; and by the Coordination of Superior Level Staff Improvement, Brazil [Finance Code 001, PDSE #88882.433249/2018‐01 to P.T.].

## Supporting information


**Table S1.** Plant species, period of the day in which ‘Sampling period 1’ occurred, and time interval in which florivores fed on flowers. The period between brackets corresponds to single plants in which florivores fed on for less time.
**Table S2**. The sample size for each plant species, considering the number of intact and damaged flowers or inflorescences (indicated by an asterisk) sampled in each treatment in each sampling moment (SM). We also show the duration of each sample collection.
**Table S3**. Total absolute (mean ± SD; in ng.flower^−1^.h^−1^ or ng.inflorescence^−1^.h^−1^) and relative amount (mean ± SD; (minimum‐maximum); in %) of scent emitted by flowers of each plant species submitted to each treatment. Species for which scent is presented for the inflorescence are indicated by +. Scent compounds are listed according to their chemical class and linear Retention Index (RI) based on a series of n‐alkanes (C6–C20), *sensu* Van Den Dool & Kratz (1963). Compounds marked by an asterisk were identified based on their mass spectra and the RI of synthetic standards. SM1 = Sampling moment 1; SM2 = Sampling moment 2; tr = trace amount (mean < 0.1%).
**Table S4**. Detailed outcomes of PERMANOVA analyses that evaluated if florivory affected the total amount of scent and the relative composition of floral scent compounds in the study plant species. Random factors in the PERMANOVA design are marked in italics. Statistically significant results are marked in bold.
**Table S5**. Pairwise comparisons of the effect of ‘Treatment*Sampling moment’ for the total amount of scent of *Tocoyena formosa* flowers. Statistically significant results are marked in bold. SM: Sampling moment.
**Table S6**. Detailed outcomes of PERMANOVA analyses for the individual scent compounds in each plant species.
**Table S7**. Pairwise comparisons of the effect of ‘Treatment × Sampling moment’ for the relative amount of 6‐methyl‐5‐hepten‐2‐one in *Tocoyena formosa* flowers. Statistically significant results are marked in bold. SM, sampling moment.

## Data Availability

All relevant data are within the manuscript and Supporting Information S1.
