# Supplementary material for: Resilience of floral scent emission after florivory
Source: Plant Biol (Stuttg). 2026 Mar 25;28(5):1491–506. doi: 10.1111/plb.70209 (PMC13358683; doi:10.1111/plb.70209)
Supplement: Supplementary file 1 — Table S1. Plant species, period of the day in which ‘Sampling period 1’ occurred, and time interval in which florivores fed on flowers. The period between brackets corresponds to single plants in which florivores fed on for less time. Table S2. The sample size for each plant species, considering the number of intact and damaged flowers or inflorescences (indicated by an asterisk) sampled in each treatment in each sampling moment (SM). We also show the duration of each sample collection. Table S3. Total absolute (mean ± SD; in ng.flower−1.h−1 or ng.inflorescence−1.h−1) and relative amount (mean ± SD; (minimum‐maximum); in %) of scent emitted by flowers of each plant species submitted to each treatment. Species for which scent is presented for the inflorescence are indicated by +. Scent compounds are listed according to their chemical class and linear Retention Index (RI) based on a series of n‐alkanes (C6–C20), sensu Van Den Dool & Kratz (1963). Compounds marked by an asterisk were identified based on their mass spectra and the RI of synthetic standards. SM1 = Sampling moment 1; SM2 = Sampling moment 2; tr = trace amount (mean < 0.1%). Table S4. Detailed outcomes of PERMANOVA analyses that evaluated if florivory affected the total amount of scent and the relative composition of floral scent compounds in the study plant species. Random factors in the PERMANOVA design are marked in italics. Statistically significant results are marked in bold. Table S5. Pairwise comparisons of the effect of ‘Treatment*Sampling moment’ for the total amount of scent of Tocoyena formosa flowers. Statistically significant results are marked in bold. SM: Sampling moment. Table S6. Detailed outcomes of PERMANOVA analyses for the individual scent compounds in each plant species. Table S7. Pairwise comparisons of the effect of ‘Treatment × Sampling moment’ for the relative amount of 6‐methyl‐5‐hepten‐2‐one in Tocoyena formosa flowers. Statistically significant results are marked in bold. SM, s [file PLB-28-1491-s001.docx]

**Table S1.** Plant species, period of the day in which ‘Sampling period 1’ occurred, and time interval in which florivores fed on flowers. The period between brackets corresponds to single plants in which florivores fed on for less time.

| **Plant species** | **Sampling period (h)** | **Time interval in which florivores fed on flowers** |
| --- | --- | --- |
| *Amphilophium mansoanum* | 0700 - 1200 | 6h – 6h30m (1h20m) |
| *Byrsonima intermedia* | 0700 - 0930 | 23h – 26h |
| *Centrosema pubescens* | 1330 - 1800 | 22h – 25h (6h) |
| *Lantana* *camara* | 1430 - 1600 | 24h10m – 24h30m |
| *Lippia alba* | 1300 - 1500 | 24h |
| *Tocoyena formosa* | 1900 - 2130 | 13h – 15h |
| *Zeyheria montana* | 0700 - 0900 | 8h – 9h |

**Table S2.** The sample size for each plant species, considering the number of intact and damaged flowers or inflorescences (indicated by an asterisk) sampled in each treatment in each sampling moment (SM). We also show the duration of each sample collection.

| **Plant species** |  | **SM1 Control** | **SM2 Control** | **SM1 Before florivory** | **SM2 After florivory** | **Sampling duration (min)** |
| --- | --- | --- | --- | --- | --- | --- |
| *Amphilophium mansoanum* |  | 6 | 5 | 6 | 5 | 20 |
| *Byrsonima intermedia** |  | 4 | 3 | 4 | 3 | 40 |
| *Centrosema pubescens* |  | 4 | 4 | 4 | 4 | 40 |
| *Lantana camara** |  | 2 | 2 | 2 | 2 | 40 |
| *Lippia alba** |  | 3 | 2 | 3 | 2 | 60 |
| *Tocoyena formosa* |  | 4 | 4 | 4 | 4 | 15 |
| *Zeyheria montana* |  | 5 | 5 | 5 | 6 | 60 |

**Table S3.** Total absolute (mean ± sd; in ng.flower^-1^.hour^-1^ or ng.inflorescence^-1^.hour^-1^) and relative amount (mean ± sd; (minimum-maximum); in %) of scent emitted by flowers of each plant species submitted to each treatment. Species for which scent is presented for the inflorescence are indicated by +. Scent compounds are listed according to their chemical class and linear Retention Index (RI) based on a series of n-alkanes (C6-C20), *sensu* van Den Dool and Kratz (1963). Compounds marked by an asterisk were identified based on their mass spectra and the RI of synthetic standards. SM1 = Sampling moment 1; SM2 = Sampling moment 2; tr = trace amount (mean <0.1%).

| **Bee-pollinated plant species** | **RI** | ***A*. *mansoanum*** | | | | ***B.* *intermedia*** | | | | ***C*. *pubescens*** | | | |
| --- | --- | --- | --- | --- | --- | --- | --- | --- | --- | --- | --- | --- | --- |
|  |  | **Control** | | **Before florivory** | **After florivory** | **Control** | | **Before florivory** | **After florivory** | **Control** | | **Before florivory** | **After florivory** |
|  |  | **SM1** | **SM2** | **SM1** | **SM2** | **SM1** | **SM2** | **SM1** | **SM2** | **SM1** | **SM2** | **SM1** | **SM2** |
| Total number of compounds |  | 41 | 30 | 43 | 34 | 47 | 43 | 43 | 44 | 52 | 37 | 51 | 40 |
| N_samples_ (N_plants_) |  | 6 (5) | 5 (5) | 6 (5) | 5 (5) | 4 (4) | 3 (3) | 4 (4) | 3 (3) | 4 (4) | 4 (4) | 4 (4) | 4 (4) |
| Total amount of scent emitted (ng/ flower/ h) |  | 497.8 ± 127.2 | 278.3 ± 177.2 | 491.4 ± 298.0 | 444.5 ± 135.6 | 44.3 ± 50.8 | 39.4 ± 34.9 | 19.9 ± 19.3 | 34.1 ± 38.5 | 170.9 ± 83.0 | 122.1 ± 138.9 | 144.0 ± 134.0 | 162.6 ± 209.7 |
|  |  |  |  |  |  |  |  |  |  |  |  |  |  |
| **Aliphatic compounds** |  |  |  |  |  |  |  |  |  |  |  |  |  |
| 2-Butenal | 646 | - | - | 0.6 ± 1.6  (0-4.1) | 0.8 ± 1.8  (0-4.1) | - | - | - | - | - | - | - | - |
| 2-Butanyl acetate | 752 | - | - | 0.6 ± 1.5  (0-3.6) | 0.7 ± 1.6  (0-3.6) | - | - | - | - | - | - | - | - |
| 4-Methyl-3-pentene-2-ol* | 793 | - | - | - | - | - | - | - | - | 0.1 ± 0.3  (0-0.6) | 0.8 ± 0.9  (0-2.1) | 0.2 ± 0.4  (0-0.8) | 0.9 ± 1.2  (0-2.6) |
| Hexanal* | 796 | 0.5 ± 0.9  (0-2.3) | - | 1.8 ± 3.8  (0-9.4) | 0.5 ± 0.7  (0-1.7) | 2.1 ± 3.3  (0-7.1) | - | 0.9 ± 1.4  (0-3.0) | - | - | - | - | - |
| (*Z*)-2-Hexenal* | 852 | - | - | - | - | 0.4 ± 0.8  (0-1.6) | - | tr | - | - | - | - | - |
| 1-Methoxy-2-propyl acetate | 866 | - | - | - | - | 3.1 ± 6.3  (0-12.6) | - | 6.1 ± 12.3  (0-24.7) | - | - | - | - | - |
| Methyl hexanoate* | 922 | tr | 0.1 ± 0.2  (0-0.5) | tr | - | - | - | - | - | - | - | - | - |
| Methyl octanoate* | 1120 | - | - | - | - | 0.2 ± 0.4  (0-0.9) | - | tr | - | - | - | - | - |
| (*Z*)-3-Hexenyl butyrate* | 1188 | - | - | - | - | - | - | - | - | tr | 0.2 ± 0.5 (0-1.1) | tr | 0.5 ± 1.1 (0-2.3) |
| Ethyl octanoate* | 1195 | 0.9 ± 2.2  (0-5.6) | - | 1.3 ± 3.2  (0-8) | - | - | - | - | - | - | - | - | - |
| Methyl decanoate | 1322 | - | - | - | - | 0.3 ± 0.5  (0-1.1) | 10.3 ± 17.9  (0-31.0) | 1.4 ± 2.5  (0-5.1) | tr | tr | - | - | 0.1 ± 0.1  (0-0.4) |
| Tetradecane* | 1400 | - | - | - | - | 0.1 ± 0.2  (0-0.4) | 0.2 ± 0.4  (0-0.8) | - | 0.4 ± 0.7  (0-1.2) | - | - | - | - |
| Methyl tetradecanoate | 1723 | - | - | - | - | - | - | - | - | tr | tr | tr | 3.6 ± 6.8  (0-13.9) |
| Methyl pentadecanoate | 1824 | - | - | - | - | - | - | - | - | tr | - | - | tr |
| **Aromatic compounds** |  |  |  |  |  |  |  |  |  |  |  |  |  |
| Benzaldehyde* | 963 | - | - | - | - | - | tr | - | 0.1 ± 0.2  (0-0.4) | - | - | - | - |
| Benzyl alcohol* | 1036 | - | - | - | - | - | tr | - | tr | 1.0 ± 0.7  (0-1.6) | 0.1 ± 0.2  (0-0.4) | 1.7 ± 1.8  (0-4.3) | 0.1 ± 0.2  (0-0.5) |
| Methyl phenethylether | 1086 | - | - | - | - | - | - | - | - | tr | tr | tr | tr |
| 2-Phenylethanol* | 1117 | 0.6 ± 0.6  (0-1.4) | 0.2 ± 0.3  (0-0.7) | 0.5 ± 0.5  (0-1.3) | - | - | - | - | - | 63.9 ± 21.3  (33.1-79.8) | 32.9 ± 13.7  (16.8-50.3) | 54.0 ± 19.9  (31.6-75.3) | 34.2 ± 18.4  (10.5-55.5) |
| 1,2-Dimethoxybenzene* | 1145 | - | - | - | - | - | - | - | - | 2.0 ± 1.1  (0.9-3.6) | 1.1 ± 0.9  (0-2.1) | 1.3 ± 0.8  (0.7-2.5) | 1.0 ± 0.8  (0-2.0) |
| 2-Phenylethyl formate* | 1180 | - | - | - | - | - | - | - | - | tr | - | tr | - |
| Methyl salicylate* | 1204 | - | - | - | - | 0.1 ± 0.3  (0-0.6) | - | 0.1 ± 0.2  (0-0.5) | - | - | - | - | - |
| 2-Phenylethyl acetate* | 1262 | - | - | - | - | - | - | - | - | 0.2 ± 0.2  (0-0.5) | - | tr | - |
| *p*-Anisaldehyde* | 1263 | - | - | - | - | 0.3 ± 0.6  (0-1.2) | - | 0.3 ± 0.7  (0-1.5) | - | tr | 0.3 ± 0.5  (0-1.2) | tr | 0.1 ± 0.2  (0-0.4) |
| *p*-Anisyl alcohol* | 1288 | - | - | - | - | - | - | - | - | - | tr | - | tr |
| *p*-Propylanisole | 1307 | - | - | - | - | - | - | - | - | tr | 0.2 ± 0.4  (0-0.8) | tr | 0.1 ± 0.3  (0-0.7) |
| Anisyl formate | 1341 | - | - | - | - | - | - | - | - | 0.1 ± 0.1  (0-0.2) | 0.4 ± 0.5  (0-1.2) | tr | 0.2 ± 0.3  (0-0.6) |
| Eugenol* | 1366 | 0.1 ± 0.3  (0-0.8) | tr | 0.3 ± 0.4  (0-1.1) | 0.1 ± 0.2  (0-0.5) | - | - | - | - | - | - | - | - |
| 4-Methoxyphenylethyl alcohol* | 1375 | - | - | - | - | - | - | - | - | 8.9 ± 3.7  (4.7-13.7) | 32.2 ± 17  (15.8-49.6) | 3.5 ± 3.2  (0-7.4) | 21.7 ± 15.9  (4.9-39.3) |
| (*E*)-Methylcinnamate* | 1392 | - | - | - | - | - | - | - | - | - | tr | 0.3 ± 0.7  (0-1.5) | - |
| Benzyl 2-methylbutanoate | 1394 | - | - | - | - | - | - | - | - | tr | - | tr | - |
| Benzyl isovalerate* | 1400 | - | - | - | - | - | - | - | - | tr | 0.3 ± 0.7  (0-1.4) | tr | tr |
| Benzyl tiglate* | 1506 | - | - | - | - | - | - | - | - | 0.2 ± 0.4  (0-0.9) | - | tr | - |
| 4-Methoxyphenylethyl acetate* | 1514 | - | - | - | - | - | - | - | - | tr | 0.1 ± 0.2  (0-0.4) | tr | 0.2 ± 0.4  (0-0.8) |
| Benzyl benzoate* | 1785 | - | - | - | - | - | - | - | - | tr | - | tr | - |
| **N-bearing compounds** |  |  |  |  |  |  |  |  |  |  |  |  |  |
| Phenylacetonitrile* | 1145 | - | - | - | - | 0.7 ± 1.0  (0-2.2) | 1.0 ± 1.3  (0-2.6) | 1.6 ± 2.4  (0-5.2) | 0.7 ± 0.7  (0-1.4) | - | - | - | - |
| 3-Phenylpropanenitrile | 1246 | - | - | - | - | - | - | - | - | tr | - | tr | - |
| Indole* | 1303 | tr | - | tr | 1.7 ± 3.9  (0-8.7) | tr | tr | - | tr | tr | - | tr | - |
| **S-bearing compounds** |  |  |  |  |  |  |  |  |  |  |  |  |  |
| Dimethyldisulfide* | 741 | - | - | - | - | - | 0.2 ± 0.3  (0-0.6) | 0.2 ± 0.5  (0-1) | 0.2 ± 0.4  (0-0.7) | - | - | - | - |
| **Terpenoids** |  |  |  |  |  |  |  |  |  |  |  |  |  |
| a 2-Methyl-2-vinyl-5-hydroxytetrahydrofuran | 928 | - | - | - | - | - | - | - | - | 0.2 ± 0.3  (0-0.6) | 0.3 ± 0.5  (0-1.1) | 0.2 ± 0.5  (0-1.1) | 0.1 ± 0.1  (0-0.4) |
| α-Pinene* | 939 | - | - | - | 0.1 ± 0.2  (0-0.5) | - | tr | tr | tr | - | - | - | - |
| a 2-Methyl-2-vinyl-5-hydroxytetrahydrofuran | 946 | - | - | - | - | - | - | - | - | 0.1 ± 0.1  (0-0.3) | 0.1 ± 0.3  (0-0.6) | 0.2 ± 0.4  (0-0.8) | tr |
| β-Citronellene* | 946 | - | - | - | - | - | tr | tr | tr | - | - | - | - |
| β-Pinene* | 979 | 0.1 ± 0.2  (0-0.7) | - | 0.1 ± 0.4  (0-1.0) | - | - | - | - | - | - | - | - | - |
| 6-Methyl-5-hepten-2-one* | 987 | - | - | - | - | - | 0.3 ± 0.6  (0-1.1) | - | 0.9 ± 1.6  (0-2.9) | - | - | - | - |
| β-Myrcene* | 990 | 1.5 ± 1.2  (0-2.8) | 0.7 ± 1.0  (0-2) | 0.8 ± 1.0  (0-2.2) | 2.3 ± 2.6  (0-6.3) | - | - | - | - | 0.2 ± 0.1  (0-0.4) | 0.4 ± 0.5  (0-1.1) | 2.0 ± 2.1  (0.1-4.9) | 0.2 ± 0.2  (0-0.6) |
| δ-3-Carene* | 1008 | - | - | - | - | - | - | - | - | tr | - | tr | - |
| Limonene* | 1036 | - | - | - | - | - | - | - | - | tr | - | tr | - |
| (*Z*)-β-Ocimene* | 1037 | 9.6 ± 1.9  (7.3-12.5) | 5.9 ± 4.2  (0-10.5) | 11.1 ± 4.8  (6.5-18.1) | 8.3 ± 6.1  (3.1-18) | 0.4 ± 0.8  (0-1.7) | - | 0.1 ± 0.2  (0-0.5) | - | 0.3 ± 0.5  (0-1.1) | - | 0.9 ± 1.0  (0-2.4) | - |
| Eucalyptol* | 1040 | - | - | - | - | tr | tr | tr | tr | - | - | - | - |
| Lavender lactone* | 1044 | - | - | - | - | - | - | - | - | 0.1 ± 0.2  (0-0.5) | 0.3 ± 0.5  (0-1.1) | 0.1 ± 0.3  (0-0.6) | 0.3 ± 0.3  (0-0.8) |
| (*E*)-β-Ocimene* | 1049 | 64.9 ± 2.5  (61.7-68.3) | 51.7 ± 18.0  (32.9-76.2) | 56.2 ± 15.4  (29.9-74.0) | 54.2 ± 17.9  (30-73.8) | 1.6 ± 3.3  (0-6.6) | 0.4 ± 0.8  (0-1.4) | 0.9 ± 1.8  (0-3.6) | 0.2 ± 0.4  (0-0.7) | 2.5 ± 4.9  (0-9.9) | 0.1 ± 0.3  (0-0.6) | 8.4 ± 9.9  (0-21.2) | 0.5 ± 0.7  (0-1.5) |
| γ-Terpinene* | 1063 | - | - | - | - | - | - | - | - | tr | - | tr | - |
| (*Z*)-Linalool oxide (furanoid)* | 1076 | tr | - | - | 0.4 ± 1.1  (0-2.4) | 0.2 ± 0.5  (0-1.1) | tr | 0.1 ± 0.2  (0-0.5) | tr | 0.2 ± 0.2  (0-0.5) | 0.6 ± 0.6  (0-1.5) | 0.2 ± 0.3  (0-0.5) | 0.4 ± 0.3  (0-0.9) |
| (*E*)-Linalool oxide (furanoid)* | 1092 | - | 0.1 ± 0.3  (0-0.7) | tr | 0.7 ± 1.0  (0-2.4) | tr | - | tr | - | 0.2 ± 0.2  (0-0.6) | 0.3 ± 0.3  (0-0.8) | 0.1 ± 0.1  (0-0.3) | 0.2 ± 0.2  (0-0.6) |
| α-Terpinolene* | 1094 | - | - | - | - | - | - | - | - | tr | - | tr | - |
| (*Z*)-4,8-Dimethyl-1,3,7-nonatriene | 1099 | - | - | - | - | 0.3 ± 0.7  (0-1.5) | - | 0.1 ± 0.2  (0-0.4) | - | - | - | - | - |
| Linalool* | 1099 | 1.3 ± 3.3  (0-8.1) | 20.5 ± 27.6  (0-67) | 2.1 ± 2.6  (0-6.9) | 15 ± 19.5  (0-40.7) | 1.7 ± 3.5  (0-7) | 0.1 ± 0.2  (0-0.3) | 0.6 ± 1.2  (0-2.5) | tr | 16.5 ± 19.1  (0.07-44.1) | 24.9 ± 21.2  (0-45.5) | 23.3 ± 16.9  (0.2-36.3) | 32.0 ± 22.3  (0-49.8) |
| Hotrienol | 1104 | - | - | - | - | - | - | - | - | - | 0.1 ± 0.3  (0-0.6) | - | 0.1 ± 0.3  (0-0.6) |
| (*E*)-4,8-dimethyl-1,3,7-nonatriene* | 1119 | - | - | - | - | 2.3 ± 4.6  (0-9.4) | tr | 1.0 ± 2.1  (0-4.2) | tr | - | - | - | - |
| Ocimene derivative | 1125 | 0.2 ± 0.3  (0-0.7) | 3 ± 5.8  (0-13.4) | 0.8 ± 1.1  (0-2.8) | 0.3 ± 0.3  (0-0.6) | - | tr | - | tr | - | - | - | - |
| *allo*-Ocimene* | 1130 | 1.3 ± 1.6  (0-3.3) | 3.2 ± 3.7  (0-8.1) | 1.9 ± 1.6  (0-3.2) | 2.1 ± 1.4  (0-3.8) | 0.2 ± 0.4  (0-0.8) | tr | tr | tr | 0.1 ± 0.2  (0-0.4) | - | 0.1 ± 0.08  (0-0.2) | tr |
| *p*-1,3,8-Menthatriene | 1134 | 5.4 ± 4.0  (0-9.6) | 5.6 ± 3.9  (0-10.2) | 6.8 ± 7.6  (0-17.8) | 3.3 ± 2.4  (0-5.9) | 0.2 ± 0.4  (0-0.8) | 0.1 ± 0.1  (0-0.3) | 0.1 ± 0.2  (0-0.5) | tr | 0.2 ± 0.4  (0-0.8) | tr | 0.3 ± 0.3  (0-0.6) | tr |
| (*E*)-Ocimene epoxide* | 1143 | tr | - | tr | 0.1 ± 0.1  (0-0.2) | - | - | - | - | - | - | - | - |
| *neoallo*-Ocimene* | 1145 | tr | tr | 0.1 ± 0.3  (0-0.7) | - | - | - | - | - | - | - | tr | - |
| (*Z*)-linalool oxide (pyranoid) | 1175 | - | - | - | - | 0.1 ± 0.2  (0-0.5) | tr | - | tr | tr | tr | tr | 0.1 ± 0.1  (0-0.2) |
| (*E*)-linalool oxide (pyranoid)* | 1175 | - | - | - | - | - | - | - | - | tr | tr | tr | 0.1 ± 0.1  (0-0.4) |
| α-Terpineol* | 1198 | - | - | - | - | - | - | - | - | tr | tr | tr | tr |
| Geraniol | 1255 | - | - | - | - | - | - | - | - | tr | - | tr | - |
| Neodihydrocarveol | 1286 | - | - | - | - | 0.1 ± 0.2  (0-0.5) | - | 0.4 ± 0.9  (0-1.8) | - | - | - | - | - |
| (*Z*)-Dihydrocarveol | 1287 | - | - | - | - | tr | tr | - | tr | - | - | - | - |
| Lavandulyl acetate* | 1290 | - | - | - | - | 0.1 ± 0.2  (0-0.5) | - | tr | - | - | - | - | - |
| Chrysanthenone | 1317 | - | - | - | - | tr | tr | - | tr | - | - | - | - |
| β-Bourbonene* | 1407 | - | tr | tr | - | - | - | - | - | - | - | - | - |
| β-Elemene | 1408 | - | - | - | - | tr | 0.1 ± 0.2  (0-0.4) | tr | 0.1 ± 0.1  (0-0.3) | - | - | - | - |
| (*E*)-β-Caryophyllene* | 1444 | 0.1 ± 0.2  (0-0.4) | 0.2 ± 0.3  (0-0.7) | tr | tr | 70.9 ± 42.7  (6.9-94.7) | 58.7 ± 50.8  (0-89.7) | 69.2 ± 45.1  (1.5-94.1) | 90.9 ± 4.9  (87.4-96.6) | - | - | - | - |
| Dihydro-ß-Ionone* | 1452 | 1.4 ± 1.5  (0-3.4) | 0.1 ± 0.2  (0-0.5) | 1.5 ± 2.2  (0-6.0) | 0.3 ± 0.5  (0-1.1) | - | - | - | - | - | - | - | - |
| α-Humulene* | 1481 | - | - | - | - | 10.2 ± 13.1 (3.5-29.9) | 25.8 ± 37.3 (4.1-68.9) | 4.2 ± 4.6 (0-10.8) | 3.9 ± 0.6 (3.2-4.4) | - | - | - | - |
| β-Ionone* | 1499 | 2.9 ± 2.3  (0-6.7) | 0.8 ± 1.1  (0-2.7) | 2.1 ± 2.5  (0-6.8) | 1.3 ± 1.6  (0-3.2) | - | - | - | - | tr | tr | tr | tr |
| Germacrene D* | 1497 | - | - | - | - | - | tr | - | tr | - | - | - | - |
| (*E*,*E*)-α-Farnesene* | 1512 | 0.1 ± 0.1  (0-0.4) | tr | tr | 0.4 ± 0.7  (0-1.7) | - | - | - | - | - | - | - | - |
| (*Z*)-Nerolidol* | 1536 | - | - | - | - | 0.4 ± 0.8  (0-1.8) | tr | 0.1 ± 0.2  (0-0.4) | tr | - | - | - | - |
| Caryophyllene oxide derivative | 1582 | - | - | - | - | tr | tr | - | tr | - | - | - | - |
| Kessane | 1705 | - | - | - | - | tr | tr | 9.6 ± 19.2  (0-38.5) | 0.1 ± 0.1  (0-0.2) | - | - | - | - |
| **Unidentified compounds** |  |  |  |  |  |  |  |  |  |  |  |  |  |
| m/z: 82,67,43,82,71 | 646 | - | - | - | - | tr | 0.5 ± 0.9  (0-1.6) | 0.7 ± 1.4  (0-2.9) | 0.4 ± 0.7  (0-1.3) | - | - | - | - |
| m/z: 43,57,84,55,71 | 779 | - | - | - | - | - | - | - | - | tr | tr | tr | tr |
| m/z: 95,67,41,53,81 | 890 | 0.1 ± 0.3  (0-0.8) | 0.3 ± 0.4  (0-1) | 0.7 ± 0.6  (0-1.5) | - | - | - | - | - | - | - | - | - |
| m/z: 43,70,57,84,96 | 902 | - | - | - | - | tr | - | tr | - | - | - | - | - |
| m/z: 56,55,40,73,105 | 929 | - | - | - | - | - | tr | tr | tr | - | - | - | - |
| m/z: 43,71,115,55,83 | 954 | - | - | - | - | - | - | - | - | tr | - | tr | - |
| m/z: 93,77,41,55,136 | 958 | - | - | - | - | tr | tr | tr | tr | - | - | - | - |
| m/z: 105,40,56,120,70 | 970 | - | - | - | - | - | tr | - | tr | - | - | - | - |
| m/z: 55,70,94,83,43 | 977 | 0.6 ± 1.5  (0-3.7) | - | 1.6 ± 3.9  (0-9.6) | - | - | - | - | - | - | - | - | - |
| m/z: 57,43,105,71,85 | 999 | 0.1 ± 0.3  (0-0.7) | - | 0.1 ± 0.2  (0-0.6) | - | - | - | - | - | - | - | - | - |
| m/z: 91,119,134,77,105 | 1010 | 1.1 ± 1.2  (0-2.7) | 1.5 ± 1.5  (0-3.3) | 2.2 ± 3.1  (0-7.9) | 1.1 ± 0.7  (0.3-2.2) | - | - | - | - | - | - | - | - |
| m/z: 43,91,119,134,77 | 1012 | - | - | - | - | - | tr | - | tr | - | - | - | - |
| m/z: 81,41,69,79,150 | 1015 | - | - | - | - | - | - | - | - | 0.2 ± 0.1  (0-0.4) | 0.2 ± 0.1  (0-0.4) | 0.3 ± 0.2  (0-0.5) | 0.2 ± 0.1  (0-0.4) |
| m/z: 81,44,79,53,60 | 1023 | tr | - | tr | tr | - | - | - | - | - | - | - | - |
| m/z: 77,57,43,107,121 | 1026 | - | - | - | - | tr | - | tr | - | - | - | - | - |
| m/z: 57,43,70,83,91 | 1060 | - | - | - | - | 0.3 ± 0.7  (0-1.4) | - | tr | - | - | - | - | - |
| m/z: 69,55,41,109,85 | 1079 | tr | tr | tr | tr | - | - | - | - | - | - | - | - |
| m/z: 91,119,134,41,77 | 1082 | 0.4 ± 0.4  (0-0.9) | 0.2 ± 0.3  (0-0.7) | 0.5 ± 0.5  (0-1.5) | 0.2 ± 0.2  (0-0.5) | - | - | - | tr | - | - | - | - |
| m/z: 55,70,41,83,119 | 1163 | 0.8 ± 0.8  (0-2) | 0.5 ± 0.9  (0-2) | 0.9 ± 1.2  (0-3) | 0.4 ± 0.4  (0-0.9) | - | - | - | - | - | - | - | - |
| m/z: 95,93,41,123,150 | 1168 | 0.1 ± 0.2  (0-0.5) | - | 0.1 ± 0.4  (0-1.1) | - | - | - | - | - | - | - | - | - |
| m/z: 59,94,79,43,105 | 1174 | 0.9 ± 0.6  (0-1.6) | 0.7 ± 0.7  (0-1.6) | 0.5 ± 0.5  (0-1.3) | 0.7 ± 0.5  (0-1.5) | - | - | - | - | - | - | - | - |
| m/z: 95,150,41,79,55 | 1183 | 0.3 ± 0.3  (0-1) | 0.3 ± 0.4  (0-1) | 0.3 ± 0.3  (0-0.8) | 0.2 ± 0.2  (0-0.4) | - | - | - | - | - | - | - | - |
| m/z: 82,67,85,43,71 | 1187 | 0.1 ± 0.3  (0-0.9) | 0.1 ± 0.1  (0-0.3) | 0.1 ± 0.2  (0-0.5) | - | - | - | - | - | - | - | - | - |
| m/z: 82,71,67,43,57 | 1188 | - | 0.1 ± 0.2  (0-0.5) | tr | 0.7 ± 1.2  (0-2.9) | - | - | - | - | 0.1 ± 0.1  (0-0.2) | 0.1 ± 0.2  (0-0.4) | 0.2 ± 0.3  (0-0.7) | 0.3 ± 0.4  (0-0.9) |
| m/z: 82,67,71,43,53 | 1198 | 0.3 ± 0.8  (0-2.1) | - | 0.2 ± 0.6  (0-1.5) | - | - | - | - | - | - | - | - | - |
| m/z: 43,109,81,67,152 | 1208 | - | - | - | - | - | - | - | - | 0.1 ± 0.2 (0-0.4) | - | tr | - |
| m/z: 43,109,81,152,67 | 1209 | 1.3 ± 1.5  (0-3.4) | 2.1 ± 1.3  (0-3.4) | 1 ± 1.1  (0-2.5) | 1.7 ± 1.2  (0-3.1) | - | - | - | - | - | - | - | - |
| m/z: 43,79,94,59,105 | 1231 | - | - | - | - | 0.3 ± 0.6  (0-1.2) | - | tr | - | - | - | - | - |
| m/z: 150,107,91,39,79 | 1233 | tr | - | - | 0.1 ± 0.2  (0-0.5) | - | - | - | - | - | - | - | - |
| m/z: 71,43,98,83,55 | 1240 | - | - | - | - | - | - | - | - | 0.2 ± 0.3  (0-0.6) | - | tr | 0.2 ± 0.3  (0-0.7) |
| m/z: 125,83,70,55,43 | 1246 | - | - | - | - | - | tr | - | tr | - | - | - | - |
| m/z: 43,85,71,99,57 | 1249 | - | - | - | - | tr | tr | tr | tr | - | - | - | - |
| m/z: 105,77,51,44,94 | 1250 | - | - | - | - | - | - | - | - | - | tr | - | tr |
| m/z: 95,67,41,82,39 | 1253 | tr | - | - | - | - | - | - | - | - | - | - | - |
| m/z: 67,43,71,82,55 | 1273 | - | 0.2 ± 0.4  (0-1.1) | tr | 0.4 ± 0.6  (0-1.4) | 0.3 ± 0.7  (0-1.4) | - | tr | - | 0.6 ± 0.6  (0-1.3) | 0.1 ± 0.1  (0-0.4) | 0.3 ± 0.4  (0-1) | 0.5 ± 0.6  (0-1.4) |
| m/z: 69,41,79,95,190 | 1279 | - | - | - | - | 0.3 ± 0.7  (0-1.4) | - | tr | - | - | - | - | - |
| m/z: 104,43,57,77,68 | 1311 | - | - | - | - | - | tr | - | 0.1 ± 0.2  (0-0.3) | - | - | - | - |
| m/z: 93,41,55,119,70 | 1366 | - | - | - | - | - | - | - | - | tr | tr | tr | tr |
| m/z: 161,189,84,71,55 | 1386 | - | - | - | - | - | tr | - | tr | - | - | - | - |
| m/z: 57,43,71,150,91 | 1399 | 0.1 ± 0.4  (0-1) | tr | 0.3 ± 0.5  (0-1.2) | - | - | - | - | - | - | - | - | - |
| m/z: 150,107,91,135,79 | 1428 | 1 ± 0.7  (0-1.9) | 0.7 ± 0.7  (0-1.7) | 0.8 ± 0.9  (0-2.2) | 0.2 ± 0.4  (0-0.8) | - | - | - | - | - | - | - | - |
| m/z: 79,108,39,82,95 | 1434 | 0.1 ± 0.2  (0-0.5) | - | - | 0.2 ± 0.3  (0-0.8) | - | - | - | - | - | - | - | - |
| m/z: 119,93,41,69,107 | 1449 | - | - | - | - | - | - | - | - | 0.2 ± 0.1  (0-0.4) | 0.6 ± 0.5  (0-1.2) | 0.2 ± 0.3  (0-0.7) | 0.4 ± 0.3  (0-0.9) |
| m/z: 69,93,41,133,105 | 1454 | - | - | - | - | tr | 0.5 ± 0.5  (0-1) | - | 0.4 ± 0.7  (0-1.3) | - | - | - | - |
| m/z: 93,107,133,79,41 | 1474 | - | - | - | - | tr | tr | - | tr | - | - | - | - |
| m/z: 105,41,57,77,91 | 1484 | - | - | - | - | tr | - | tr | - | - | - | - | - |
| m/z: 43,105,91,55,132 | 1496 | tr | - | tr | - | - | - | - | - | - | - | - | - |
| m/z: 104,57,85,41,77 | 1499 | - | - | - | - | - | - | - | - | 0.1 ± 0.2  (0-0.5) | 1.4 ± 2.8  (0-5.7) | - | tr |
| m/z: 161,57,91,105,119 | 1541 | - | - | - | tr | - | - | - | - | - | - | - | - |
| m/z: 55,41,93,67,79 | 1567 | - | - | - | - | 0.2 ± 0.2  (0-0.6) | tr | 0.4 ± 0.8  (0-1.8) | tr | - | - | - | - |
| m/z: 69,162,41,81,131 | 1586 | - | - | - | - | - | tr | - | tr | - | - | - | - |
| m/z: 43,109,151,213,57 | 1649 | - | - | - | - | tr | tr | tr | tr | - | - | - | - |
| m/z: 136,41,69,91,55 | 1664 | - | - | - | - | 0.7 ± 1.3  (0-2.7) | tr | tr | tr | - | - | - | - |
| m/z: 41,55,69,91,109 | 1667 | - | - | - | - | tr | - | tr | - | - | - | - | - |
|  |  |  |  |  |  |  |  |  |  |  |  |  |  |
| **Butterfly-pollinated plant species** | **RI** | ***L*. *camara +*** | | | | ***L*. *alba +*** | | | |  | | | |
|  |  | **Control** | | **Before florivory** | **After florivory** | **Control** | | **Before florivory** | **After florivory** |  |  |  |  |
|  |  | **SM1** | **SM2** | **SM1** | **SM2** | **SM1** | **SM2** | **SM1** | **SM2** |  |  |  |  |
| Total number of compounds |  | 4 | 8 | 6 | 7 | 44 | 41 | 44 | 41 |  |  |  |  |
| Nsamples (Nplants) |  | 2 (2) | 2 (2) | 2 (2) | 2 (2) | 3 (3) | 2 (2) | 3 (3) | 2 (2) |  |  |  |  |
| Total amount of scent emitted (ng/ flower/ h) |  | 160.4 ± 70.3 | 173.9 ± 89.5 | 286.0 ± 260.6 | 127.1 ± 64.2 | 21.0 ± 2.8 | 79.6 ± 85.1 | 14.5 ± 1.1 | 85.1 ± 102.6 |  |  |  |  |
|  |  |  |  |  |  |  |  |  |  |  |  |  |  |
| **Aliphatic compounds** |  |  |  |  |  |  |  |  |  |  |  |  |  |
| 4-Methyl-3-pentene-2-ol* | 793 | - | - | - | - | tr | 0.1 ± 0.2  (0-0.2) | 0.1 ± 0.2  (0-0.3) | 0.8 ± 1.2  (0-1.7) |  |  |  |  |
| Hexanal* | 796 | - | - | - | - | - | 0.1 ± 0.2  (0-0.3) | - | 0.1 ± 0.1  (0-0.2) |  |  |  |  |
| 3-Octanol* | 993 | - | - | - | - | 0.1 ± 0.1  (0.05-0.2) | 0.2 ± 0.007  (0.22-0.23) | 0.1 ± 0.07  (0.07-0.2) | 0.3 ± 0.2  (0.1-0.5) |  |  |  |  |
| Methyl octanoate* | 1120 | 43 ± 20.1  (28.7-57.2) | 1.3 ± 1.8  (0-2.6) | 33.2 ± 46.9  (0-66.4) | - | - | - | - | - |  |  |  |  |
| (*Z*)-3-Hexenyl butyrate* | 1188 | - | - | - | - | 0.1 ± 0.2  (0.03-0.4) | tr | 0.1 ± 0.1  (0.04-0.3) | 0.3 ± 0.5  (0-0.7) |  |  |  |  |
| Methyl decanoate | 1322 | - | 1.8 ± 2.5  (0-3.6) | - | 2.8 ± 4.0  (0-5.7) | - | - | - | - |  |  |  |  |
| **Aromatic compounds** |  |  |  |  |  |  |  |  |  |  |  |  |  |
| Benzaldehyde* | 963 | - | 5.4 ± 7.6  (0-10.8) | 15.4 ± 21.9  (0-30.9) | 3.3 ± 4.7  (0-6.7) | 0.3 ± 0.1  (0.1-0.4) | 0.4 ± 0.1  (0.3-0.5) | 0.2 ± 0.2  (0.06-0.4) | 0.7 ± 0.6  (0.3-1.2) |  |  |  |  |
| Methyl salicylate* | 1204 | - | 7.6 ± 10.8  (0-15.2) | - | 11.8 ± 16.8  (0-23.7) | 1 ± 1.3  (0.2-2.6) | 0.8 ± 0.6  (0.3-1.3) | 0.7 ± 0.8  (0.1-1.6) | 0.9 ± 0.1  (0.8-1) |  |  |  |  |
| **Terpenoids** |  |  |  |  |  |  |  |  |  |  |  |  |  |
| a 2-Methyl-2-vinyl-5-hydroxytetrahydrofuran | 928 | - | - | - | - | 0.5 ± 0.3  (0.2-0.9) | 1.2 ± 0.6  (0.7-1.6) | 0.7 ± 0.7  (0.07-1.4) | 1.8 ± 1.4  (0.8-2.8) |  |  |  |  |
| a 2-Methyl-2-vinyl-5-hydroxytetrahydrofuran | 946 | - | - | - | - | 0.4 ± 0.3  (0.1-0.8) | 0.7 ± 0.6  (0.3-1.1) | 0.6 ± 0.6  (0.04-1.3) | 0.9 ± 0.5  (0.5-1.3) |  |  |  |  |
| 6-Methyl-5-hepten-2-one* | 987 | - | 5.2 ± 7.4  (0-10.4) | 16.1 ± 22.7  (0-32.2) | 3.7 ± 5.3  (0-7.5) | 2 ± 0.7  (1.2-2.7) | 3 ± 0.6  (2.5-3.4) | 1.7 ± 0.9  (0.6-2.3) | 2.3 ± 3.3  (0-4.6) |  |  |  |  |
| β-Myrcene* | 990 | - | - | - | - | 0.9 ± 0.2  (0.7-1.2) | 0.7 ± 1  (0-1.5) | 1 ± 0.1  (0.9-1.2) | 0.8 ± 1.1  (0-1.6) |  |  |  |  |
| Limonene* | 1036 | - | - | - | - | 0.2 ± 0.3  (0-0.6) | 0.2 ± 0.4  (0-0.5) | 0.1 ± 0.1  (0-0.3) | 0.4 ± 0.5  (0-0.8) |  |  |  |  |
| Lavender lactone* | 1044 | - | - | - | - | 0.6 ± 0.2  (0.4-1) | 1.4 ± 0.6  (0.9-1.9) | 0.8 ± 0.7  (0.06-1.5) | 1.4 ± 0.5  (1-1.8) |  |  |  |  |
| (*E*)-β-Ocimene* | 1049 | - | 20.4 ± 14.6  (10.0-30.7) | 11.7 ± 16.5  (0-23.4) | 5.0 ± 7.1  (0-10.1) | 0.4 ± 0.1  (0.3-0.6) | 0.7 ± 0.1  (0.6-0.8) | 0.9 ± 0.5  (0.4-1.4) | 0.7 ± 0.1  (0.5-0.8) |  |  |  |  |
| (*E*)-Linalool oxide furanoid* | 1092 | - | - | - | - | 0.5 ± 0.1  (0.3-0.6) | 0.5 ± 0.06  (0.5-0.6) | 0.5 ± 0.1  (0.3-0.7) | 0.7 ± 0.1  (0.6-0.8) |  |  |  |  |
| Linalool* | 1099 | 33.5 ± 47.5  (0-67.1) | 54.9 ± 19.3  (41.2-68.5) | 6.6 ± 9.4  (0-13.3) | 66.9 ± 4.0  (64.1-69.8) | 58.5 ± 27.7  (26.6-76.7) | 75.6 ± 1.5  (74.6-76.7) | 68.9 ± 2.5  (66.6-71.6) | 69.0 ± 5.9  (64.8-73.3) |  |  |  |  |
| Hotrienol | 1104 | - | - | - | - | 3.4 ± 0.8  (2.4-4) | 2.0 ± 2.9  (0-4.1) | 2.5 ± 2.4  (0-4.8) | 6.0 ± 0.7  (5.5-6.5) |  |  |  |  |
| (*E*)-4,8-dimethyl-1,3,7-nonatriene* | 1119 | 2.0 ± 2.8  (0-4.0) | 3.1 ± 4.5  (0-6.3) | - | 6.0 ± 8.5  (0-12.0) | 1.1 ± 0.6  (0.4-1.6) | 0.9 ± 0.07  (0.9-1.0) | 0.9 ± 0.3  (0.5-1.2) | 1.7 ± 0.3  (1.4-2.0) |  |  |  |  |
| Ocimene derivative | 1125 | - | - | - | - | 0.3 ± 0.08  (0.2-0.4) | 0.3 ± 0.1  (0.2-0.4) | 0.3 ± 0.07  (0.2-0.4) | 0.4 ± 0.1  (0.3-0.5) |  |  |  |  |
| *allo*-Ocimene* | 1130 | - | - | - | - | - | - | - | - |  |  |  |  |
| *p*-1,3,8-Menthatriene | 1134 | - | - | - | - | 0.4 ± 0.7  (0-1.3) | 0.4 ± 0.6  (0-0.9) | 0.8 ± 0.7  (0-1.5) | 0.5 ± 0.8  (0-1.1) |  |  |  |  |
| (*E*)-linalool oxide pyranoid* | 1175 | - | - | - | - | 0.1 ± 0.04  (0.06-0.1) | tr | 0.1 ± 0.03  (0.08-0.1) | tr |  |  |  |  |
| α-Terpineol* | 1198 | - | - | - | - | 0.4 ± 0.1  (0.2-0.5) | 0.7 ± 0.1  (0.6-0.9) | 0.5 ± 0.1  (0.4-0.7) | 0.9 ± 0.6  (0.4-1.3) |  |  |  |  |
| Neral* | 1245 | - | - | - | - | tr | tr | 0.1 ± 0.08  (0-0.2) | 0.1 ± 0.1  (0-0.2) |  |  |  |  |
| Geranial* | 1273 | - | - | - | - | 0.7 ± 0.2  (0.5-1) | 0.3 ± 0.4  (0-0.6) | 0.7 ± 0.3  (0.3-1) | 0.3 ± 0.4  (0-0.6) |  |  |  |  |
| β-Bourbonene* | 1407 | - | - | - | - | 3.1 ± 2.6  (1.5-6.2) | 1 ± 0.1  (0.9-1.1) | 2.5 ± 1.5  (0.7-3.6) | 0.8 ± 1.1  (0.08-1.6) |  |  |  |  |
| β-Caryophyllene* | 1444 | - | - | - | - | 2.9 ± 2.7  (0.6-6.0) | 0.8 ± 0.4 (0.4-1.1) | 3.3 ± 2.4 (0.6-5.2) | 0.2 ± 0.2 (0.06-0.4) |  |  |  |  |
| (*E*)-β-Farnesene* | 1461 | - | - | - | - | 4.0 ± 4.6  (1.0-9.3) | - | 1.3 ± 1.4  (0-2.8) | - |  |  |  |  |
| Germacrene D* | 1497 | - | - | - | - | 0.3 ± 0.1  (0.2-0.4) | tr | 0.8 ± 0.4  (0.5-1.3) | 0.3 ± 0.5  (0-0.7) |  |  |  |  |
| α-Amorphene | 1520 | - | - | - | - | 0.6 ± 0.8  (0.001-1.6) | 0.2 ± 0.3  (0-0.5) | 0.5 ± 0.07  (0.4-0.6) | 0.1 ± 0.2  (0-0.3) |  |  |  |  |
| Kessane | 1705 | - | - | - | - | 3.3 ± 3.7  (1.1-7.7) | 2.1 ± 1.6  (0.9-3.3) | 1.7 ± 1.6  (0-3.1) | 1.5 ± 2.1  (0-3.0) |  |  |  |  |
| **Unidentified compounds** |  |  |  |  |  |  |  |  |  |  |  |  |  |
| m/z: 133,151,43,95,67 | 888 | - | - | - | - | tr | - | tr | - |  |  |  |  |
| m/z: 81,41,69,79,150 | 1015 | - | - | - | - | 0.3 ± 0.2  (0.1-0.6) | 0.4 ± 0.1  (0.2-0.5) | 0.3 ± 0.1  (0.1-0.4) | 0.3 ± 0.4  (0-0.6) |  |  |  |  |
| m/z: 91,119,134,41,77 | 1082 | - | - | - | - | 0.1 ± 0.09  (0.04-0.2) | 0.1 ± 0.02  (0.1-0.2) | 0.2 ± 0.1  (0.1-0.3) | 0.1 ± 0.02  (0.1-0.1) |  |  |  |  |
| m/z: 59,94,79,43,105 | 1174 | - | - | - | - | 0.4 ± 0.3  (0.1-0.8) | 0.4 ± 0.6  (0-0.9) | 1.0 ± 0.3  (0.7-1.3) | 0.2 ± 0.3  (0-0.4) |  |  |  |  |
| m/z: 43,135,107,91,65 | 1203 | 21.3 ± 30.2  (0-42.7) | - | 16.7 ± 23.7  (0-33.5) | - | - | - | - | - |  |  |  |  |
| m/z: 43,109,81,67,152 | 1208 | - | - | - | - | 0.4 ± 0.2  (0.1-0.6) | 0.4 ± 0.1  (0.4-0.5) | 0.6 ± 0.2  (0.3-0.9) | 1.3 ± 0.8  (0.7-1.9) |  |  |  |  |
| m/z: 43,94,68,55,79 | 1290 | - | - | - | - | tr | tr | tr | 0.1 ± 0.1  (0-0.2) |  |  |  |  |
| m/z: 82,73,43,67,71 | 1322 | - | - | - | - | tr | - | tr | - |  |  |  |  |
| m/z: 82,43,71,67,55 | 1332 | - | - | - | - | 1.6 ± 2.1  (0.3-4.1) | 0.4 ± 0.6  (0-0.9) | 0.5 ± 0.5  (0.06-1.2) | 0.1 ± 0.2  (0-0.3) |  |  |  |  |
| m/z: 93,55,41,85,77 | 1381 | - | - | - | - | 1.3 ± 1.8  (0.2-3.5) | 0.2 ± 0.03  (0.2-0.3) | 0.3 ± 0.1  (0.2-0.5) | 0.4 ± 0.2  (0.2-0.6) |  |  |  |  |
| m/z: 161,120,105,91,43 | 1441 | - | - | - | - | 0.4 ± 0.3  (0.2-0.7) | 0.2 ± 0.04  (0.1-0.2) | 0.5 ± 0.4  (0.2-1.0) | 0.2 ± 0  (0.2-0.2) |  |  |  |  |
| m/z: 93,121,105,41,55 | 1448 | - | - | - | - | 0.1 ± 0.3  (0-0.5) | tr | 0.2 ± 0.3  (0-0.6) | tr |  |  |  |  |
| m/z: 119,93,41,69,107 | 1449 | - | - | - | - | 0.3 ± 0.3  (0-0.7) | tr | 0.2 ± 0.2  (0-0.5) | tr |  |  |  |  |
| m/z: 161,57,91,105,119 | 1541 | - | - | - | - | 0.1 ± 0.1  (0-0.2) | - | 0.3 ± 0.2  (0-0.5) | - |  |  |  |  |
| m/z: 43,91,121,79,107 | 1739 | - | - | - | - | 4.7 ± 7.4  (0-13.3) | 1.4 ± 1.1  (0.5-2.2) | 1.1 ± 0.9  (0-1.7) | 1.1 ± 1.6  (0-2.3) |  |  |  |  |
| m/z: 43,93,55,107,85 | 1767 | - | - | - | - | 0.5 ± 0.9  (0-1.6) | tr | tr | tr |  |  |  |  |
| m/z: 43,95,55,81,159 | 1779 | - | - | - | - | 1.3 ± 1.9  (0.1-3.5) | 0.2 ± 0.1  (0.1-0.3) | 0.2 ± 0.1  (0.1-0.4) | 0.2 ± 0.2  (0.1-0.4) |  |  |  |  |
|  |  |  |  |  |  |  |  |  |  |  |  |  |  |
| **Hawkmoth-pollinated plant species** | **RI** | ***T*. *formosa*** | | | |  |  |  |  |  |  |  |  |
|  |  | **Control** | | **Before florivory** | **After florivory** |  |  |  |  |  |  |  |  |
|  |  | **SM1** | **SM2** | **SM1** | **SM2** |  |  |  |  |  |  |  |  |
| Total number of compounds |  | 27 | 29 | 27 | 29 |  |  |  |  |  |  |  |  |
| Nsamples (Nplants) |  | 4 (4) | 4 (4) | 4 (4) | 4 (4) |  |  |  |  |  |  |  |  |
| Total amount of scent emitted (ng/ flower/ h) |  | 646.8 ± 148.4 | 437.9 ± 108.5 | 664.4 ± 81.3 | 240.8 ± 110.5 |  |  |  |  |  |  |  |  |
|  |  |  |  |  |  |  |  |  |  |  |  |  |  |
| **Aliphatic compounds** |  |  |  |  |  |  |  |  |  |  |  |  |  |
| Hexanal* | 796 | - | tr | - | tr |  |  |  |  |  |  |  |  |
| 1-Hexanol* | 864 | tr | tr | tr | tr |  |  |  |  |  |  |  |  |
| 1-Octen-3-ol* | 976 | tr | tr | tr | tr |  |  |  |  |  |  |  |  |
| **Aromatic compounds** |  |  |  |  |  |  |  |  |  |  |  |  |  |
| Benzaldehyde* | 963 | 0.2 ± 0.1  (0-0.4) | 0.2 ± 0.1  (0.06-0.3) | 0.2 ± 0.1  (0-0.4) | 0.2 ± 0.1  (0.05-0.5) |  |  |  |  |  |  |  |  |
| Benzyl alcohol* | 1036 | 14.0 ± 9.0  (0.5-18.7) | 9.8 ± 5.5  (2.1-15.3) | 13.1 ± 8.8  (0.3-20.0) | 8.6 ± 7.0  (1.1-18.1) |  |  |  |  |  |  |  |  |
| Methyl benzoate* | 1100 | 43.5 ± 6  (37.9-51.9) | 10.2 ± 3.1  (6.5-13.8) | 45.9 ± 4.5  (42.0-52.4) | 15.1 ± 2.7  (11-16.8) |  |  |  |  |  |  |  |  |
| 2-Phenylethanol* | 1117 | tr | 0.1 ± 0.04  (0.08-0.1) | tr | 0.1 ± 0.05  (0.06-0.1) |  |  |  |  |  |  |  |  |
| *p*-Vinylanisole* | 1159 | - | tr | - | 0.1 ± 0.1  (0-0.2) |  |  |  |  |  |  |  |  |
| Benzyl acetate* | 1167 | tr | tr | tr | tr |  |  |  |  |  |  |  |  |
| Methyl salicylate* | 1204 | 0.5 ± 1.0  (0.003-2.1) | 0.8 ± 1.6  (0-3.4) | 0.6 ± 1.3  (0.02-2.6) | 0.4 ± 0.7  (0-1.6) |  |  |  |  |  |  |  |  |
| *p*-Anisaldehyde* | 1263 | - | tr | - | tr |  |  |  |  |  |  |  |  |
| Benzyl benzoate* | 1785 | 0.1 ± 0.2  (0-0.6) | tr | 0.1 ± 0.2  (0-0.4) | tr |  |  |  |  |  |  |  |  |
| **N-bearing compounds** |  |  |  |  |  |  |  |  |  |  |  |  |  |
| 2-Methylbutanenitrile | 716 | 0.1 ± 0.2  (0-0.4) | 0.2 ± 0.1  (0-0.4) | tr | 0.3 ± 0.3  (0-0.9) |  |  |  |  |  |  |  |  |
| Isobutylaldoxime | 747 | - | tr | - | tr |  |  |  |  |  |  |  |  |
| *syn*-3-Methylbutylaldoxime* | 848 | 0.3 ± 0.1  (0.2-0.6) | 0.3 ± 0.04  (0.2-0.3) | 0.3 ± 0.06  (0.2-0.4) | 0.4 ± 0.1  (0.3-0.5) |  |  |  |  |  |  |  |  |
| *syn*-2-Methylbutylaldoxime | 851 | 1.9 ± 2.3  (0.7-5.4) | 1.7 ± 1.2  (1-3.5) | 1.7 ± 1.9  (0.5-4.6) | 3.0 ± 3.0  (1.4-7.6) |  |  |  |  |  |  |  |  |
| *anti*-2-Methylbutylaldoxime | 858 | 0.8 ± 0.9  (0.2-2.3) | 0.7 ± 0.3  (0.5-1.2) | 0.8 ± 0.8  (0.1-2) | 1.2 ± 1.0  (0.6-2.7) |  |  |  |  |  |  |  |  |
| Methyl nicotinate* | 1143 | 0.2 ± 0.08  (0.1-0.3) | 0.8 ± 0.2  (0.6-1.1) | 0.2 ± 0.06  (0.1-0.3) | 0.6 ± 0.2  (0.4-0.9) |  |  |  |  |  |  |  |  |
| **Terpenoids** |  |  |  |  |  |  |  |  |  |  |  |  |  |
| 6-Methyl-5-hepten-2-one* | 987 | tr | 0.1 ± 0.03  (0.06-0.1) | tr | 0.2 ± 0.07  (0.1-0.3) |  |  |  |  |  |  |  |  |
| β-Myrcene* | 990 | tr | - | tr | - |  |  |  |  |  |  |  |  |
| (*Z*)-β-Ocimene* | 1037 | tr | - | tr | - |  |  |  |  |  |  |  |  |
| (*E*)-β-Ocimene* | 1049 | 0.1 ± 0.08  (0-0.2) | tr | 0.1 ± 0.1  (0-0.2) | 0.1 ± 0.1  (0.001-0.3) |  |  |  |  |  |  |  |  |
| (*Z*)-Linalool oxide furanoid* | 1076 | 2.9 ± 0.5  (2.3-3.6) | 2.1 ± 0.7  (1.2-3.0) | 2.9 ± 0.1  (2.6-3.1) | 2.7 ± 1.1  (1.6-4.3) |  |  |  |  |  |  |  |  |
| (*E*)-Linalool oxide furanoid* | 1092 | 0.6 ± 0.1  (0.5-0.8) | 0.5 ± 0.1  (0.3-0.6) | 0.6 ± 0.04  (0.5-0.6) | 0.5 ± 0.2  (0.3-0.8) |  |  |  |  |  |  |  |  |
| 2,2,6-Trimethy-6-vinyldihydro-2H-pyran-3(4H)-one* | 1112 | 0.8 ± 0.08  (0.7-0.9) | 0.4 ± 0.1  (0.2-0.6) | 0.9 ± 0.07  (0.8-0.9) | 0.4 ± 0.2  (0.2-0.7) |  |  |  |  |  |  |  |  |
| *allo*-Ocimene* | 1130 | tr | tr | tr | tr |  |  |  |  |  |  |  |  |
| (*Z*)-linalool oxide pyranoid* | 1175 | 2.9 ± 1.3  (1.3-4.1) | 1.2 ± 1.3  (0.09-3.1) | 3.5 ± 1.3  (1.6-4.9) | 0.9 ± 0.5  (0.3-1.3) |  |  |  |  |  |  |  |  |
| (*E*)-linalool oxide pyranoid* | 1175 | 29.8 ± 2.5  (26.7-32.8) | 69.8 ± 4.6  (65.3-74.2) | 28.1 ± 2.4  (25.5-30.3) | 63.9 ± 7.8  (52.4-69.7) |  |  |  |  |  |  |  |  |
| (3*E*,7*E*)-4,8,12-Trimethyltrideca-1,3,7,11-tetraene | 1584 | tr | tr | tr | tr |  |  |  |  |  |  |  |  |
| **Unidentified compounds** |  |  |  |  |  |  |  |  |  |  |  |  |  |
| m/z: 81,43,109,71,55 | 946 | tr | - | tr | - |  |  |  |  |  |  |  |  |
| m/z: 68,43,94,59,85 | 1237 | tr | tr | tr | tr |  |  |  |  |  |  |  |  |
| m/z: 43,94,68,55,79 | 1290 | - | tr | - | tr |  |  |  |  |  |  |  |  |
|  |  |  |  |  |  |  |  |  |  |  |  |  |  |
| **Hummingbird-pollinated plant species** | **RI** | ***Z*. *montana*** | | | |  |  |  |  |  |  |  |  |
|  |  | **Control** | | **Before florivory** | **After florivory** |  |  |  |  |  |  |  |  |
|  |  | **SM1** | **SM2** | **SM1** | **SM2** |  |  |  |  |  |  |  |  |
| Total number of compounds |  | 16 | 17 | 20 | 13 |  |  |  |  |  |  |  |  |
| Nsamples (Nplants) |  | 5 (5) | 5 (5) | 5 (5) | 6 (6) |  |  |  |  |  |  |  |  |
| Total amount of scent emitted (ng/ flower/ h) |  | 36,1 ± 24,1 | 72,3 ± 131,8 | 53,7 ± 105,5 | 68,8 ± 125,6 |  |  |  |  |  |  |  |  |
|  |  |  |  |  |  |  |  |  |  |  |  |  |  |
| **Aliphatic compounds** |  |  |  |  |  |  |  |  |  |  |  |  |  |
| Ethyl 2-methylacrylate | 783 | 3.0 ± 6.6  (0-14.8) | - | 1.5 ± 3.4  (0-7.5) | 27.9 ± 44.5  (0-100) |  |  |  |  |  |  |  |  |
| 1-Hexanol* | 864 | 0.2 ± 0.3  (0-1.5) | 3.3 ± 7.4  (0-16.6) | 0.2 ± 0.6  (0-1.3) | - |  |  |  |  |  |  |  |  |
| 2-Methylbutyl acetate* | 874 | 0.2 ± 0.3  (0-0.8) | 1.0 ± 1.6  (0-3.6) | 0.3 ± 0.6  (0-1.4) | - |  |  |  |  |  |  |  |  |
| 1-Octen-3-ol* | 976 | 18.1 ± 24.7  (0-59.9) | 6.6 ± 13.9  (0-31.4) | 0.3 ± 0.6  (0-1.4) | - |  |  |  |  |  |  |  |  |
| 3-Octanone* | 987 | - | - | 0.7 ± 1.6  (0-3.6) | 0.3 ± 0.7  (0-1.7) |  |  |  |  |  |  |  |  |
| (*Z*)-3-Hexenyl acetate | 1005 | 5.4 ± 8.9  (0-20.5) | 5.8 ± 13.0  (0-29) | - | - |  |  |  |  |  |  |  |  |
| 2-Ethylhexoic acid | 1111 | - | - | 12.7 ± 28.4  (0-63.5) | 9.0. ± 22.0  (0-53.8) |  |  |  |  |  |  |  |  |
| Tridecane* | 1300 | 3.7 ± 5.6  (0-12.7) | 16.2 ± 20.2  (0-39.6) | - | - |  |  |  |  |  |  |  |  |
| Tetradecane* | 1400 | 2.7 ± 6.0  (0-13.3) | 6.8 ± 12.5  (0-28.8) | 0.7 ± 1.5  (0-3.3) | 0.9 ± 2.1  (0-5.2) |  |  |  |  |  |  |  |  |
| **Aromatic compounds** |  |  |  |  |  |  |  |  |  |  |  |  |  |
| Benzaldehyde* | 963 | 30.5 ± 28.9  (0-63.6) | 3.6 ± 8.1  (0-18.2) | 9.1 ± 20.3  (0-45.3) | - |  |  |  |  |  |  |  |  |
| Benzyl alcohol* | 1036 | 23 ± 27.4  (0-69.7) | 31.7 ± 30.8  (0-79.9) | 14.2 ± 19.9  (0-41.6) | 13.0 ± 23.2  (0-57.1) |  |  |  |  |  |  |  |  |
| Methyl benzoate* | 1100 | - | - | 15.0 ± 33.6  (0-75.1) | 2.0 ± 4.9  (0-12.0) |  |  |  |  |  |  |  |  |
| Benzyl acetate* | 1167 | - | 0.2 ± 0.3  (0-0.8) | 20.0 ± 44.7  (0-99.9) | tr |  |  |  |  |  |  |  |  |
| *p*-Acetylacetophenone | 1464 | - | - | 6.6 ± 14.  (0-33.1) | 6.8 ± 16.8  (0-41.0) |  |  |  |  |  |  |  |  |
| **Terpenoids** |  |  |  |  |  |  |  |  |  |  |  |  |  |
| Lavender lactone* | 1044 | 0.7 ± 1.7  (0-3.7) | 0.7 ± 1.6  (0-3.6) | 1.0 ± 2.2  (0-4.8) | - |  |  |  |  |  |  |  |  |
| (*Z*)-Linalool oxide furanoid* | 1076 | 3.9 ± 8.8  (0-19.6) | 3.7 ± 6.8  (0-15.7) | 0.8 ± 1.8  (0-4.0) | 4.7 ± 11.6  (0-28.4) |  |  |  |  |  |  |  |  |
| (*E*)-Linalool oxide furanoid* | 1092 | - | 0.5 ± 1.2  (0-2.6) | 0.4 ± 1.0  (0-2.2) | - |  |  |  |  |  |  |  |  |
| Linalool* | 1099 | - | - | 4.9 ± 11.1  (0-24.9) | 10.3 ± 17.2  (0-41.2) |  |  |  |  |  |  |  |  |
| (*E*)-4,8-Dimethyl-1,3,7-nonatriene* | 1119 | - | - | 0.02 ± 0.1  (0-0.1) | 16.6 ± 40.7  (0-99.9) |  |  |  |  |  |  |  |  |
| Linalyl acetate | 1256 | - | 2.3 ± 5.2  (0-11.6) | 0.6 ± 1.3  (0-2.9) | - |  |  |  |  |  |  |  |  |
| Geranyl acetate* | 1384 | - | 1.6 ± 2.4  (0-5.3) | - | - |  |  |  |  |  |  |  |  |
| α-Copaene* | 1395 | 0.6 ± 1.4  (0-3.2) | 2.2 ± 3.8  (0-8.8) | - | - |  |  |  |  |  |  |  |  |
| (*E*)-β-Caryophyllene* | 1444 | - | - | 9.6 ± 21.5  (0-48.1) | 0.9 ± 2.3  (0-5.7) |  |  |  |  |  |  |  |  |
| 1-nor-Bourbonanone | 1588 | 0.4 ± 0.9  (0-2.1) | 1.8 ± 4.1  (0-9.2) | - | - |  |  |  |  |  |  |  |  |
| **Unidentified compounds** |  |  |  |  |  |  |  |  |  |  |  |  |  |
| m/z: 43,70,57,84,96 | 902 | 5.3 ± 8.5  (0-19.6) | 1.9 ± 4.1  (0-9.3) | - | - |  |  |  |  |  |  |  |  |
| m/z: 57,71,43,85,127 | 1056 | 2.1 ± 4.8  (0-10.7) | 10.1 ± 20.3  (0-46.2) | 1.3 ± 2.8  (0-6.3) | 7.5 ± 18.4  (0-45.1) |  |  |  |  |  |  |  |  |

**Table S4.** Detailed outcomes of PERMANOVA analyses that evaluated if florivory affected the total amount of scent and the relative composition of floral scent compounds in the study plant species. Random factors in the PERMANOVA design are marked in italics. Statistically significant results are marked in bold.

| *Amphilophium mansoanum* |  |  |  |  |
| --- | --- | --- | --- | --- |
| **Total amount** | Df | Pseudo-F | p | Unique permutations |
| Treatment | 1 | 0.13283 | 0.7224 | 9837 |
| Sampling moment | 1 | 3.8327 | 0.1234 | 9787 |
| *Individual* | 6 | 3.1394 | 0.1889 | 9960 |
| Treatment*Sampling moment | 1 | 0.2387 | 0.6553 | 9784 |
| Treatment**Individual* | 5 | 1.2404 | 0.4562 | 9974 |
| Sampling moment**Individual* | 4 | 2.0869 | 0.2834 | 9968 |
| Residuals | 3 |  |  |  |
| Total | 21 |  |  |  |
|  |  |  |  |  |
| **Relative amount** | Df | Pseudo-F | p | Unique permutations |
| Treatment | 1 | 1.0993 | 0.3954 | 9966 |
| Sampling moment | 1 | 3.133 | 0.0872 | 9923 |
| *Individual* | 6 | 1.3333 | 0.3101 | 9942 |
| Treatment*Sampling moment | 1 | 1.0571 | 0.4006 | 9909 |
| Treatment**Individual* | 5 | 0.6978 | 0.7172 | 9956 |
| Sampling moment**Individual* | 4 | 1.4207 | 0.3606 | 9963 |
| Residuals | 3 |  |  |  |
| Total | 21 |  |  |  |
| *Byrsonima intermedia* |  |  |  |  |
| **Total amount** | Df | Pseudo-F | p | Unique permutations |
| Treatment | 1 | 0.97027 | 0.3844 | 1394 |
| Sampling moment | 1 | 1.5772 | 0.3909 | 211 |
| *Individual* | 3 | 16.452 | 0.0606 | 9979 |
| Treatment*Sampling moment | 1 | 1.8527 | 0.3137 | 8028 |
| Treatment**Individual* | 3 | 1.1127 | 0.5043 | 9970 |
| Sampling moment**Individual* | 2 | 1.083 | 0.4786 | 9970 |
| Residuals | 2 |  |  |  |
| Total | 13 |  |  |  |
|  |  |  |  |  |
| **Relative amount** | Df | Pseudo-F | p | Unique permutations |
| Treatment | 1 | 1.8342 | 0.1856 | 1383 |
| Sampling moment | 1 | 1.1165 | 0.3763 | 212 |
| *Individual* | 3 | 5.3207 | 0.1050 | 9957 |
| Treatment*Sampling moment | 1 | 0.93873 | 0.4976 | 7797 |
| Treatment**Individual* | 3 | 1.0381 | 0.5123 | 9966 |
| Sampling moment**Individual* | 2 | 0.88312 | 0.5477 | 9962 |
| Residuals | 2 |  |  |  |
| Total | 13 |  |  |  |
| *Centrosema pubescens* |  |  |  |  |
| **Total amount** | Df | Pseudo-F | p | Unique permutations |
| Treatment | 1 | 8.42E-02 | 0.7772 | 417 |
| Sampling moment | 1 | 1.98E-02 | 0.9161 | 425 |
| *Individual* | 3 | 4.3655 | 0.1337 | 9975 |
| Treatment*Sampling moment | 1 | 0.61264 | 0.4909 | 9095 |
| Treatment**Individual* | 3 | 0.29292 | 0.8249 | 9972 |
| Sampling moment**Individual* | 3 | 6.1997 | 0.0838 | 9978 |
| Residuals | 3 |  |  |  |
| Total | 15 |  |  |  |
|  |  |  |  |  |
| **Relative amount** | Df | Pseudo-F | p | Unique permutations |
| Treatment | 1 | 0.71209 | 0.4639 | 425 |
| Sampling moment | 1 | 4.9876 | **0.0482** | 425 |
| *Individual* | 3 | 3.6875 | 0.0633 | 9958 |
| Treatment*Sampling moment | 1 | 0.15725 | 0.8405 | 9264 |
| Treatment**Individual* | 3 | 1.4989 | 0.3561 | 9966 |
| Sampling moment**Individual* | 3 | 1.8044 | 0.2432 | 9963 |
| Residuals | 3 |  |  |  |
| Total | 15 |  |  |  |
| *Tocoyena formosa* |  |  |  |  |
| **Total amount** | Df | Pseudo-F | p | Unique permutations |
| Treatment | 1 | 1.5659 | 0.3539 | 424 |
| Sampling moment | 1 | 25.976 | **0.0305** | 425 |
| *Individual* | 3 | 2.8956 | 0.2112 | 9955 |
| Treatment*Sampling moment | 1 | 10.8 | **0.0493** | 9222 |
| Treatment**Individual* | 3 | 4.8224 | 0.1187 | 9972 |
| Sampling moment**Individual* | 3 | 3.6089 | 0.1648 | 9975 |
| Residuals | 3 |  |  |  |
| Total | 15 |  |  |  |
|  |  |  |  |  |
| **Relative amount** | Df | Pseudo-F | p | Unique permutations |
| Treatment | 1 | 2.169 | 0.2226 | 425 |
| Sampling moment | 1 | Negative |  |  |
| *Individual* | 3 | 17.692 | **0.0157** | 9958 |
| Treatment*Sampling moment | 1 | 2.0741 | 0.1849 | 9228 |
| Treatment**Individual* | 3 | 0.70417 | 0.6483 | 9957 |
| Sampling moment**Individual* | 3 | Negative |  |  |
| Residuals | 3 |  |  |  |
| Total | 15 |  |  |  |
| *Zeyheria montana* |  |  |  |  |
| **Total amount** | Df | Pseudo-F | p | Unique permutations |
| Treatment | 1 | 2.00E-02 | 0.8738 | 9114 |
| Sampling moment | 1 | 1.5734 | 0.3585 | 8998 |
| *Individual* | 5 | 2.5592 | 0.1933 | 9970 |
| Treatment*Sampling moment | 1 | 5.03E-02 | 0.8317 | 9843 |
| Treatment**Individual* | 4 | 4.4872 | 0.0846 | 9962 |
| Sampling moment**Individual* | 4 | 0.69067 | 0.6571 | 9967 |
| Residuals | 4 |  |  |  |
| Total | 20 |  |  |  |
|  |  |  |  |  |
| **Relative amount** | Df | Pseudo-F | p | Unique permutations |
| Treatment | 1 | 1.6818 | 0.1897 | 9002 |
| Sampling moment | 1 | 1.2888 | 0.304 | 8760 |
| *Individual* | 5 | 5.2627 | **0.0003** | 9942 |
| Treatment*Sampling moment | 1 | 1.5194 | 0.2521 | 9948 |
| Treatment**Individual* | 4 | 3.4938 | **0.0027** | 9916 |
| Sampling moment**Individual* | 4 | 1.8543 | 0.0862 | 9942 |
| Residuals | 4 |  |  |  |
| Total | 20 |  |  |  |

**Table S5.** Pairwise comparisons of the effect of ‘Treatment*Sampling moment’ for the total amount of scent of *Tocoyena formosa* flowers. Statistically significant results are marked in bold. SM: Sampling moment.

| ‘Treatment’ effect | | | | ‘Sampling moment’ effect | | | |
| --- | --- | --- | --- | --- | --- | --- | --- |
| Comparison | T value | p-value | Unique Perm | Comparison | T value | p-value | Unique Perm |
| SM1 Control-Before florivory | 0.1736 | 0.8601 | 425 | Control SM1-SM2 | 2.5949 | 0.0907 | 425 |
| SM2 Control-After florivory | 4.2366 | **0.0489** | 411 | Florivory SM1-SM2 | 7.3153 | **0.0284** | 425 |

**Table S6.** Detailed outcomes of PERMANOVA analyses for the individual scent compounds in each plant species.

| *Amphilophium mansoanum* |  |  |  |  |
| --- | --- | --- | --- | --- |
| **2-Butenal** | df | Pseudo-F | p | Unique perms |
| Treatment | 1 | 3.0615 | 0.1775 | 4093 |
| Sampling moment | 1 | 1.2963 | 0.4237 | 245 |
| *Individual* | 6 | 1.8435 | 0.332 | 9974 |
| Treatment*Sampling moment | 1 | 1 | 0.5277 | 11 |
| Treatment**Individual* | 5 | 1.9418 | 0.3095 | 9962 |
| Sampling moment**Individual* | 4 | 0.75 | 0.6405 | 2653 |
| Residuals | 3 |  |  |  |
| Total | 21 |  |  |  |
| **2-Butanyl acetate** | df | Pseudo-F | p | Unique perms |
| Treatment | 1 | 3.0615 | 0.1763 | 3971 |
| Sampling moment | 1 | 1.2963 | 0.4227 | 245 |
| *Individual* | 6 | 1.8435 | 0.3406 | 9970 |
| Treatment*Sampling moment | 1 | 1 | 0.531 | 11 |
| Treatment**Individual* | 5 | 1.9418 | 0.3201 | 9966 |
| Sampling moment**Individual* | 4 | 0.75 | 0.6491 | 2697 |
| Residuals | 3 |  |  |  |
| Total | 21 |  |  |  |
| **Hexanal** | df | Pseudo-F | p | Unique perms |
| Treatment | 1 | 2.0029 | 0.2328 | 9930 |
| Sampling moment | 1 | 2.3065 | 0.1956 | 9401 |
| *Individual* | 6 | 22.217 | **0.0219** | 9965 |
| Treatment*Sampling moment | 1 | 2.84E-04 | 0.9758 | 8497 |
| Treatment**Individual* | 5 | 13.932 | **0.0318** | 9960 |
| Sampling moment**Individual* | 4 | 2.2194 | 0.2712 | 9968 |
| Residuals | 3 |  |  |  |
| Total | 21 |  |  |  |
| **Methyl hexanoate** | df | Pseudo-F | p | Unique perms |
| Treatment | 1 | 0.68002 | 0.453 | 9785 |
| Sampling moment | 1 | 2.0697 | 0.2393 | 9376 |
| *Individual* | 6 | 2.2267 | 0.2786 | 9969 |
| Treatment*Sampling moment | 1 | 1.6472 | 0.2933 | 8403 |
| Treatment**Individual* | 5 | 1.5944 | 0.3663 | 9970 |
| Sampling moment**Individual* | 4 | 0.31333 | 0.8555 | 9969 |
| Residuals | 3 |  |  |  |
| Total | 21 |  |  |  |
| **α-Pinene** | df | Pseudo-F | p | Unique perms |
| Treatment | 1 | 1.5 | 0.3644 | 2285 |
| Sampling moment | 1 | 1.2963 | 0.4324 | 245 |
| *Individual* | 6 | 1.3344 | 0.4179 | 9961 |
| Treatment*Sampling moment | 1 | 1 | 0.5331 | 11 |
| Treatment**Individual* | 5 | 0.6 | 0.7199 | 3397 |
| Sampling moment**Individual* | 4 | 0.75 | 0.6388 | 2637 |
| Residuals | 3 |  |  |  |
| Total | 21 |  |  |  |
| **β-Pinene** | df | Pseudo-F | p | Unique perms |
| Treatment | 1 | 0.14286 | 0.6572 | 2304 |
| Sampling moment | 1 | 1.0082 | 0.4465 | 229 |
| *Individual* | 6 | 12.703 | **0.0401** | 9966 |
| Treatment*Sampling moment | 1 | 1 | 0.5374 | 11 |
| Treatment**Individual* | 5 | 0.73333 | 0.6504 | 7201 |
| Sampling moment**Individual* | 4 | 17.077 | **0.0209** | 9970 |
| Residuals | 3 |  |  |  |
| Total | 21 |  |  |  |
| **β-Myrcene** | df | Pseudo-F | p | Unique perms |
| Treatment | 1 | 0.76117 | 0.4194 | 9835 |
| Sampling moment | 1 | 8.57E-03 | 0.9239 | 9760 |
| *Individual* | 6 | 3.0076 | 0.1908 | 9972 |
| Treatment*Sampling moment | 1 | 5.3211 | 0.1027 | 9799 |
| Treatment**Individual* | 5 | 1.2806 | 0.4455 | 9960 |
| Sampling moment**Individual* | 4 | 3.7088 | 0.1522 | 9966 |
| Residuals | 3 |  |  |  |
| Total | 21 |  |  |  |
| **(*Z*)-β-Ocimene** | df | Pseudo-F | p | Unique perms |
| Treatment | 1 | 2.3359 | 0.1994 | 9851 |
| Sampling moment | 1 | 0.94007 | 0.3924 | 9813 |
| *Individual* | 6 | 0.83082 | 0.6085 | 9969 |
| Treatment*Sampling moment | 1 | 0.12593 | 0.7401 | 9822 |
| Treatment**Individual* | 5 | 0.36642 | 0.8402 | 9966 |
| Sampling moment**Individual* | 4 | 1.5707 | 0.3785 | 9967 |
| Residuals | 3 |  |  |  |
| Total | 21 |  |  |  |
| **(*E*)-β-Ocimene** | df | Pseudo-F | p | Unique perms |
| Treatment | 1 | 0.38482 | 0.5616 | 9848 |
| Sampling moment | 1 | 0.88213 | 0.3873 | 9794 |
| *Individual* | 6 | 0.22232 | 0.9466 | 9964 |
| Treatment*Sampling moment | 1 | 0.21077 | 0.6791 | 9788 |
| Treatment**Individual* | 5 | 0.28324 | 0.8969 | 9962 |
| Sampling moment**Individual* | 4 | 0.76059 | 0.6178 | 9968 |
| Residuals | 3 |  |  |  |
| Total | 21 |  |  |  |
| **(*Z*)-linalool oxide furanoid** | df | Pseudo-F | p | Unique perms |
| Treatment | 1 | 1.2665 | 0.3798 | 9244 |
| Sampling moment | 1 | 0.98385 | 0.4817 | 4305 |
| *Individual* | 6 | 0.64872 | 0.6808 | 9964 |
| Treatment*Sampling moment | 1 | 1.4178 | 0.355 | 1087 |
| Treatment**Individual* | 5 | 0.72835 | 0.6538 | 9962 |
| Sampling moment**Individual* | 4 | 0.90893 | 0.5353 | 9971 |
| Residuals | 3 |  |  |  |
| Total | 21 |  |  |  |
| **(*E*)-linalool oxide furanoid** | df | Pseudo-F | p | Unique perms |
| Treatment | 1 | 1.7004 | 0.322 | 9097 |
| Sampling moment | 1 | 2.9514 | 0.1896 | 4395 |
| *Individual* | 6 | 0.62113 | 0.7014 | 9969 |
| Treatment*Sampling moment | 1 | 1.0463 | 0.3489 | 1017 |
| Treatment**Individual* | 5 | 0.58368 | 0.7284 | 9973 |
| Sampling moment**Individual* | 4 | 0.7424 | 0.6272 | 9963 |
| Residuals | 3 |  |  |  |
| Total | 21 |  |  |  |
| **Linalool** | df | Pseudo-F | p | Unique perms |
| Treatment | 1 | 0.87462 | 0.4033 | 9867 |
| Sampling moment | 1 | 4.7582 | 0.084 | 9858 |
| *Individual* | 6 | 2.9963 | 0.2071 | 9976 |
| Treatment*Sampling moment | 1 | 0.60874 | 0.5004 | 9836 |
| Treatment**Individual* | 5 | 0.9879 | 0.5275 | 9974 |
| Sampling moment**Individual* | 4 | 4.4656 | 0.1246 | 9967 |
| Residuals | 3 |  |  |  |
| Total | 21 |  |  |  |
| **2-Phenylethanol** | df | Pseudo-F | p | Unique perms |
| Treatment | 1 | 3.8035 | 0.1124 | 9830 |
| Sampling moment | 1 | 7.7963 | 0.0581 | 9778 |
| *Individual* | 6 | 10.003 | **0.0423** | 9967 |
| Treatment*Sampling moment | 1 | 0.98686 | 0.3887 | 9706 |
| Treatment**Individual* | 5 | 0.55531 | 0.7333 | 9978 |
| Sampling moment**Individual* | 4 | 4.2296 | 0.1405 | 9958 |
| Residuals | 3 |  |  |  |
| Total | 21 |  |  |  |
| **Ocimene derivative** | df | Pseudo-F | p | Unique perms |
| Treatment | 1 | 1.5132 | 0.3088 | 9742 |
| Sampling moment | 1 | 0.10924 | 0.7482 | 9366 |
| *Individual* | 6 | 0.65582 | 0.6781 | 9964 |
| Treatment*Sampling moment | 1 | 1.7487 | 0.3043 | 8412 |
| Treatment**Individual* | 5 | 0.51531 | 0.7709 | 9979 |
| Sampling moment**Individual* | 4 | 0.9115 | 0.5513 | 9978 |
| Residuals | 3 |  |  |  |
| Total | 21 |  |  |  |
| ***allo*-Ocimene** | df | Pseudo-F | p | Unique perms |
| Treatment | 1 | 3.6077 | 0.1175 | 9830 |
| Sampling moment | 1 | 1.72E-02 | 0.9029 | 9807 |
| *Individual* | 6 | 1.9448 | 0.3103 | 9969 |
| Treatment*Sampling moment | 1 | 2.7896 | 0.1919 | 9286 |
| Treatment**Individual* | 5 | 0.14325 | 0.968 | 9967 |
| Sampling moment**Individual* | 4 | 2.8036 | 0.2138 | 9973 |
| Residuals | 3 |  |  |  |
| Total | 21 |  |  |  |
| ***p*-1.3.8-Menthatriene** | df | Pseudo-F | p | Unique perms |
| Treatment | 1 | 1.7723 | 0.2453 | 9853 |
| Sampling moment | 1 | 2.7354 | 0.1741 | 9808 |
| *Individual* | 6 | 2.1082 | 0.2917 | 9977 |
| Treatment*Sampling moment | 1 | 6.6091 | 0.0844 | 9851 |
| Treatment**Individual* | 5 | 2.3019 | 0.2665 | 9962 |
| Sampling moment**Individual* | 4 | 1.7847 | 0.3295 | 9965 |
| Residuals | 3 |  |  |  |
| Total | 21 |  |  |  |
| **(*E*)-Ocimene epoxide** | df | Pseudo-F | p | Unique perms |
| Treatment | 1 | 0.44768 | 0.5338 | 9840 |
| Sampling moment | 1 | 0.22997 | 0.6476 | 9819 |
| *Individual* | 6 | 1.0095 | 0.5547 | 9964 |
| Treatment*Sampling moment | 1 | 1.3902 | 0.3245 | 9720 |
| Treatment**Individual* | 5 | 0.87445 | 0.5791 | 9970 |
| Sampling moment**Individual* | 4 | 1.8976 | 0.3119 | 9969 |
| Residuals | 3 |  |  |  |
| Total | 21 |  |  |  |
| ***neoallo*-Ocimene** | df | Pseudo-F | p | Unique perms |
| Treatment | 1 | 2.15E-02 | 0.8751 | 9324 |
| Sampling moment | 1 | 1.8814 | 0.278 | 4364 |
| *Individual* | 6 | 1.2245 | 0.4649 | 9969 |
| Treatment*Sampling moment | 1 | 2.9733 | 0.183 | 1079 |
| Treatment**Individual* | 5 | 0.7265 | 0.6546 | 9962 |
| Sampling moment**Individual* | 4 | 1.2244 | 0.4572 | 9963 |
| Residuals | 3 |  |  |  |
| Total | 21 |  |  |  |
| **Ethyl octanoate** | df | Pseudo-F | p | Unique perms |
| Treatment | 1 | 1.6 | 0.3453 | 373 |
| Sampling moment | 1 | 1.75 | 0.3829 | 8 |
| *Individual* | 6 | Denominator is 0 |  |  |
| Treatment*Sampling moment | 1 | Denominator is 0 |  |  |
| Treatment**Individual* | 5 | Denominator is 0 |  |  |
| Sampling moment**Individual* | 4 | Denominator is 0 |  |  |
| Residuals | 3 |  |  |  |
| Total | 21 |  |  |  |
| **Indole** | df | Pseudo-F | p | Unique perms |
| Treatment | 1 | 1.4952 | 0.37 | 2845 |
| Sampling moment | 1 | 1.316 | 0.4138 | 253 |
| *Individual* | 6 | 0.74028 | 0.622 | 9966 |
| Treatment*Sampling moment | 1 | 1 | 0.5236 | 11 |
| Treatment**Individual* | 5 | 0.62291 | 0.7125 | 9968 |
| Sampling moment**Individual* | 4 | 0.62882 | 0.6917 | 9963 |
| Residuals | 3 |  |  |  |
| Total | 21 |  |  |  |
| **Eugenol** | df | Pseudo-F | p | Unique perms |
| Treatment | 1 | 0.4733 | 0.5301 | 9800 |
| Sampling moment | 1 | 1.7913 | 0.2622 | 9259 |
| *Individual* | 6 | 1.2899 | 0.4563 | 9958 |
| Treatment*Sampling moment | 1 | 0.80997 | 0.4537 | 8508 |
| Treatment**Individual* | 5 | 0.90045 | 0.5692 | 9963 |
| Sampling moment**Individual* | 4 | 1.646 | 0.3566 | 9958 |
| Residuals | 3 |  |  |  |
| Total | 21 |  |  |  |
| **β-Bourbonene** | df | Pseudo-F | p | Unique perms |
| Treatment | 1 | 1.34E-02 | 0.9753 | 2848 |
| Sampling moment | 1 | 1.3307 | 0.3615 | 253 |
| *Individual* | 6 | 0.58886 | 0.7178 | 9956 |
| Treatment*Sampling moment | 1 | 1 | 0.5283 | 11 |
| Treatment**Individual* | 5 | 0.16392 | 0.9589 | 9967 |
| Sampling moment**Individual* | 4 | 0.13158 | 0.9624 | 9971 |
| Residuals | 3 |  |  |  |
| Total | 21 |  |  |  |
| **β-Caryophyllene** | df | Pseudo-F | p | Unique perms |
| Treatment | 1 | 2.2821 | 0.2018 | 9773 |
| Sampling moment | 1 | 0.44566 | 0.5848 | 9350 |
| *Individual* | 6 | 0.95643 | 0.5509 | 9973 |
| Treatment*Sampling moment | 1 | 0.16037 | 0.7138 | 8457 |
| Treatment**Individual* | 5 | 0.26691 | 0.903 | 9963 |
| Sampling moment**Individual* | 4 | 0.20349 | 0.9241 | 9961 |
| Residuals | 3 |  |  |  |
| Total | 21 |  |  |  |
| **Dihydro-ß-Ionone** | df | Pseudo-F | p | Unique perms |
| Treatment | 1 | 2.35E-02 | 0.8787 | 9827 |
| Sampling moment | 1 | 4.0379 | 0.1112 | 9782 |
| *Individual* | 6 | 2.0221 | 0.3009 | 9969 |
| Treatment*Sampling moment | 1 | 5.55E-02 | 0.8275 | 9786 |
| Treatment**Individual* | 5 | 0.49265 | 0.7781 | 9970 |
| Sampling moment**Individual* | 4 | 2.0082 | 0.2931 | 9971 |
| Residuals | 3 |  |  |  |
| Total | 21 |  |  |  |
| **β-Ionone** | df | Pseudo-F | p | Unique perms |
| Treatment | 1 | 0.41468 | 0.5472 | 9796 |
| Sampling moment | 1 | 4.277 | 0.1206 | 9372 |
| *Individual* | 6 | 0.60009 | 0.7212 | 9963 |
| Treatment*Sampling moment | 1 | 1.83E-02 | 0.9005 | 9849 |
| Treatment**Individual* | 5 | 0.59008 | 0.7247 | 9967 |
| Sampling moment**Individual* | 4 | 0.63787 | 0.6721 | 9968 |
| Residuals | 3 |  |  |  |
| Total | 21 |  |  |  |
| **(*E*.*E*)-α-Farnesene** | df | Pseudo-F | p | Unique perms |
| Treatment | 1 | 0.66534 | 0.4966 | 9755 |
| Sampling moment | 1 | 0.17491 | 0.6961 | 9271 |
| *Individual* | 6 | 49.634 | **0.0081** | 9961 |
| Treatment*Sampling moment | 1 | 2.5278 | 0.2162 | 8459 |
| Treatment**Individual* | 5 | 2.8139 | 0.2138 | 9977 |
| Sampling moment**Individual* | 4 | 4.7153 | 0.1204 | 9966 |
| Residuals | 3 |  |  |  |
| Total | 21 |  |  |  |
| *Byrsonima intermedia* |  |  |  |  |
| **Dimethyldisulfide** | df | Pseudo-F | p | Unique perms |
| Treatment | 1 | 0.30576 | 0.5803 | 48 |
| Sampling moment | 1 | 1 | 0.7195 | 3 |
| *Individual* | 3 | 3.707 | 0.2282 | 9964 |
| Treatment*Sampling moment | 1 | 1 | 0.5147 | 10 |
| Treatment**Individual* | 3 | 0.87658 | 0.5749 | 9936 |
| Sampling moment**Individual* | 2 | 7.53E-02 | 0.9301 | 9359 |
| Residuals | 2 |  |  |  |
| Total | 13 |  |  |  |
| **Hexanal** | df | Pseudo-F | p | Unique perms |
| Treatment | 1 | 2.1058 | 0.392 | 48 |
| Sampling moment | 1 | 1 | 0.7102 | 3 |
| *Individual* | 3 | 227.43 | **0.0071** | 9971 |
| Treatment*Sampling moment | 1 | 1 | 0.517 | 10 |
| Treatment**Individual* | 3 | 36.645 | **0.024** | 9315 |
| Sampling moment**Individual* | 2 | 6.3524 | 0.1385 | 9404 |
| Residuals | 2 |  |  |  |
| Total | 13 |  |  |  |
| **(*Z*)-2-Hexenal** | df | Pseudo-F | p | Unique perms |
| Treatment | 1 | 0.29412 | 0.5675 | 48 |
| Sampling moment | 1 | 1 | 0.7101 | 3 |
| *Individual* | 3 | 1.0226 | 0.505 | 9971 |
| Treatment*Sampling moment | 1 | 1 | 0.5199 | 10 |
| Treatment**Individual* | 3 | 0.83333 | 0.5932 | 1403 |
| Sampling moment**Individual* | 2 | 1.2272 | 0.4341 | 9349 |
| Residuals | 2 |  |  |  |
| Total | 13 |  |  |  |
| **1-Methoxy-2-propyl acetate** | df | Pseudo-F | p | Unique perms |
| Treatment | 1 | 1.6667 | 0.4674 | 4 |
| Sampling moment | 1 | Denominator is 0 |  |  |
| *Individual* | 3 | Denominator is 0 |  |  |
| Treatment*Sampling moment | 1 | Denominator is 0 |  |  |
| Treatment**Individual* | 3 | Denominator is 0 |  |  |
| Sampling moment**Individual* | 2 | Denominator is 0 |  |  |
| Residuals | 2 |  |  |  |
| Total | 13 |  |  |  |
| **α-Pinene** | df | Pseudo-F | p | Unique perms |
| Treatment | 1 | 0.18906 | 0.6641 | 48 |
| Sampling moment | 1 | 1 | 0.7031 | 3 |
| *Individual* | 3 | 2.342 | 0.3155 | 9964 |
| Treatment*Sampling moment | 1 | 1 | 0.5172 | 10 |
| Treatment**Individual* | 3 | 0.5303 | 0.7096 | 9943 |
| Sampling moment**Individual* | 2 | 2.23E-03 | 0.9935 | 9386 |
| Residuals | 2 |  |  |  |
| Total | 13 |  |  |  |
| **(*Z*)-β-Citronellene** | df | Pseudo-F | p | Unique perms |
| Treatment | 1 | 0.20875 | 0.6522 | 48 |
| Sampling moment | 1 | 1 | 0.71 | 3 |
| *Individual* | 3 | 2.2989 | 0.3164 | 9970 |
| Treatment*Sampling moment | 1 | 1 | 0.5161 | 10 |
| Treatment**Individual* | 3 | 0.57749 | 0.6947 | 9952 |
| Sampling moment**Individual* | 2 | 1.47E-04 | 0.9953 | 9302 |
| Residuals | 2 |  |  |  |
| Total | 13 |  |  |  |
| **1-Decanol** | df | Pseudo-F | p | Unique perms |
| Treatment | 1 | 6.57E-02 | 0.7957 | 48 |
| Sampling moment | 1 | 1 | 0.7135 | 3 |
| *Individual* | 3 | 5.588 | 0.1528 | 9969 |
| Treatment*Sampling moment | 1 | 1 | 0.5185 | 10 |
| Treatment**Individual* | 3 | 0.29272 | 0.8435 | 9941 |
| Sampling moment**Individual* | 2 | 3.74E-02 | 0.9596 | 9355 |
| Residuals | 2 |  |  |  |
| Total | 13 |  |  |  |
| **Benzaldehyde** | df | Pseudo-F | p | Unique perms |
| Treatment | 1 | 1.4286 | 0.4077 | 48 |
| Sampling moment | 1 | 1 | 0.7154 | 3 |
| *Individual* | 3 | 5.6202 | 0.1683 | 9858 |
| Treatment*Sampling moment | 1 | 1 | 0.5179 | 10 |
| Treatment**Individual* | 3 | 0.66667 | 0.6623 | 503 |
| Sampling moment**Individual* | 2 | 8.4303 | 0.1099 | 9362 |
| Residuals | 2 |  |  |  |
| Total | 13 |  |  |  |
| **6-methyl-5-hepten-2-one** | df | Pseudo-F | p | Unique perms |
| Treatment | 1 | 1.4286 | 0.4012 | 48 |
| Sampling moment | 1 | 1 | 0.713 | 3 |
| *Individual* | 3 | 3.6331 | 0.2363 | 9835 |
| Treatment*Sampling moment | 1 | 1 | 0.5186 | 10 |
| Treatment**Individual* | 3 | 0.66667 | 0.662 | 508 |
| Sampling moment**Individual* | 2 | 5.4497 | 0.1491 | 9331 |
| Residuals | 2 |  |  |  |
| Total | 13 |  |  |  |
| **Benzyl alcohol** | df | Pseudo-F | p | Unique perms |
| Treatment | 1 | 1.4286 | 0.3948 | 48 |
| Sampling moment | 1 | 1 | 0.7106 | 3 |
| *Individual* | 3 | 1.1959 | 0.4687 | 9844 |
| Treatment*Sampling moment | 1 | 1 | 0.5138 | 10 |
| Treatment**Individual* | 3 | 0.66667 | 0.6731 | 508 |
| Sampling moment**Individual* | 2 | 1.7939 | 0.358 | 9407 |
| Residuals | 2 |  |  |  |
| Total | 13 |  |  |  |
| **(*Z*)-β-Ocimene** | df | Pseudo-F | p | Unique perms |
| Treatment | 1 | 1.6667 | 0.4672 | 4 |
| Sampling moment | 1 | Denominator is 0 |  |  |
| *Individual* | 3 | Denominator is 0 |  |  |
| Treatment*Sampling moment | 1 | Denominator is 0 |  |  |
| Treatment**Individual* | 3 | Denominator is 0 |  |  |
| Sampling moment**Individual* | 2 | Denominator is 0 |  |  |
| Residuals | 2 |  |  |  |
| Total | 13 |  |  |  |
| **Eucalyptol** | df | Pseudo-F | p | Unique perms |
| Treatment | 1 | 0.13569 | 0.8021 | 48 |
| Sampling moment | 1 | 1 | 0.7122 | 3 |
| *Individual* | 3 | 3.7162 | 0.2231 | 9973 |
| Treatment*Sampling moment | 1 | 1 | 0.5226 | 10 |
| Treatment**Individual* | 3 | 0.41772 | 0.7632 | 9962 |
| Sampling moment**Individual* | 2 | 5.55E-03 | 0.9931 | 9379 |
| Residuals | 2 |  |  |  |
| Total | 13 |  |  |  |
| **(*E*)-β-Ocimene** | df | Pseudo-F | p | Unique perms |
| Treatment | 1 | 2.1774 | 0.3498 | 48 |
| Sampling moment | 1 | 1 | 0.7015 | 3 |
| *Individual* | 3 | 305.7 | **0.0057** | 9963 |
| Treatment*Sampling moment | 1 | 1 | 0.5127 | 10 |
| Treatment**Individual* | 3 | 26.732 | **0.0371** | 9954 |
| Sampling moment**Individual* | 2 | 9.3199 | 0.0946 | 9272 |
| Residuals | 2 |  |  |  |
| Total | 13 |  |  |  |
| **1-Decanol** | df | Pseudo-F | p | Unique perms |
| Treatment | 1 | 0.29412 | 0.5779 | 48 |
| Sampling moment | 1 | 1 | 0.7179 | 3 |
| *Individual* | 3 | 0.89467 | 0.5494 | 9961 |
| Treatment*Sampling moment | 1 | 1 | 0.5234 | 10 |
| Treatment**Individual* | 3 | 0.83333 | 0.5907 | 1401 |
| Sampling moment**Individual* | 2 | 1.0736 | 0.4445 | 9312 |
| Residuals | 2 |  |  |  |
| Total | 13 |  |  |  |
| **(*Z*)-linalool oxide furanoid** | df | Pseudo-F | p | Unique perms |
| Treatment | 1 | 1.9027 | 0.4413 | 605 |
| Sampling moment | 1 | 1.243 | 0.4067 | 34 |
| *Individual* | 3 | 5323.4 | **0.0014** | 9971 |
| Treatment*Sampling moment | 1 | 0.29938 | 0.6462 | 937 |
| Treatment**Individual* | 3 | 596.84 | **0.0021** | 9971 |
| Sampling moment**Individual* | 2 | 28.44 | **0.0377** | 9955 |
| Residuals | 2 |  |  |  |
| Total | 13 |  |  |  |
| **(*E*)-linalool oxide furanoid** | df | Pseudo-F | p | Unique perms |
| Treatment | 1 | 1.6667 | 0.4699 | 4 |
| Sampling moment | 1 | Denominator is 0 |  |  |
| *Individual* | 3 | Denominator is 0 |  |  |
| Treatment*Sampling moment | 1 | Denominator is 0 |  |  |
| Treatment**Individual* | 3 | Denominator is 0 |  |  |
| Sampling moment**Individual* | 2 | Denominator is 0 |  |  |
| Residuals | 2 |  |  |  |
| Total | 13 |  |  |  |
| **(*Z*)-4.8-Dimethyl-1.3.7-nonatriene** | df | Pseudo-F | p | Unique perms |
| Treatment | 1 | 1.6667 | 0.4649 | 4 |
| Sampling moment | 1 | Denominator is 0 |  |  |
| *Individual* | 3 | Denominator is 0 |  |  |
| Treatment*Sampling moment | 1 | Denominator is 0 |  |  |
| Treatment**Individual* | 3 | Denominator is 0 |  |  |
| Sampling moment**Individual* | 2 | Denominator is 0 |  |  |
| Residuals | 2 |  |  |  |
| Total | 13 |  |  |  |
| **Linalool** | df | Pseudo-F | p | Unique perms |
| Treatment | 1 | 1.7971 | 0.4478 | 47 |
| Sampling moment | 1 | 1 | 0.7178 | 3 |
| *Individual* | 3 | 2024.4 | **0.0053** | 9965 |
| Treatment*Sampling moment | 1 | 1 | 0.5155 | 10 |
| Treatment**Individual* | 3 | 431.92 | **0.0042** | 9947 |
| Sampling moment**Individual* | 2 | 3.6579 | 0.2157 | 9336 |
| Residuals | 2 |  |  |  |
| Total | 13 |  |  |  |
| **(*E*)-4.8-dimethyl-1.3.7-nonatriene** | df | Pseudo-F | p | Unique perms |
| Treatment | 1 | 1.6723 | 0.4367 | 39 |
| Sampling moment | 1 | 1 | 0.7227 | 3 |
| *Individual* | 3 | 1.99E+05 | **0.0013** | 9961 |
| Treatment*Sampling moment | 1 | 1 | 0.5118 | 10 |
| Treatment**Individual* | 3 | 27943 | **0.0066** | 9969 |
| Sampling moment**Individual* | 2 | 7.4716 | 0.125 | 9354 |
| Residuals | 2 |  |  |  |
| Total | 13 |  |  |  |
| **Methyl octanoate** | df | Pseudo-F | p | Unique perms |
| Treatment | 1 | 0.29412 | 0.5718 | 48 |
| Sampling moment | 1 | 1 | 0.7183 | 3 |
| *Individual* | 3 | 1.233 | 0.4609 | 9970 |
| Treatment*Sampling moment | 1 | 1 | 0.5262 | 10 |
| Treatment**Individual* | 3 | 0.83333 | 0.5842 | 1422 |
| Sampling moment**Individual* | 2 | 1.4796 | 0.396 | 9299 |
| Residuals | 2 |  |  |  |
| Total | 13 |  |  |  |
| **Ocimene derivative** | df | Pseudo-F | p | Unique perms |
| Treatment | 1 | 1.4286 | 0.3948 | 48 |
| Sampling moment | 1 | 1 | 0.7049 | 3 |
| *Individual* | 3 | 1.1747 | 0.479 | 9850 |
| Treatment*Sampling moment | 1 | 1 | 0.521 | 10 |
| Treatment**Individual* | 3 | 0.66667 | 0.6533 | 505 |
| Sampling moment**Individual* | 2 | 1.762 | 0.361 | 9379 |
| Residuals | 2 |  |  |  |
| Total | 13 |  |  |  |
| ***allo*-Ocimene** | df | Pseudo-F | p | Unique perms |
| Treatment | 1 | 1.8631 | 0.4448 | 48 |
| Sampling moment | 1 | 1 | 0.7129 | 3 |
| *Individual* | 3 | 471.53 | **0.0089** | 9971 |
| Treatment*Sampling moment | 1 | 1 | 0.5203 | 10 |
| Treatment**Individual* | 3 | 189.15 | **0.0061** | 9958 |
| Sampling moment**Individual* | 2 | 1.196 | 0.4464 | 9371 |
| Residuals | 2 |  |  |  |
| Total | 13 |  |  |  |
| ***p*-1.3.8-Mentatriene** | df | Pseudo-F | p | Unique perms |
| Treatment | 1 | 1.4286 | 0.3995 | 48 |
| Sampling moment | 1 | 1 | 0.7124 | 3 |
| *Individual* | 3 | 2.0222 | 0.3416 | 9838 |
| Treatment*Sampling moment | 1 | 1 | 0.5147 | 10 |
| Treatment**Individual* | 3 | 0.66667 | 0.6632 | 506 |
| Sampling moment**Individual* | 2 | 3.0333 | 0.2445 | 9368 |
| Residuals | 2 |  |  |  |
| Total | 13 |  |  |  |
| **Phenylacetonitrile** | df | Pseudo-F | p | Unique perms |
| Treatment | 1 | 1.1737 | 0.4837 | 688 |
| Sampling moment | 1 | 3.4431 | 0.2174 | 34 |
| *Individual* | 3 | 26.345 | **0.041** | 9981 |
| Treatment*Sampling moment | 1 | 0.75992 | 0.4969 | 920 |
| Treatment**Individual* | 3 | 3.4778 | 0.2296 | 9977 |
| Sampling moment**Individual* | 2 | 0.98696 | 0.5037 | 9958 |
| Residuals | 2 |  |  |  |
| Total | 13 |  |  |  |
| **(*Z*)-linalool oxide pyranoid** | df | Pseudo-F | p | Unique perms |
| Treatment | 1 | 1.5667 | 0.4588 | 634 |
| Sampling moment | 1 | 1.8202 | 0.384 | 34 |
| *Individual* | 3 | 147.13 | **0.0109** | 9968 |
| Treatment*Sampling moment | 1 | 0.16994 | 0.7194 | 942 |
| Treatment**Individual* | 3 | 146.4 | **0.0061** | 9967 |
| Sampling moment**Individual* | 2 | 2.0872 | 0.3218 | 9923 |
| Residuals | 2 |  |  |  |
| Total | 13 |  |  |  |
| **Methyl salicylate** | df | Pseudo-F | p | Unique perms |
| Treatment | 1 | 1.6667 | 0.4679 | 4 |
| Sampling moment | 1 | Denominator is 0 |  |  |
| *Individual* | 3 | Denominator is 0 |  |  |
| Treatment*Sampling moment | 1 | Denominator is 0 |  |  |
| Treatment**Individual* | 3 | Denominator is 0 |  |  |
| Sampling moment**Individual* | 2 | Denominator is 0 |  |  |
| Residuals | 2 |  |  |  |
| Total | 13 |  |  |  |
| ***p*-Anisaldehyde** | df | Pseudo-F | p | Unique perms |
| Treatment | 1 | 1.6667 | 0.4677 | 4 |
| Sampling moment | 1 | Denominator is 0 |  |  |
| *Individual* | 3 | Denominator is 0 |  |  |
| Treatment*Sampling moment | 1 | Denominator is 0 |  |  |
| Treatment**Individual* | 3 | Denominator is 0 |  |  |
| Sampling moment**Individual* | 2 | Denominator is 0 |  |  |
| Residuals | 2 |  |  |  |
| Total | 13 |  |  |  |
| **Neodihydrocarveol** | df | Pseudo-F | p | Unique perms |
| Treatment | 1 | 0.29412 | 0.572 | 48 |
| Sampling moment | 1 | 1 | 0.7074 | 3 |
| *Individual* | 3 | 3.2372 | 0.2523 | 9966 |
| Treatment*Sampling moment | 1 | 1 | 0.5147 | 10 |
| Treatment**Individual* | 3 | 0.83333 | 0.581 | 1404 |
| Sampling moment**Individual* | 2 | 3.8846 | 0.2038 | 9327 |
| Residuals | 2 |  |  |  |
| Total | 13 |  |  |  |
| **(*Z*)-Dihydrocarveol** | df | Pseudo-F | p | Unique perms |
| Treatment | 1 | 1.36E-02 | 0.8 | 48 |
| Sampling moment | 1 | 1 | 0.7196 | 3 |
| *Individual* | 3 | 0.82941 | 0.5802 | 9968 |
| Treatment*Sampling moment | 1 | 1 | 0.514 | 10 |
| Treatment**Individual* | 3 | 0.19848 | 0.8926 | 9959 |
| Sampling moment**Individual* | 2 | 0.12785 | 0.8815 | 9380 |
| Residuals | 2 |  |  |  |
| Total | 13 |  |  |  |
| **Lavandulyl acetate** | df | Pseudo-F | p | Unique perms |
| Treatment | 1 | 1.6667 | 0.4682 | 4 |
| Sampling moment | 1 | Denominator is 0 |  |  |
| *Individual* | 3 | Denominator is 0 |  |  |
| Treatment*Sampling moment | 1 | Denominator is 0 |  |  |
| Treatment**Individual* | 3 | Denominator is 0 |  |  |
| Sampling moment**Individual* | 2 | Denominator is 0 |  |  |
| Residuals | 2 |  |  |  |
| Total | 13 |  |  |  |
| **Indole** | df | Pseudo-F | p | Unique perms |
| Treatment | 1 | 0.37063 | 0.5655 | 48 |
| Sampling moment | 1 | 1 | 0.7131 | 3 |
| *Individual* | 3 | 59.25 | **0.0246** | 9976 |
| Treatment*Sampling moment | 1 | 1 | 0.5166 | 10 |
| Treatment**Individual* | 3 | 1.1713 | 0.4847 | 9965 |
| Sampling moment**Individual* | 2 | 51.924 | **0.0195** | 9368 |
| Residuals | 2 |  |  |  |
| Total | 13 |  |  |  |
| **Chrysanthenone** | df | Pseudo-F | p | Unique perms |
| Treatment | 1 | 0.24315 | 0.5866 | 48 |
| Sampling moment | 1 | 1 | 0.7103 | 3 |
| *Individual* | 3 | 2.055 | 0.3397 | 9960 |
| Treatment*Sampling moment | 1 | 1 | 0.5168 | 10 |
| Treatment**Individual* | 3 | 0.66934 | 0.6449 | 9944 |
| Sampling moment**Individual* | 2 | 3.73E-02 | 0.9592 | 9347 |
| Residuals | 2 |  |  |  |
| Total | 13 |  |  |  |
| **Methyl decanoate** | df | Pseudo-F | p | Unique perms |
| Treatment | 1 | 1.5558 | 0.316 | 685 |
| Sampling moment | 1 | 0.81649 | 0.6253 | 34 |
| *Individual* | 3 | 0.66967 | 0.6175 | 9970 |
| Treatment*Sampling moment | 1 | 0.95347 | 0.4867 | 863 |
| Treatment**Individual* | 3 | 0.34711 | 0.8008 | 9976 |
| Sampling moment**Individual* | 2 | 0.53847 | 0.6507 | 9957 |
| Residuals | 2 |  |  |  |
| Total | 13 |  |  |  |
| **Tetradecane** | df | Pseudo-F | p | Unique perms |
| Treatment | 1 | 0.43774 | 0.5667 | 44 |
| Sampling moment | 1 | 1 | 0.7126 | 3 |
| *Individual* | 3 | 5.7627 | 0.1512 | 9965 |
| Treatment*Sampling moment | 1 | 1 | 0.5134 | 10 |
| Treatment**Individual* | 3 | 4.22E-02 | 0.9862 | 9942 |
| Sampling moment**Individual* | 2 | 3.7003 | 0.2153 | 9376 |
| Residuals | 2 |  |  |  |
| Total | 13 |  |  |  |
| **β-Elemene** | df | Pseudo-F | p | Unique perms |
| Treatment | 1 | 0.90801 | 0.4725 | 44 |
| Sampling moment | 1 | 1 | 0.7081 | 3 |
| *Individual* | 3 | 10610 | **0.001** | 9970 |
| Treatment*Sampling moment | 1 | 1 | 0.516 | 10 |
| Treatment**Individual* | 3 | 512.14 | **0.0028** | 9950 |
| Sampling moment**Individual* | 2 | 298.15 | **0.0056** | 9327 |
| Residuals | 2 |  |  |  |
| Total | 13 |  |  |  |
| **β-Caryophyllene** | df | Pseudo-F | p | Unique perms |
| Treatment | 1 | 1.2621 | 0.4527 | 1395 |
| Sampling moment | 1 | 1.562 | 0.331 | 206 |
| *Individual* | 3 | 4.8409 | 0.1824 | 9979 |
| Treatment*Sampling moment | 1 | 0.92489 | 0.4411 | 7784 |
| Treatment**Individual* | 3 | 0.54857 | 0.6911 | 9977 |
| Sampling moment**Individual* | 2 | 0.65621 | 0.6013 | 9948 |
| Residuals | 2 |  |  |  |
| Total | 13 |  |  |  |
| **(*E*)-Caryophyllene** | df | Pseudo-F | p | Unique perms |
| Treatment | 1 | 0.61855 | 0.5817 | 48 |
| Sampling moment | 1 | 1 | 0.7142 | 3 |
| *Individual* | 3 | 864.74 | **0.0099** | 9967 |
| Treatment*Sampling moment | 1 | 1 | 0.5143 | 10 |
| Treatment**Individual* | 3 | 4.8903 | 0.1772 | 9957 |
| Sampling moment**Individual* | 2 | 1052.8 | **0.0046** | 9311 |
| Residuals | 2 |  |  |  |
| Total | 13 |  |  |  |
| **α-Caryophyllene** | df | Pseudo-F | p | Unique perms |
| Treatment | 1 | 2.5888 | 0.2285 | 1351 |
| Sampling moment | 1 | 1.1646 | 0.3578 | 200 |
| *Individual* | 3 | 1.0815 | 0.5192 | 9977 |
| Treatment*Sampling moment | 1 | 0.93212 | 0.4921 | 7205 |
| Treatment**Individual* | 3 | 0.91099 | 0.5561 | 9967 |
| Sampling moment**Individual* | 2 | 1.1303 | 0.4522 | 9945 |
| Residuals | 2 |  |  |  |
| Total | 13 |  |  |  |
| **Germacrene D** | df | Pseudo-F | p | Unique perms |
| Treatment | 1 | 1.4286 | 0.4028 | 48 |
| Sampling moment | 1 | 1 | 0.7256 | 3 |
| *Individual* | 3 | 0.67279 | 0.6483 | 9719 |
| Treatment*Sampling moment | 1 | 1 | 0.5224 | 10 |
| Treatment**Individual* | 3 | 0.66667 | 0.6663 | 506 |
| Sampling moment**Individual* | 2 | 1.0092 | 0.4566 | 8165 |
| Residuals | 2 |  |  |  |
| Total | 13 |  |  |  |
| **Nerolidol** | df | Pseudo-F | p | Unique perms |
| Treatment | 1 | 1.7575 | 0.4354 | 44 |
| Sampling moment | 1 | 1 | 0.7225 | 3 |
| *Individual* | 3 | 7498.8 | **0.0009** | 9975 |
| Treatment*Sampling moment | 1 | 1 | 0.5201 | 10 |
| Treatment**Individual* | 3 | 2802.1 | **0.0041** | 9971 |
| Sampling moment**Individual* | 2 | 6.677 | 0.1271 | 9342 |
| Residuals | 2 |  |  |  |
| Total | 13 |  |  |  |
| **Caryophyllene oxide derivative** | df | Pseudo-F | p | Unique perms |
| Treatment | 1 | 1.1567 | 0.3573 | 699 |
| Sampling moment | 1 | 0.23378 | 0.6571 | 34 |
| *Individual* | 3 | 17.845 | 0.0578 | 9967 |
| Treatment*Sampling moment | 1 | 3.8375 | 0.1884 | 934 |
| Treatment**Individual* | 3 | 2.7558 | 0.2807 | 9976 |
| Sampling moment**Individual* | 2 | 11.84 | 0.079 | 9924 |
| Residuals | 2 |  |  |  |
| Total | 13 |  |  |  |
| **Kessane** | df | Pseudo-F | p | Unique perms |
| Treatment | 1 | 1.6772 | 0.4435 | 401 |
| Sampling moment | 1 | 0.19957 | 0.6514 | 34 |
| *Individual* | 3 | 1.13E+05 | **0.0023** | 9969 |
| Treatment*Sampling moment | 1 | 3.3288 | 0.2272 | 939 |
| Treatment**Individual* | 3 | 1.14E+05 | **0.0046** | 9959 |
| Sampling moment**Individual* | 2 | 14.542 | 0.067 | 9962 |
| Residuals | 2 |  |  |  |
| Total | 13 |  |  |  |
| *Centrosema pubescens* |  |  |  |  |
| **4-Methyl-3-pentene-2-ol** | df | Pseudo-F | p | Unique perms |
| Treatment | 1 | 9.34E-02 | 0.7603 | 207 |
| Sampling moment | 1 | 3.5941 | 0.1999 | 207 |
| *Individual* | 3 | 5.2191 | 0.1117 | 9961 |
| Treatment*Sampling moment | 1 | 3.45E-02 | 0.7919 | 7560 |
| Treatment**Individual* | 3 | 1.3022 | 0.3795 | 9955 |
| Sampling moment**Individual* | 3 | 1.8282 | 0.3099 | 9960 |
| Residuals | 3 |  |  |  |
| Total | 15 |  |  |  |
| **a 2-Methyl-2-vinyl-5-hydroxytetrahydrofuran (RI 928)** | df | Pseudo-F | p | Unique perms |
| Treatment | 1 | 1.2778 | 0.3209 | 207 |
| Sampling moment | 1 | 9.74E-05 | 0.9142 | 209 |
| *Individual* | 3 | 6.1747 | 0.0972 | 9959 |
| Treatment*Sampling moment | 1 | 0.50358 | 0.5687 | 7537 |
| Treatment**Individual* | 3 | 0.24239 | 0.8596 | 9973 |
| Sampling moment**Individual* | 3 | 8.66E-02 | 0.9633 | 9961 |
| Residuals | 3 |  |  |  |
| Total | 15 |  |  |  |
| **a 2-Methyl-2-vinyl-5-hydroxytetrahydrofuran (RI 946)** | df | Pseudo-F | p | Unique perms |
| Treatment | 1 | 1.35E-03 | 0.7959 | 20 |
| Sampling moment | 1 | 2.8529 | 0.2432 | 20 |
| *Individual* | 3 | 4.2504 | 0.1563 | 9753 |
| Treatment*Sampling moment | 1 | 0.65475 | 0.5155 | 613 |
| Treatment**Individual* | 3 | 5.31E-02 | 0.9829 | 9960 |
| Sampling moment**Individual* | 3 | 4.81E-02 | 0.9842 | 9970 |
| Residuals | 3 |  |  |  |
| Total | 15 |  |  |  |
| **β-Myrcene** | df | Pseudo-F | p | Unique perms |
| Treatment | 1 | 2.8725 | 0.2047 | 425 |
| Sampling moment | 1 | 5.7658 | 0.1127 | 425 |
| *Individual* | 3 | 1.6254 | 0.3241 | 9953 |
| Treatment*Sampling moment | 1 | 2.9026 | 0.1928 | 9208 |
| Treatment**Individual* | 3 | 0.59765 | 0.6671 | 9965 |
| Sampling moment**Individual* | 3 | 0.31164 | 0.8269 | 9961 |
| Residuals | 3 |  |  |  |
| Total | 15 |  |  |  |
| **δ-3-Carene** | df | Pseudo-F | p | Unique perms |
| Treatment | 1 | 1 | 0.5731 | 3 |
| Sampling moment | 1 | 1 | 0.5702 | 3 |
| *Individual* | 3 | 1.4231 | 0.3127 | 2766 |
| Treatment*Sampling moment | 1 | 1 | 0.4441 | 7 |
| Treatment**Individual* | 3 | 1 | 0.5209 | 687 |
| Sampling moment**Individual* | 3 | 1.4231 | 0.3764 | 9891 |
| Residuals | 3 |  |  |  |
| Total | 15 |  |  |  |
| **Benzyl alcohol** | df | Pseudo-F | p | Unique perms |
| Treatment | 1 | 0.82242 | 0.5419 | 207 |
| Sampling moment | 1 | 7.2472 | 0.0984 | 207 |
| *Individual* | 3 | 2.9204 | 0.1968 | 9952 |
| Treatment*Sampling moment | 1 | 0.81228 | 0.4576 | 7444 |
| Treatment**Individual* | 3 | 1.1159 | 0.4521 | 9965 |
| Sampling moment**Individual* | 3 | 1.5031 | 0.3681 | 9974 |
| Residuals | 3 |  |  |  |
| Total | 15 |  |  |  |
| **Limonene** | df | Pseudo-F | p | Unique perms |
| Treatment | 1 | 1 | 0.5752 | 3 |
| Sampling moment | 1 | 1 | 0.575 | 3 |
| *Individual* | 3 | 2.365 | 0.204 | 2748 |
| Treatment*Sampling moment | 1 | 1 | 0.4455 | 7 |
| Treatment**Individual* | 3 | 1 | 0.5246 | 700 |
| Sampling moment**Individual* | 3 | 2.365 | 0.2385 | 9874 |
| Residuals | 3 |  |  |  |
| Total | 15 |  |  |  |
| **(*Z*)-β-Ocimene** | df | Pseudo-F | p | Unique perms |
| Treatment | 1 | 4.2008 | 0.1537 | 207 |
| Sampling moment | 1 | 2.5831 | 0.2335 | 207 |
| *Individual* | 3 | 5.9917 | 0.0982 | 9964 |
| Treatment*Sampling moment | 1 | 4.2008 | 0.1399 | 7688 |
| Treatment**Individual* | 3 | 1 | 0.4966 | 9724 |
| Sampling moment**Individual* | 3 | 5.9917 | 0.0898 | 9971 |
| Residuals | 3 |  |  |  |
| Total | 15 |  |  |  |
| **Lavender lactone** | df | Pseudo-F | p | Unique perms |
| Treatment | 1 | 0.90539 | 0.4146 | 207 |
| Sampling moment | 1 | 4.1565 | 0.1477 | 207 |
| *Individual* | 3 | 40.696 | **0.0166** | 9962 |
| Treatment*Sampling moment | 1 | 0.52539 | 0.5547 | 7549 |
| Treatment**Individual* | 3 | 0.3721 | 0.7781 | 9960 |
| Sampling moment**Individual* | 3 | 2.023 | 0.2848 | 9979 |
| Residuals | 3 |  |  |  |
| Total | 15 |  |  |  |
| **(*E*)-β-Ocimene** | df | Pseudo-F | p | Unique perms |
| Treatment | 1 | 3.4793 | 0.183 | 201 |
| Sampling moment | 1 | 2.3051 | 0.2528 | 206 |
| *Individual* | 3 | 6.9613 | 0.0839 | 9965 |
| Treatment*Sampling moment | 1 | 3.547 | 0.1604 | 7432 |
| Treatment**Individual* | 3 | 1.3135 | 0.4014 | 9957 |
| Sampling moment**Individual* | 3 | 5.2568 | 0.1042 | 9968 |
| Residuals | 3 |  |  |  |
| Total | 15 |  |  |  |
| **γ-Terpinene** | df | Pseudo-F | p | Unique perms |
| Treatment | 1 | 1 | 0.5655 | 3 |
| Sampling moment | 1 | 1 | 0.5657 | 3 |
| *Individual* | 3 | 1.159 | 0.356 | 2742 |
| Treatment*Sampling moment | 1 | 1 | 0.4454 | 7 |
| Treatment**Individual* | 3 | 1 | 0.5189 | 693 |
| Sampling moment**Individual* | 3 | 1.159 | 0.4427 | 9857 |
| Residuals | 3 |  |  |  |
| Total | 15 |  |  |  |
| **(*Z*)-linalool oxide furanoid** | df | Pseudo-F | p | Unique perms |
| Treatment | 1 | 0.51925 | 0.5498 | 190 |
| Sampling moment | 1 | 5.6504 | 0.1205 | 246 |
| *Individual* | 3 | 4.598 | 0.1295 | 9954 |
| Treatment*Sampling moment | 1 | 0.60236 | 0.5239 | 7130 |
| Treatment**Individual* | 3 | 0.70564 | 0.6262 | 9959 |
| Sampling moment**Individual* | 3 | 0.57969 | 0.6646 | 9958 |
| Residuals | 3 |  |  |  |
| Total | 15 |  |  |  |
| **Methyl phenethylether** | df | Pseudo-F | p | Unique perms |
| Treatment | 1 | 5.47E-02 | 0.8728 | 207 |
| Sampling moment | 1 | 0.30988 | 0.6045 | 207 |
| *Individual* | 3 | 5.8435 | 0.1005 | 9969 |
| Treatment*Sampling moment | 1 | 1.4165 | 0.3277 | 7455 |
| Treatment**Individual* | 3 | 1.369 | 0.4057 | 9968 |
| Sampling moment**Individual* | 3 | 1.9301 | 0.3046 | 9967 |
| Residuals | 3 |  |  |  |
| Total | 15 |  |  |  |
| **(*E*)-linalool oxide furanoid** | df | Pseudo-F | p | Unique perms |
| Treatment | 1 | 0.69452 | 0.5497 | 410 |
| Sampling moment | 1 | 4.3563 | 0.1511 | 410 |
| *Individual* | 3 | 22.982 | **0.0217** | 9959 |
| Treatment*Sampling moment | 1 | 0.13251 | 0.7266 | 9043 |
| Treatment**Individual* | 3 | 7.3347 | 0.0669 | 9957 |
| Sampling moment**Individual* | 3 | 1.7707 | 0.3206 | 9975 |
| Residuals | 3 |  |  |  |
| Total | 15 |  |  |  |
| **α-Terpinolene** | df | Pseudo-F | p | Unique perms |
| Treatment | 1 | 1 | 0.5679 | 3 |
| Sampling moment | 1 | 1 | 0.5695 | 3 |
| *Individual* | 3 | 1.1457 | 0.3433 | 2728 |
| Treatment*Sampling moment | 1 | 1 | 0.4531 | 7 |
| Treatment**Individual* | 3 | 1 | 0.5279 | 693 |
| Sampling moment**Individual* | 3 | 1.1457 | 0.448 | 9862 |
| Residuals | 3 |  |  |  |
| Total | 15 |  |  |  |
| **Linalool** | df | Pseudo-F | p | Unique perms |
| Treatment | 1 | 0.52689 | 0.4976 | 424 |
| Sampling moment | 1 | 0.69636 | 0.4503 | 425 |
| *Individual* | 3 | 1.7747 | 0.3264 | 9963 |
| Treatment*Sampling moment | 1 | 5.04E-05 | 0.9604 | 9137 |
| Treatment**Individual* | 3 | 1.2452 | 0.4305 | 9958 |
| Sampling moment**Individual* | 3 | 1.4233 | 0.3917 | 9968 |
| Residuals | 3 |  |  |  |
| Total | 15 |  |  |  |
| **Hotrienol** | df | Pseudo-F | p | Unique perms |
| Treatment | 1 | 1 | 0.5635 | 3 |
| Sampling moment | 1 | 1 | 0.5634 | 3 |
| *Individual* | 3 | 592.84 | **0.0326** | 2656 |
| Treatment*Sampling moment | 1 | 1 | 0.4455 | 7 |
| Treatment**Individual* | 3 | 1 | 0.5136 | 686 |
| Sampling moment**Individual* | 3 | 592.84 | **0.0049** | 9860 |
| Residuals | 3 |  |  |  |
| Total | 15 |  |  |  |
| **2-Phenylethanol** | df | Pseudo-F | p | Unique perms |
| Treatment | 1 | 0.25461 | 0.6694 | 425 |
| Sampling moment | 1 | 36.949 | **0.0285** | 423 |
| *Individual* | 3 | 3.113 | 0.1861 | 9949 |
| Treatment*Sampling moment | 1 | 0.50747 | 0.5269 | 9168 |
| Treatment**Individual* | 3 | 1.1778 | 0.4417 | 9963 |
| Sampling moment**Individual* | 3 | 0.28153 | 0.8358 | 9968 |
| Residuals | 3 |  |  |  |
| Total | 15 |  |  |  |
| ***allo*-Ocimene** | df | Pseudo-F | p | Unique perms |
| Treatment | 1 | 2.60E-02 | 0.8863 | 207 |
| Sampling moment | 1 | 3.303 | 0.1682 | 207 |
| *Individual* | 3 | 1.4148 | 0.3705 | 9901 |
| Treatment*Sampling moment | 1 | 2.22E-02 | 0.8879 | 7580 |
| Treatment**Individual* | 3 | 1.0083 | 0.4994 | 9971 |
| Sampling moment**Individual* | 3 | 1.4242 | 0.3844 | 9965 |
| Residuals | 3 |  |  |  |
| Total | 15 |  |  |  |
| ***p*-1.3.8-Menthatriene** | df | Pseudo-F | p | Unique perms |
| Treatment | 1 | 0.25097 | 0.6731 | 207 |
| Sampling moment | 1 | 3.7754 | 0.1757 | 207 |
| *Individual* | 3 | 3.193 | 0.1885 | 9956 |
| Treatment*Sampling moment | 1 | 9.31E-02 | 0.7673 | 7621 |
| Treatment**Individual* | 3 | 0.79799 | 0.594 | 9962 |
| Sampling moment**Individual* | 3 | 1.0831 | 0.4718 | 9959 |
| Residuals | 3 |  |  |  |
| Total | 15 |  |  |  |
| ***neoallo*-Ocimene** | df | Pseudo-F | p | Unique perms |
| Treatment | 1 | 2.9743 | 0.2418 | 20 |
| Sampling moment | 1 | 2.9743 | 0.2368 | 20 |
| *Individual* | 3 | 1 | 0.5233 | 2090 |
| Treatment*Sampling moment | 1 | 2.9743 | 0.1947 | 606 |
| Treatment**Individual* | 3 | 1 | 0.5127 | 7683 |
| Sampling moment**Individual* | 3 | 1 | 0.5073 | 7731 |
| Residuals | 3 |  |  |  |
| Total | 15 |  |  |  |
| **1.2-Dimethoxybenzene** | df | Pseudo-F | p | Unique perms |
| Treatment | 1 | 15.104 | 0.0549 | 425 |
| Sampling moment | 1 | 1.0183 | 0.3733 | 425 |
| *Individual* | 3 | 5.0135 | 0.1237 | 9962 |
| Treatment*Sampling moment | 1 | 1.2261 | 0.347 | 9211 |
| Treatment**Individual* | 3 | 0.11259 | 0.9439 | 9969 |
| Sampling moment**Individual* | 3 | 4.6826 | 0.1234 | 9960 |
| Residuals | 3 |  |  |  |
| Total | 15 |  |  |  |
| **(*Z*)-linalool oxide pyranoid** | df | Pseudo-F | p | Unique perms |
| Treatment | 1 | 5.7779 | 0.1246 | 218 |
| Sampling moment | 1 | 0.39839 | 0.5648 | 199 |
| *Individual* | 3 | 11.437 | **0.0423** | 9953 |
| Treatment*Sampling moment | 1 | 0.99509 | 0.3931 | 7647 |
| Treatment**Individual* | 3 | 0.10258 | 0.9496 | 9963 |
| Sampling moment**Individual* | 3 | 5.5688 | 0.0994 | 9964 |
| Residuals | 3 |  |  |  |
| Total | 15 |  |  |  |
| **(*E*)-linalool oxide pyranoid** | df | Pseudo-F | p | Unique perms |
| Treatment | 1 | 2.2679 | 0.2477 | 20 |
| Sampling moment | 1 | 1.3847 | 0.4706 | 20 |
| *Individual* | 3 | 112.29 | **0.0222** | 9693 |
| Treatment*Sampling moment | 1 | 0.35429 | 0.5966 | 601 |
| Treatment**Individual* | 3 | 1.3956 | 0.3954 | 9973 |
| Sampling moment**Individual* | 3 | 39.399 | **0.0079** | 9955 |
| Residuals | 3 |  |  |  |
| Total | 15 |  |  |  |
| **2-Phenylethyl formate** | df | Pseudo-F | p | Unique perms |
| Treatment | 1 | 0.15633 | 0.6888 | 207 |
| Sampling moment | 1 | 5.2799 | 0.125 | 207 |
| *Individual* | 3 | 1.298 | 0.4111 | 9962 |
| Treatment*Sampling moment | 1 | 0.15633 | 0.7173 | 7668 |
| Treatment**Individual* | 3 | 1 | 0.5027 | 9710 |
| Sampling moment**Individual* | 3 | 1.298 | 0.423 | 9968 |
| Residuals | 3 |  |  |  |
| Total | 15 |  |  |  |
| **(*Z*)-3-Hexenyl butyrate** | df | Pseudo-F | p | Unique perms |
| Treatment | 1 | 1.6514 | 0.4121 | 207 |
| Sampling moment | 1 | 0.66888 | 0.57 | 207 |
| *Individual* | 3 | 6.2357 | 0.0973 | 9955 |
| Treatment*Sampling moment | 1 | 0.54716 | 0.5548 | 7524 |
| Treatment**Individual* | 3 | 0.7626 | 0.5826 | 9959 |
| Sampling moment**Individual* | 3 | 7.5144 | 0.0676 | 9967 |
| Residuals | 3 |  |  |  |
| Total | 15 |  |  |  |
| **α-Terpineol** | df | Pseudo-F | p | Unique perms |
| Treatment | 1 | 1 | 0.57 | 3 |
| Sampling moment | 1 | 1 | 0.5679 | 3 |
| *Individual* | 3 | 23.187 | **0.0358** | 2772 |
| Treatment*Sampling moment | 1 | 1 | 0.4332 | 7 |
| Treatment**Individual* | 3 | 2.7539 | 0.2008 | 9860 |
| Sampling moment**Individual* | 3 | 0.47359 | 0.74 | 9878 |
| Residuals | 3 |  |  |  |
| Total | 15 |  |  |  |
| **3-Phenylpropanenitrile** | df | Pseudo-F | p | Unique perms |
| Treatment | 1 | 1 | 0.5712 | 3 |
| Sampling moment | 1 | 1 | 0.5684 | 3 |
| *Individual* | 3 | 485.54 | **0.0378** | 2715 |
| Treatment*Sampling moment | 1 | 1 | 0.443 | 7 |
| Treatment**Individual* | 3 | 1 | 0.5179 | 697 |
| Sampling moment**Individual* | 3 | 485.54 | **0.0058** | 9850 |
| Residuals | 3 |  |  |  |
| Total | 15 |  |  |  |
| **Geraniol** | df | Pseudo-F | p | Unique perms |
| Treatment | 1 | 1 | 0.5713 | 3 |
| Sampling moment | 1 | 1 | 0.5721 | 3 |
| *Individual* | 3 | 1.7528 | 0.2676 | 2795 |
| Treatment*Sampling moment | 1 | 1 | 0.4442 | 7 |
| Treatment**Individual* | 3 | 1 | 0.5217 | 714 |
| Sampling moment**Individual* | 3 | 1.7528 | 0.3132 | 9870 |
| Residuals | 3 |  |  |  |
| Total | 15 |  |  |  |
| **2-Phenylethyl acetate** | df | Pseudo-F | p | Unique perms |
| Treatment | 1 | 2.3554 | 0.2429 | 207 |
| Sampling moment | 1 | 3.6706 | 0.1627 | 207 |
| *Individual* | 3 | 2.7386 | 0.2223 | 9959 |
| Treatment*Sampling moment | 1 | 2.3554 | 0.2291 | 7572 |
| Treatment**Individual* | 3 | 1 | 0.4955 | 9727 |
| Sampling moment**Individual* | 3 | 2.7386 | 0.2164 | 9972 |
| Residuals | 3 |  |  |  |
| Total | 15 |  |  |  |
| ***p*-Anisaldehyde** | df | Pseudo-F | p | Unique perms |
| Treatment | 1 | 2.0099 | 0.2985 | 20 |
| Sampling moment | 1 | 1.4443 | 0.4815 | 20 |
| *Individual* | 3 | 8.1181 | 0.0688 | 9738 |
| Treatment*Sampling moment | 1 | 2.0666 | 0.241 | 604 |
| Treatment**Individual* | 3 | 1.6727 | 0.3342 | 9960 |
| Sampling moment**Individual* | 3 | 4.8341 | 0.1157 | 9967 |
| Residuals | 3 |  |  |  |
| Total | 15 |  |  |  |
| ***p*-Anisyl alcohol** | df | Pseudo-F | p | Unique perms |
| Treatment | 1 | 1 | 0.5706 | 3 |
| Sampling moment | 1 | 1 | 0.5611 | 3 |
| *Individual* | 3 | 270.77 | **0.0428** | 2743 |
| Treatment*Sampling moment | 1 | 1 | 0.4445 | 7 |
| Treatment**Individual* | 3 | 1 | 0.5292 | 701 |
| Sampling moment**Individual* | 3 | 270.77 | **0.0037** | 9865 |
| Residuals | 3 |  |  |  |
| Total | 15 |  |  |  |
| **Indole** | df | Pseudo-F | p | Unique perms |
| Treatment | 1 | 1 | 0.5742 | 3 |
| Sampling moment | 1 | 1 | 0.5761 | 3 |
| *Individual* | 3 | 12.211 | **0.0444** | 2750 |
| Treatment*Sampling moment | 1 | 1 | 0.4365 | 7 |
| Treatment**Individual* | 3 | 1 | 0.5258 | 703 |
| Sampling moment**Individual* | 3 | 12.211 | **0.0331** | 9845 |
| Residuals | 3 |  |  |  |
| Total | 15 |  |  |  |
| ***p*-Propylanisole** | df | Pseudo-F | p | Unique perms |
| Treatment | 1 | 0.21278 | 0.7413 | 20 |
| Sampling moment | 1 | 0.80121 | 0.5719 | 20 |
| *Individual* | 3 | 600.31 | **0.0123** | 9603 |
| Treatment*Sampling moment | 1 | 2.2982 | 0.226 | 609 |
| Treatment**Individual* | 3 | 1.6491 | 0.3448 | 9880 |
| Sampling moment**Individual* | 3 | 666.57 | **0.0017** | 9965 |
| Residuals | 3 |  |  |  |
| Total | 15 |  |  |  |
| **Methyl decanoate** | df | Pseudo-F | p | Unique perms |
| Treatment | 1 | 2.1308 | 0.2971 | 20 |
| Sampling moment | 1 | 2.1308 | 0.3054 | 20 |
| *Individual* | 3 | 1 | 0.4804 | 2088 |
| Treatment*Sampling moment | 1 | 2.1901 | 0.2383 | 585 |
| Treatment**Individual* | 3 | 0.99744 | 0.5005 | 9956 |
| Sampling moment**Individual* | 3 | 0.99744 | 0.5006 | 9964 |
| Residuals | 3 |  |  |  |
| Total | 15 |  |  |  |
| **Anisyl formate** | df | Pseudo-F | p | Unique perms |
| Treatment | 1 | 6.8794 | 0.088 | 425 |
| Sampling moment | 1 | 1.8296 | 0.2943 | 424 |
| *Individual* | 3 | 10.525 | 0.0505 | 9962 |
| Treatment*Sampling moment | 1 | 1.018 | 0.3793 | 9235 |
| Treatment**Individual* | 3 | 0.5572 | 0.6737 | 9971 |
| Sampling moment**Individual* | 3 | 5.6502 | 0.0938 | 9962 |
| Residuals | 3 |  |  |  |
| Total | 15 |  |  |  |
| **4-Methoxyphenylethyl alcohol** | df | Pseudo-F | p | Unique perms |
| Treatment | 1 | 24.83 | **0.0312** | 425 |
| Sampling moment | 1 | 7.5869 | 0.0884 | 425 |
| *Individual* | 3 | 31.001 | **0.0168** | 9953 |
| Treatment*Sampling moment | 1 | 2.5668 | 0.2098 | 9170 |
| Treatment**Individual* | 3 | 0.96485 | 0.5076 | 9968 |
| Sampling moment**Individual* | 3 | 21.64 | **0.0179** | 9966 |
| Residuals | 3 |  |  |  |
| Total | 15 |  |  |  |
| **(*E*)-Methylcinnamate** | df | Pseudo-F | p | Unique perms |
| Treatment | 1 | 1 | 0.5698 | 3 |
| Sampling moment | 1 | 1 | 0.5683 | 3 |
| *Individual* | 3 | 1 | 0.4955 | 194 |
| Treatment*Sampling moment | 1 | 1 | 0.4474 | 7 |
| Treatment**Individual* | 3 | 0.50095 | 0.7233 | 9859 |
| Sampling moment**Individual* | 3 | 0.50095 | 0.7245 | 9885 |
| Residuals | 3 |  |  |  |
| Total | 15 |  |  |  |
| **Benzyl 2-methylbutanoate** | df | Pseudo-F | p | Unique perms |
| Treatment | 1 | 1.9899 | 0.303 | 20 |
| Sampling moment | 1 | 2.9899 | 0.2452 | 20 |
| *Individual* | 3 | 2.902 | 0.1968 | 9749 |
| Treatment*Sampling moment | 1 | 1.9899 | 0.2651 | 595 |
| Treatment**Individual* | 3 | 1 | 0.5004 | 7313 |
| Sampling moment**Individual* | 3 | 2.902 | 0.2062 | 9968 |
| Residuals | 3 |  |  |  |
| Total | 15 |  |  |  |
| **Benzyl isovalerate** | df | Pseudo-F | p | Unique perms |
| Treatment | 1 | 1 | 0.5688 | 3 |
| Sampling moment | 1 | 1 | 0.5772 | 3 |
| *Individual* | 3 | 1.8962 | 0.2499 | 2766 |
| Treatment*Sampling moment | 1 | 1 | 0.4305 | 7 |
| Treatment**Individual* | 3 | 0.93811 | 0.5388 | 9868 |
| Sampling moment**Individual* | 3 | 1.0115 | 0.4738 | 9736 |
| Residuals | 3 |  |  |  |
| Total | 15 |  |  |  |
| **β-Ionone** | df | Pseudo-F | p | Unique perms |
| Treatment | 1 | 2.9323 | 0.2437 | 20 |
| Sampling moment | 1 | 9.94E-04 | 0.7974 | 20 |
| *Individual* | 3 | 0.60268 | 0.7072 | 9647 |
| Treatment*Sampling moment | 1 | 1.72E-02 | 0.8259 | 604 |
| Treatment**Individual* | 3 | 0.50861 | 0.7027 | 9870 |
| Sampling moment**Individual* | 3 | 1.2042 | 0.3825 | 9932 |
| Residuals | 3 |  |  |  |
| Total | 15 |  |  |  |
| **Benzyl tiglate** | df | Pseudo-F | p | Unique perms |
| Treatment | 1 | 0.76588 | 0.5803 | 20 |
| Sampling moment | 1 | 1.3747 | 0.4673 | 20 |
| *Individual* | 3 | 1.4236 | 0.3082 | 9739 |
| Treatment*Sampling moment | 1 | 0.76588 | 0.4571 | 597 |
| Treatment**Individual* | 3 | 1 | 0.5028 | 7693 |
| Sampling moment**Individual* | 3 | 1.4236 | 0.3793 | 9960 |
| Residuals | 3 |  |  |  |
| Total | 15 |  |  |  |
| **4-Methoxyphenylethyl acetate** | df | Pseudo-F | p | Unique perms |
| Treatment | 1 | 0.857 | 0.5709 | 20 |
| Sampling moment | 1 | 0.93265 | 0.5702 | 19 |
| *Individual* | 3 | 12.015 | **0.0469** | 9751 |
| Treatment*Sampling moment | 1 | 1.1852 | 0.3477 | 609 |
| Treatment**Individual* | 3 | 1.5485 | 0.3542 | 9963 |
| Sampling moment**Individual* | 3 | 11.045 | **0.0402** | 9974 |
| Residuals | 3 |  |  |  |
| Total | 15 |  |  |  |
| **Methyl tetradecanoate** | df | Pseudo-F | p | Unique perms |
| Treatment | 1 | 1.1134 | 0.4435 | 178 |
| Sampling moment | 1 | 1.1143 | 0.4468 | 178 |
| *Individual* | 3 | 0.99965 | 0.4278 | 9514 |
| Treatment*Sampling moment | 1 | 1.1222 | 0.3379 | 6486 |
| Treatment**Individual* | 3 | 1.0021 | 0.4872 | 9955 |
| Sampling moment**Individual* | 3 | 1.0019 | 0.4944 | 9957 |
| Residuals | 3 |  |  |  |
| Total | 15 |  |  |  |
| **Benzyl benzoate** | df | Pseudo-F | p | Unique perms |
| Treatment | 1 | 1 | 0.5745 | 3 |
| Sampling moment | 1 | 1 | 0.5728 | 3 |
| *Individual* | 3 | 4.8543 | 0.131 | 2778 |
| Treatment*Sampling moment | 1 | 1 | 0.4442 | 7 |
| Treatment**Individual* | 3 | 1 | 0.5281 | 693 |
| Sampling moment**Individual* | 3 | 4.8543 | 0.1061 | 9852 |
| Residuals | 3 |  |  |  |
| Total | 15 |  |  |  |
| **Methyl pentadecanoate** | df | Pseudo-F | p | Unique perms |
| Treatment | 1 | 0.82753 | 0.572 | 20 |
| Sampling moment | 1 | 0.82753 | 0.5628 | 20 |
| *Individual* | 3 | 1 | 0.4261 | 2078 |
| Treatment*Sampling moment | 1 | 1.1887 | 0.3446 | 606 |
| Treatment**Individual* | 3 | 1.0944 | 0.4751 | 9866 |
| Sampling moment**Individual* | 3 | 1.0944 | 0.4664 | 9876 |
| Residuals | 3 |  |  |  |
| Total | 15 |  |  |  |
| *Lantana camara* |  |  |  |  |
| **Benzaldehyde** | df | Pseudo-F | p | Unique perms |
| Treatment | 1 | 1 | 0.4922 | 2 |
| Sampling moment | 1 | 1 | 0.4968 | 2 |
| *Individual* | 1 | 1.9121 | 0.2731 | 18 |
| Treatment*Sampling moment | 1 | 1 | 0.4898 | 4 |
| Treatment**Individual* | 1 | 0.58361 | 0.5398 | 18 |
| Sampling moment**Individual* | 1 | 0.14529 | 0.6744 | 18 |
| Residuals | 1 |  |  |  |
| Total | 7 |  |  |  |
| **6-methyl-5-hepten-2-one** | df | Pseudo-F | p | Unique perms |
| Treatment | 1 | 1 | 0.5099 | 2 |
| Sampling moment | 1 | 1 | 0.5025 | 2 |
| *Individual* | 1 | 2.0423 | 0.2621 | 18 |
| Treatment*Sampling moment | 1 | 1 | 0.4965 | 4 |
| Treatment**Individual* | 1 | 0.69497 | 0.545 | 18 |
| Sampling moment**Individual* | 1 | 0.16367 | 0.6868 | 18 |
| Residuals | 1 |  |  |  |
| Total | 7 |  |  |  |
| **(*E*)-β-Ocimene** | df | Pseudo-F | p | Unique perms |
| Treatment | 1 | 1.81E-02 | 0.4939 | 3 |
| Sampling moment | 1 | 0.65645 | 0.4975 | 3 |
| *Individual* | 1 | 3.0422 | 0.1873 | 18 |
| Treatment*Sampling moment | 1 | 53.929 | 0.1204 | 18 |
| Treatment**Individual* | 1 | 54.072 | 0.1231 | 18 |
| Sampling moment**Individual* | 1 | 21.245 | 0.1207 | 18 |
| Residuals | 1 |  |  |  |
| Total | 7 |  |  |  |
| **Linalool** | df | Pseudo-F | p | Unique perms |
| Treatment | 1 | 0.15516 | 0.5029 | 3 |
| Sampling moment | 1 | 11.785 | 0.2471 | 3 |
| *Individual* | 1 | 12.434 | 0.1202 | 18 |
| Treatment*Sampling moment | 1 | 5.8579 | 0.1205 | 18 |
| Treatment**Individual* | 1 | 5.4914 | 0.1565 | 18 |
| Sampling moment**Individual* | 1 | 2.183 | 0.2571 | 18 |
| Residuals | 1 |  |  |  |
| Total | 7 |  |  |  |
| **(*E*)-4.8-dimethyl-1.3.7-nonatriene** | df | Pseudo-F | p | Unique perms |
| Treatment | 1 | 2.90E-02 | 0.5061 | 3 |
| Sampling moment | 1 | 0.41186 | 0.5038 | 3 |
| *Individual* | 1 | 76.092 | 0.123 | 18 |
| Treatment*Sampling moment | 1 | 34.449 | 0.1254 | 18 |
| Treatment**Individual* | 1 | 34.449 | 0.1155 | 18 |
| Sampling moment**Individual* | 1 | 184.75 | 0.1211 | 18 |
| Residuals | 1 |  |  |  |
| Total | 7 |  |  |  |
| **Methyl octanoate** | df | Pseudo-F | p | Unique perms |
| Treatment | 1 | 0.39914 | 0.5086 | 3 |
| Sampling moment | 1 | 2.6374 | 0.2476 | 3 |
| *Individual* | 1 | 5.7817 | 0.1571 | 18 |
| Treatment*Sampling moment | 1 | 0.17562 | 0.6589 | 18 |
| Treatment**Individual* | 1 | 0.75629 | 0.5419 | 18 |
| Sampling moment**Individual* | 1 | 5.1718 | 0.1536 | 18 |
| Residuals | 1 |  |  |  |
| Total | 7 |  |  |  |
| **Methyl salicylate** | df | Pseudo-F | p | Unique perms |
| Treatment | 1 | 1 | 0.4981 | 2 |
| Sampling moment | 1 | 1 | 0.502 | 2 |
| *Individual* | 1 | 21.266 | 0.1219 | 18 |
| Treatment*Sampling moment | 1 | 1 | 0.4997 | 4 |
| Treatment**Individual* | 1 | 1 | 0.4987 | 4 |
| Sampling moment**Individual* | 1 | 21.266 | 0.1222 | 18 |
| Residuals | 1 |  |  |  |
| Total | 7 |  |  |  |
| **Methyl decanoate** | df | Pseudo-F | p | Unique perms |
| Treatment | 1 | 5.29E-02 | 0.5053 | 3 |
| Sampling moment | 1 | 18.921 | 0.2577 | 3 |
| *Individual* | 1 | 5.29E-02 | 0.6834 | 18 |
| Treatment*Sampling moment | 1 | 5.29E-02 | 0.696 | 18 |
| Treatment**Individual* | 1 | 1 | 0.5049 | 4 |
| Sampling moment**Individual* | 1 | 5.29E-02 | 0.6884 | 18 |
| Residuals | 1 |  |  |  |
| Total | 7 |  |  |  |
| *Lippia alba* |  |  |  |  |
| **4-Methyl-3-pentene-2-ol** | df | Pseudo-F | p | Unique perms |
| Treatment | 1 | 1.6805 | 0.347 | 41 |
| Sampling moment | 1 | 1 | 0.7573 | 2 |
| *Individual* | 2 | 2.1642 | 0.424 | 9469 |
| Treatment*Sampling moment | 1 | 1 | 0.6621 | 6 |
| Treatment**Individual* | 2 | 0.78517 | 0.5964 | 3700 |
| Sampling moment**Individual* | 1 | 1.1988 | 0.3518 | 213 |
| Residuals | 1 |  |  |  |
| Total | 9 |  |  |  |
| **Hexanal** | df | Pseudo-F | p | Unique perms |
| Treatment | 1 | 1.8 | 0.3267 | 41 |
| Sampling moment | 1 | 1 | 0.751 | 2 |
| *Individual* | 2 | 17.247 | 0.1489 | 2659 |
| Treatment*Sampling moment | 1 | 1 | 0.6592 | 6 |
| Treatment**Individual* | 2 | 0.5 | 0.7321 | 21 |
| Sampling moment**Individual* | 1 | 34.494 | 0.0935 | 214 |
| Residuals | 1 |  |  |  |
| Total | 9 |  |  |  |
| **a 2-Methyl-2-vinyl-5-hydroxytetrahydrofuran (RI 928)** | df | Pseudo-F | p | Unique perms |
| Treatment | 1 | 0.202 | 0.6006 | 102 |
| Sampling moment | 1 | 1.0931 | 0.3236 | 15 |
| *Individual* | 2 | 0.18551 | 0.8623 | 9507 |
| Treatment*Sampling moment | 1 | 1.20E-02 | 0.9119 | 213 |
| Treatment**Individual* | 2 | 0.50049 | 0.7034 | 7560 |
| Sampling moment**Individual* | 1 | 0.72423 | 0.6354 | 214 |
| Residuals | 1 |  |  |  |
| Total | 9 |  |  |  |
| **a 2-Methyl-2-vinyl-5-hydroxytetrahydrofuran (RI 946)** | df | Pseudo-F | p | Unique perms |
| Treatment | 1 | 3.25E-02 | 0.8369 | 102 |
| Sampling moment | 1 | 9.69E-02 | 0.7127 | 15 |
| *Individual* | 2 | 0.90138 | 0.5996 | 9455 |
| Treatment*Sampling moment | 1 | 6.31E-02 | 0.8379 | 213 |
| Treatment**Individual* | 2 | 0.61289 | 0.6661 | 7614 |
| Sampling moment**Individual* | 1 | 0.60684 | 0.6355 | 213 |
| Residuals | 1 |  |  |  |
| Total | 9 |  |  |  |
| **Benzaldehyde** | df | Pseudo-F | p | Unique perms |
| Treatment | 1 | 0.39718 | 0.5729 | 102 |
| Sampling moment | 1 | 0.70784 | 0.6435 | 15 |
| *Individual* | 2 | 0.33851 | 0.7636 | 9516 |
| Treatment*Sampling moment | 1 | 0.4254 | 0.6445 | 214 |
| Treatment**Individual* | 2 | 0.22358 | 0.8255 | 7551 |
| Sampling moment**Individual* | 1 | 0.35892 | 0.6699 | 300 |
| Residuals | 1 |  |  |  |
| Total | 9 |  |  |  |
| **6-methyl-5-hepten-2-one** | df | Pseudo-F | p | Unique perms |
| Treatment | 1 | 0.63641 | 0.5255 | 102 |
| Sampling moment | 1 | 1.2581 | 0.3247 | 15 |
| *Individual* | 2 | 0.37997 | 0.7448 | 9485 |
| Treatment*Sampling moment | 1 | 6.92E-02 | 0.8377 | 212 |
| Treatment**Individual* | 2 | 0.28459 | 0.7917 | 7571 |
| Sampling moment**Individual* | 1 | 8.59E-02 | 0.827 | 213 |
| Residuals | 1 |  |  |  |
| Total | 9 |  |  |  |
| **β-Myrcene** | df | Pseudo-F | p | Unique perms |
| Treatment | 1 | 2.12E-02 | 0.7426 | 102 |
| Sampling moment | 1 | 5.2475 | 0.3186 | 15 |
| *Individual* | 2 | 6.85E-02 | 0.9555 | 9529 |
| Treatment*Sampling moment | 1 | 1.06E-03 | 0.9157 | 213 |
| Treatment**Individual* | 2 | 0.7196 | 0.6283 | 7598 |
| Sampling moment**Individual* | 1 | 4.88E-02 | 0.858 | 214 |
| Residuals | 1 |  |  |  |
| Total | 9 |  |  |  |
| **3-Octanol** | df | Pseudo-F | p | Unique perms |
| Treatment | 1 | 3.97E-02 | 0.8233 | 102 |
| Sampling moment | 1 | 1.1279 | 0.3174 | 15 |
| *Individual* | 2 | 0.15782 | 0.8675 | 9467 |
| Treatment*Sampling moment | 1 | 0.14555 | 0.766 | 213 |
| Treatment**Individual* | 2 | 1.8722 | 0.4212 | 7552 |
| Sampling moment**Individual* | 1 | 3.4749 | 0.303 | 214 |
| Residuals | 1 |  |  |  |
| Total | 9 |  |  |  |
| **Limonene** | df | Pseudo-F | p | Unique perms |
| Treatment | 1 | 8.91E-02 | 0.967 | 102 |
| Sampling moment | 1 | 0.8333 | 0.6351 | 15 |
| *Individual* | 2 | 0.11205 | 0.9011 | 9489 |
| Treatment*Sampling moment | 1 | 1.2 | 0.3476 | 213 |
| Treatment**Individual* | 2 | 4.1323 | 0.3217 | 7572 |
| Sampling moment**Individual* | 1 | 1.2 | 0.3481 | 214 |
| Residuals | 1 |  |  |  |
| Total | 9 |  |  |  |
| **Lavender lactone** | df | Pseudo-F | p | Unique perms |
| Treatment | 1 | 5.32E-03 | 0.9328 | 102 |
| Sampling moment | 1 | 4.1042 | 0.3075 | 15 |
| *Individual* | 2 | 1.0085 | 0.5717 | 9498 |
| Treatment*Sampling moment | 1 | 0.20767 | 0.7302 | 213 |
| Treatment**Individual* | 2 | 0.63145 | 0.6686 | 7604 |
| Sampling moment**Individual* | 1 | 0.22399 | 0.717 | 214 |
| Residuals | 1 |  |  |  |
| Total | 9 |  |  |  |
| **(*E*)-β-Ocimene** | df | Pseudo-F | p | Unique perms |
| Treatment | 1 | 0.37442 | 0.6007 | 102 |
| Sampling moment | 1 | 0.16228 | 0.6546 | 15 |
| *Individual* | 2 | 0.97396 | 0.5843 | 9529 |
| Treatment*Sampling moment | 1 | 1.2153 | 0.3503 | 214 |
| Treatment**Individual* | 2 | 0.89815 | 0.5886 | 7533 |
| Sampling moment**Individual* | 1 | 0.29447 | 0.678 | 212 |
| Residuals | 1 |  |  |  |
| Total | 9 |  |  |  |
| **(*E*)-linalool oxide furanoid** | df | Pseudo-F | p | Unique perms |
| Treatment | 1 | 6.387 | 0.1423 | 106 |
| Sampling moment | 1 | 82.642 | **0.0302** | 15 |
| *Individual* | 2 | 189.79 | 0.0542 | 9490 |
| Treatment*Sampling moment | 1 | 14.826 | 0.1462 | 213 |
| Treatment**Individual* | 2 | 6.9391 | 0.2437 | 7536 |
| Sampling moment**Individual* | 1 | 0.17344 | 0.7278 | 214 |
| Residuals | 1 |  |  |  |
| Total | 9 |  |  |  |
| **Linalool** | df | Pseudo-F | p | Unique perms |
| Treatment | 1 | 3.69E-02 | 0.8175 | 102 |
| Sampling moment | 1 | 2.2271 | 0.3263 | 15 |
| *Individual* | 2 | 0.75435 | 0.6388 | 9504 |
| Treatment*Sampling moment | 1 | 0.73175 | 0.6382 | 214 |
| Treatment**Individual* | 2 | 0.41805 | 0.732 | 7587 |
| Sampling moment**Individual* | 1 | 0.40786 | 0.6334 | 214 |
| Residuals | 1 |  |  |  |
| Total | 9 |  |  |  |
| **Hotrienol** | df | Pseudo-F | p | Unique perms |
| Treatment | 1 | 1.791 | 0.3057 | 102 |
| Sampling moment | 1 | 0.12471 | 0.639 | 15 |
| *Individual* | 2 | 0.78639 | 0.6269 | 9490 |
| Treatment*Sampling moment | 1 | 57.19 | 0.0845 | 214 |
| Treatment**Individual* | 2 | 16.624 | 0.1786 | 7545 |
| Sampling moment**Individual* | 1 | 49.38 | 0.0856 | 260 |
| Residuals | 1 |  |  |  |
| Total | 9 |  |  |  |
| **(*E*)-4.8-dimethyl-1.3.7-nonatriene** | df | Pseudo-F | p | Unique perms |
| Treatment | 1 | 1.38E-02 | 1 | 108 |
| Sampling moment | 1 | 5.5504 | 0.3103 | 15 |
| *Individual* | 2 | 0.43535 | 0.74 | 9490 |
| Treatment*Sampling moment | 1 | 0.39501 | 0.638 | 214 |
| Treatment**Individual* | 2 | 1.2767 | 0.5203 | 7586 |
| Sampling moment**Individual* | 1 | 0.13539 | 0.7645 | 213 |
| Residuals | 1 |  |  |  |
| Total | 9 |  |  |  |
| **Ocimene derivative** | df | Pseudo-F | p | Unique perms |
| Treatment | 1 | 6.8901 | 0.1797 | 102 |
| Sampling moment | 1 | 2.9979 | 0.3182 | 15 |
| *Individual* | 2 | 78.442 | 0.0939 | 9471 |
| Treatment*Sampling moment | 1 | 4.0362 | 0.267 | 371 |
| Treatment**Individual* | 2 | 1.9073 | 0.4446 | 7535 |
| Sampling moment**Individual* | 1 | 2.1931 | 0.3495 | 213 |
| Residuals | 1 |  |  |  |
| Total | 9 |  |  |  |
| ***p*-1.3.8-Menthatriene** | df | Pseudo-F | p | Unique perms |
| Treatment | 1 | 2.2721 | 0.338 | 102 |
| Sampling moment | 1 | 1.97E-02 | 0.9274 | 15 |
| *Individual* | 2 | 3.911 | 0.3394 | 9488 |
| Treatment*Sampling moment | 1 | 6.54E-02 | 0.8345 | 212 |
| Treatment**Individual* | 2 | 13.269 | 0.1856 | 7557 |
| Sampling moment**Individual* | 1 | 239.92 | 0.0739 | 214 |
| Residuals | 1 |  |  |  |
| Total | 9 |  |  |  |
| **(*E*)-linalool oxide pyranoid** | df | Pseudo-F | p | Unique perms |
| Treatment | 1 | 1.7613 | 0.2997 | 102 |
| Sampling moment | 1 | 1.5326 | 0.3204 | 15 |
| *Individual* | 2 | 895.45 | **0.0244** | 9489 |
| Treatment*Sampling moment | 1 | 0.6371 | 0.6343 | 214 |
| Treatment**Individual* | 2 | 76.189 | 0.1059 | 7606 |
| Sampling moment**Individual* | 1 | 1124.5 | 0.0727 | 214 |
| Residuals | 1 |  |  |  |
| Total | 9 |  |  |  |
| **(*Z*)-3-Hexenyl butyrate** | df | Pseudo-F | p | Unique perms |
| Treatment | 1 | 1.9245 | 0.1413 | 102 |
| Sampling moment | 1 | 3.67E-02 | 0.8567 | 15 |
| *Individual* | 2 | 0.32858 | 0.7716 | 9478 |
| Treatment*Sampling moment | 1 | 0.34286 | 0.6513 | 214 |
| Treatment**Individual* | 2 | 2.92E-03 | 0.9965 | 7596 |
| Sampling moment**Individual* | 1 | 0.25593 | 0.6994 | 232 |
| Residuals | 1 |  |  |  |
| Total | 9 |  |  |  |
| **α-Terpineol** | df | Pseudo-F | p | Unique perms |
| Treatment | 1 | 0.17884 | 0.6418 | 102 |
| Sampling moment | 1 | 0.75335 | 0.6441 | 15 |
| *Individual* | 2 | 1.1197 | 0.5619 | 9442 |
| Treatment*Sampling moment | 1 | 0.24415 | 0.7105 | 228 |
| Treatment**Individual* | 2 | 2.1699 | 0.4342 | 7602 |
| Sampling moment**Individual* | 1 | 11.03 | 0.1614 | 214 |
| Residuals | 1 |  |  |  |
| Total | 9 |  |  |  |
| **Methyl salicylate** | df | Pseudo-F | p | Unique perms |
| Treatment | 1 | 3.90E-02 | 0.7948 | 102 |
| Sampling moment | 1 | 0.22591 | 0.6389 | 15 |
| *Individual* | 2 | 1416.1 | **0.0253** | 9471 |
| Treatment*Sampling moment | 1 | 94.525 | 0.0783 | 213 |
| Treatment**Individual* | 2 | 243.88 | 0.0518 | 7592 |
| Sampling moment**Individual* | 1 | 793.21 | 0.0738 | 214 |
| Residuals | 1 |  |  |  |
| Total | 9 |  |  |  |
| **Neral** | df | Pseudo-F | p | Unique perms |
| Treatment | 1 | 1.1611 | 0.4513 | 102 |
| Sampling moment | 1 | 0.18127 | 0.6419 | 15 |
| *Individual* | 2 | 2538.9 | **0.0194** | 9508 |
| Treatment*Sampling moment | 1 | 30.918 | 0.1067 | 213 |
| Treatment**Individual* | 2 | 561.59 | **0.0225** | 7565 |
| Sampling moment**Individual* | 1 | 848.34 | 0.0704 | 214 |
| Residuals | 1 |  |  |  |
| Total | 9 |  |  |  |
| **Geranial** | df | Pseudo-F | p | Unique perms |
| Treatment | 1 | 5.59E-02 | 0.9669 | 102 |
| Sampling moment | 1 | 2.7242 | 0.3233 | 15 |
| *Individual* | 2 | 3.7362 | 0.3491 | 9465 |
| Treatment*Sampling moment | 1 | 4.57E-02 | 0.8656 | 213 |
| Treatment**Individual* | 2 | 0.75538 | 0.6247 | 7589 |
| Sampling moment**Individual* | 1 | 4.4824 | 0.2702 | 214 |
| Residuals | 1 |  |  |  |
| Total | 9 |  |  |  |
| **β-Bourbonene** | df | Pseudo-F | p | Unique perms |
| Treatment | 1 | 5.91E-02 | 0.8778 | 102 |
| Sampling moment | 1 | 2.1422 | 0.3247 | 15 |
| *Individual* | 2 | 2.9425 | 0.3751 | 9513 |
| Treatment*Sampling moment | 1 | 0.87356 | 0.6335 | 213 |
| Treatment**Individual* | 2 | 1.4978 | 0.4875 | 7595 |
| Sampling moment**Individual* | 1 | 2.1258 | 0.347 | 213 |
| Residuals | 1 |  |  |  |
| Total | 9 |  |  |  |
| **β-Caryophyllene** | df | Pseudo-F | p | Unique perms |
| Treatment | 1 | 0.27253 | 0.5872 | 102 |
| Sampling moment | 1 | 1.0397 | 0.3223 | 16 |
| *Individual* | 2 | 4.6666 | 0.3016 | 9472 |
| Treatment*Sampling moment | 1 | 7.40E-02 | 0.8281 | 212 |
| Treatment**Individual* | 2 | 2.3967 | 0.4138 | 7601 |
| Sampling moment**Individual* | 1 | 9.276 | 0.1649 | 280 |
| Residuals | 1 |  |  |  |
| Total | 9 |  |  |  |
| **(*E*)-β-Farnesene** | df | Pseudo-F | p | Unique perms |
| Treatment | 1 | 5.77E-03 | 0.8349 | 102 |
| Sampling moment | 1 | 3.7315 | 0.3291 | 15 |
| *Individual* | 2 | 0.33491 | 0.7689 | 9500 |
| Treatment*Sampling moment | 1 | 1.1722 | 0.3467 | 213 |
| Treatment**Individual* | 2 | 1.2481 | 0.5146 | 2014 |
| Sampling moment**Individual* | 1 | 0.50467 | 0.6273 | 214 |
| Residuals | 1 |  |  |  |
| Total | 9 |  |  |  |
| **Germacrene D** | df | Pseudo-F | p | Unique perms |
| Treatment | 1 | 4.0449 | 0.2391 | 102 |
| Sampling moment | 1 | 1.0339 | 0.3212 | 16 |
| *Individual* | 2 | 2.7481 | 0.4025 | 9474 |
| Treatment*Sampling moment | 1 | 0.4284 | 0.646 | 213 |
| Treatment**Individual* | 2 | 3.5312 | 0.3309 | 7591 |
| Sampling moment**Individual* | 1 | 2.8236 | 0.3184 | 224 |
| Residuals | 1 |  |  |  |
| Total | 9 |  |  |  |
| **α-Amorphene** | df | Pseudo-F | p | Unique perms |
| Treatment | 1 | 3.45E-02 | 1 | 102 |
| Sampling moment | 1 | 0.68134 | 0.6512 | 15 |
| *Individual* | 2 | 0.14586 | 0.878 | 9474 |
| Treatment*Sampling moment | 1 | 2.26E-02 | 0.8922 | 214 |
| Treatment**Individual* | 2 | 0.34615 | 0.7617 | 7551 |
| Sampling moment**Individual* | 1 | 1.34 | 0.3497 | 213 |
| Residuals | 1 |  |  |  |
| Total | 9 |  |  |  |
| **Kessane** | df | Pseudo-F | p | Unique perms |
| Treatment | 1 | 0.729 | 0.449 | 112 |
| Sampling moment | 1 | 2.1702 | 0.33 | 15 |
| *Individual* | 2 | 0.38602 | 0.7393 | 9509 |
| Treatment*Sampling moment | 1 | 4.03E-02 | 0.8613 | 300 |
| Treatment**Individual* | 2 | 1.85E-02 | 0.9808 | 7622 |
| Sampling moment**Individual* | 1 | 0.12565 | 0.7794 | 212 |
| Residuals | 1 |  |  |  |
| Total | 9 |  |  |  |
| *Tocoyena formosa* |  |  |  |  |
| **2-Methylbutanenitrile** | df | Pseudo-F | p | Unique perms |
| Treatment | 1 | 3.0125 | 0.1895 | 207 |
| Sampling moment | 1 | 8.8469 | 0.0593 | 201 |
| *Individual* | 3 | 15.721 | **0.0325** | 9963 |
| Treatment*Sampling moment | 1 | 2.5616 | 0.2139 | 7643 |
| Treatment**Individual* | 3 | 0.65078 | 0.6314 | 9963 |
| Sampling moment**Individual* | 3 | 1.3825 | 0.3981 | 9970 |
| Residuals | 3 |  |  |  |
| Total | 15 |  |  |  |
| **Isobutylaldoxime** | df | Pseudo-F | p | Unique perms |
| Treatment | 1 | 1 | 0.5778 | 3 |
| Sampling moment | 1 | 1 | 0.5702 | 3 |
| *Individual* | 3 | 10.884 | 0.0525 | 2784 |
| Treatment*Sampling moment | 1 | 1 | 0.4501 | 7 |
| Treatment**Individual* | 3 | 1 | 0.5271 | 706 |
| Sampling moment**Individual* | 3 | 10.884 | **0.0395** | 9862 |
| Residuals | 3 |  |  |  |
| Total | 15 |  |  |  |
| **Hexanal** | df | Pseudo-F | p | Unique perms |
| Treatment | 1 | 1 | 0.5736 | 3 |
| Sampling moment | 1 | 1 | 0.5657 | 3 |
| *Individual* | 3 | 167.53 | **0.0365** | 2765 |
| Treatment*Sampling moment | 1 | 1 | 0.4529 | 7 |
| Treatment**Individual* | 3 | 1 | 0.5295 | 686 |
| Sampling moment**Individual* | 3 | 167.53 | **0.0039** | 9869 |
| Residuals | 3 |  |  |  |
| Total | 15 |  |  |  |
| ***syn*-3-Methylbutylaldoxime** | df | Pseudo-F | p | Unique perms |
| Treatment | 1 | 1.1864 | 0.3707 | 425 |
| Sampling moment | 1 | 0.24614 | 0.5882 | 425 |
| *Individual* | 3 | 2.1445 | 0.2764 | 9952 |
| Treatment*Sampling moment | 1 | 10.403 | 0.052 | 9225 |
| Treatment**Individual* | 3 | 3.3871 | 0.1822 | 9964 |
| Sampling moment**Individual* | 3 | 5.8382 | 0.0953 | 9965 |
| Residuals | 3 |  |  |  |
| Total | 15 |  |  |  |
| ***syn*-2-Methylbutylaldoxime** | df | Pseudo-F | p | Unique perms |
| Treatment | 1 | 2.229 | 0.2552 | 425 |
| Sampling moment | 1 | 173.3 | **0.0295** | 423 |
| *Individual* | 3 | 15.712 | **0.0412** | 9957 |
| Treatment*Sampling moment | 1 | 2.3753 | 0.2293 | 9189 |
| Treatment**Individual* | 3 | 0.49949 | 0.7219 | 9973 |
| Sampling moment**Individual* | 3 | 6.44E-03 | 0.9986 | 9975 |
| Residuals | 3 |  |  |  |
| Total | 15 |  |  |  |
| ***anti*-2-Methylbutylaldoxime** | df | Pseudo-F | p | Unique perms |
| Treatment | 1 | 2.229 | 0.2559 | 425 |
| Sampling moment | 1 | 173.3 | **0.0275** | 423 |
| *Individual* | 3 | 15.712 | **0.0399** | 9952 |
| Treatment*Sampling moment | 1 | 2.3753 | 0.2342 | 9152 |
| Treatment**Individual* | 3 | 0.49949 | 0.7187 | 9958 |
| Sampling moment**Individual* | 3 | 6.44E-03 | 0.998 | 9977 |
| Residuals | 3 |  |  |  |
| Total | 15 |  |  |  |
| **1-Hexanol** | df | Pseudo-F | p | Unique perms |
| Treatment | 1 | 5.4869 | 0.1062 | 425 |
| Sampling moment | 1 | 15.971 | **0.0345** | 425 |
| *Individual* | 3 | 1.3886 | 0.4036 | 9964 |
| Treatment*Sampling moment | 1 | 8.4949 | 0.0599 | 9203 |
| Treatment**Individual* | 3 | 0.97763 | 0.5106 | 9966 |
| Sampling moment**Individual* | 3 | 2.3438 | 0.2525 | 9968 |
| Residuals | 3 |  |  |  |
| Total | 15 |  |  |  |
| **Benzaldehyde** | df | Pseudo-F | p | Unique perms |
| Treatment | 1 | 0.23631 | 0.6224 | 421 |
| Sampling moment | 1 | 9.65E-04 | 0.9177 | 425 |
| *Individual* | 3 | 75.995 | **0.0045** | 9961 |
| Treatment*Sampling moment | 1 | 3.21 | 0.1677 | 9226 |
| Treatment**Individual* | 3 | 11.773 | **0.0389** | 9965 |
| Sampling moment**Individual* | 3 | 4.2002 | 0.1404 | 9965 |
| Residuals | 3 |  |  |  |
| Total | 15 |  |  |  |
| **1-Octen-3-ol** | df | Pseudo-F | p | Unique perms |
| Treatment | 1 | 2.0251 | 0.2654 | 207 |
| Sampling moment | 1 | 3.78E-02 | 0.8747 | 207 |
| *Individual* | 3 | 2.8667 | 0.2004 | 9947 |
| Treatment*Sampling moment | 1 | 2.58E-02 | 0.8172 | 7602 |
| Treatment**Individual* | 3 | 0.60214 | 0.6515 | 9960 |
| Sampling moment**Individual* | 3 | 9.1547 | 0.0529 | 9963 |
| Residuals | 3 |  |  |  |
| Total | 15 |  |  |  |
| **6-methyl-5-hepten-2-one** | df | Pseudo-F | p | Unique perms |
| Treatment | 1 | 4.9611 | 0.1326 | 409 |
| Sampling moment | 1 | 25.124 | **0.0332** | 424 |
| *Individual* | 3 | 6.9391 | 0.0769 | 9962 |
| Treatment*Sampling moment | 1 | 10.437 | **0.0468** | 9172 |
| Treatment**Individual* | 3 | 3.4395 | 0.1708 | 9976 |
| Sampling moment**Individual* | 3 | 1.6921 | 0.3421 | 9968 |
| Residuals | 3 |  |  |  |
| Total | 15 |  |  |  |
| **β-Myrcene** | df | Pseudo-F | p | Unique perms |
| Treatment | 1 | 0.4676 | 0.5712 | 20 |
| Sampling moment | 1 | 2.955 | 0.239 | 20 |
| *Individual* | 3 | 48.817 | **0.0161** | 9737 |
| Treatment*Sampling moment | 1 | 0.4676 | 0.5624 | 612 |
| Treatment**Individual* | 3 | 1 | 0.5087 | 7635 |
| Sampling moment**Individual* | 3 | 48.817 | **0.0205** | 9965 |
| Residuals | 3 |  |  |  |
| Total | 15 |  |  |  |
| **Benzyl alcohol** | df | Pseudo-F | p | Unique perms |
| Treatment | 1 | 0.26141 | 0.6825 | 420 |
| Sampling moment | 1 | 5.4087 | 0.1221 | 425 |
| *Individual* | 3 | 24.5 | **0.0282** | 9955 |
| Treatment*Sampling moment | 1 | 6.61E-03 | 0.8475 | 9043 |
| Treatment**Individual* | 3 | 2.1493 | 0.2694 | 9962 |
| Sampling moment**Individual* | 3 | 1.7255 | 0.3441 | 9967 |
| Residuals | 3 |  |  |  |
| Total | 15 |  |  |  |
| **(*Z*)-β-Ocimene** | df | Pseudo-F | p | Unique perms |
| Treatment | 1 | 1 | 0.5774 | 3 |
| Sampling moment | 1 | 1 | 0.5732 | 3 |
| *Individual* | 3 | 552.35 | **0.0371** | 2666 |
| Treatment*Sampling moment | 1 | 1 | 0.4405 | 7 |
| Treatment**Individual* | 3 | 1 | 0.5297 | 694 |
| Sampling moment**Individual* | 3 | 552.35 | **0.0046** | 9884 |
| Residuals | 3 |  |  |  |
| Total | 15 |  |  |  |
| **(*E*)-β-Ocimene** | df | Pseudo-F | p | Unique perms |
| Treatment | 1 | 3.6482 | 0.1658 | 425 |
| Sampling moment | 1 | 0.16143 | 0.7421 | 425 |
| *Individual* | 3 | 36.051 | **0.0123** | 9949 |
| Treatment*Sampling moment | 1 | 0.22019 | 0.6683 | 9118 |
| Treatment**Individual* | 3 | 3.3359 | 0.1818 | 9968 |
| Sampling moment**Individual* | 3 | 9.0594 | 0.0532 | 9966 |
| Residuals | 3 |  |  |  |
| Total | 15 |  |  |  |
| **(*Z*)-linalool oxide furanoid** | df | Pseudo-F | p | Unique perms |
| Treatment | 1 | 2.0466 | 0.2449 | 434 |
| Sampling moment | 1 | 1.4555 | 0.3983 | 402 |
| *Individual* | 3 | 7.4094 | 0.0658 | 9954 |
| Treatment*Sampling moment | 1 | 1.8257 | 0.2817 | 8988 |
| Treatment**Individual* | 3 | 0.97444 | 0.5006 | 9958 |
| Sampling moment**Individual* | 3 | 4.2635 | 0.1372 | 9966 |
| Residuals | 3 |  |  |  |
| Total | 15 |  |  |  |
| **(*E*)-linalool oxide furanoid** | df | Pseudo-F | p | Unique perms |
| Treatment | 1 | 2.34E-02 | 0.871 | 425 |
| Sampling moment | 1 | 3.0826 | 0.1689 | 425 |
| *Individual* | 3 | 3.0237 | 0.1977 | 9957 |
| Treatment*Sampling moment | 1 | 0.44973 | 0.5492 | 9226 |
| Treatment**Individual* | 3 | 1.5658 | 0.3593 | 9988 |
| Sampling moment**Individual* | 3 | 1.3866 | 0.3651 | 9970 |
| Residuals | 3 |  |  |  |
| Total | 15 |  |  |  |
| **Methyl benzoate** | df | Pseudo-F | p | Unique perms |
| Treatment | 1 | 5.216 | 0.1206 | 425 |
| Sampling moment | 1 | 100.02 | **0.0272** | 425 |
| *Individual* | 3 | 2.9888 | 0.2058 | 9962 |
| Treatment*Sampling moment | 1 | 1.0776 | 0.3813 | 9184 |
| Treatment**Individual* | 3 | 1.6621 | 0.3502 | 9970 |
| Sampling moment**Individual* | 3 | 6.9559 | 0.08 | 9966 |
| Residuals | 3 |  |  |  |
| Total | 15 |  |  |  |
| **2.2.6-Trimethy-6-vinyldihydro-2H-pyran-3(4H)-one** | df | Pseudo-F | p | Unique perms |
| Treatment | 1 | 1.42E-03 | 0.8696 | 422 |
| Sampling moment | 1 | 35.525 | **0.0301** | 425 |
| *Individual* | 3 | 10.629 | **0.0449** | 9963 |
| Treatment*Sampling moment | 1 | 1.398 | 0.3193 | 9137 |
| Treatment**Individual* | 3 | 1.6131 | 0.3262 | 9967 |
| Sampling moment**Individual* | 3 | 5.5126 | 0.0957 | 9963 |
| Residuals | 3 |  |  |  |
| Total | 15 |  |  |  |
| **2-Phenylethanol** | df | Pseudo-F | p | Unique perms |
| Treatment | 1 | 4.4517 | 0.1365 | 415 |
| Sampling moment | 1 | 5.5024 | 0.1217 | 421 |
| *Individual* | 3 | 22.268 | **0.018** | 9963 |
| Treatment*Sampling moment | 1 | 0.4297 | 0.5561 | 9163 |
| Treatment**Individual* | 3 | 3.5666 | 0.162 | 9966 |
| Sampling moment**Individual* | 3 | 26.49 | **0.0159** | 9969 |
| Residuals | 3 |  |  |  |
| Total | 15 |  |  |  |
| ***allo*-Ocimene** | df | Pseudo-F | p | Unique perms |
| Treatment | 1 | 26.959 | **0.0265** | 424 |
| Sampling moment | 1 | 50.317 | **0.0347** | 429 |
| *Individual* | 3 | 2.0854 | 0.2722 | 9959 |
| Treatment*Sampling moment | 1 | 9.64E-02 | 0.7761 | 9226 |
| Treatment**Individual* | 3 | 4.24E-02 | 0.9853 | 9970 |
| Sampling moment**Individual* | 3 | 0.14685 | 0.921 | 9959 |
| Residuals | 3 |  |  |  |
| Total | 15 |  |  |  |
| **Methyl nicotinate** | df | Pseudo-F | p | Unique perms |
| Treatment | 1 | 5.2241 | 0.125 | 425 |
| Sampling moment | 1 | 31.587 | **0.0295** | 425 |
| *Individual* | 3 | 3.7323 | 0.1556 | 9955 |
| Treatment*Sampling moment | 1 | 2.2398 | 0.2444 | 9142 |
| Treatment**Individual* | 3 | 1.2672 | 0.4257 | 9969 |
| Sampling moment**Individual* | 3 | 2.7433 | 0.2193 | 9959 |
| Residuals | 3 |  |  |  |
| Total | 15 |  |  |  |
| ***p*-Vinylanisole** | df | Pseudo-F | p | Unique perms |
| Treatment | 1 | 4.9241 | 0.1275 | 207 |
| Sampling moment | 1 | 4.8361 | 0.1278 | 207 |
| *Individual* | 3 | 1.1031 | 0.475 | 9957 |
| Treatment*Sampling moment | 1 | 4.9241 | 0.1163 | 7656 |
| Treatment**Individual* | 3 | 1 | 0.502 | 9715 |
| Sampling moment**Individual* | 3 | 1.1031 | 0.4581 | 9965 |
| Residuals | 3 |  |  |  |
| Total | 15 |  |  |  |
| **Benzyl acetate** | df | Pseudo-F | p | Unique perms |
| Treatment | 1 | 5.449 | 0.1278 | 425 |
| Sampling moment | 1 | 1.0398 | 0.3749 | 425 |
| *Individual* | 3 | 8.5806 | 0.062 | 9963 |
| Treatment*Sampling moment | 1 | 0.3459 | 0.6065 | 8986 |
| Treatment**Individual* | 3 | 0.77882 | 0.571 | 9966 |
| Sampling moment**Individual* | 3 | 4.4134 | 0.1271 | 9975 |
| Residuals | 3 |  |  |  |
| Total | 15 |  |  |  |
| **(*Z*)-linalool oxide pyranoid** | df | Pseudo-F | p | Unique perms |
| Treatment | 1 | 0.68008 | 0.4701 | 425 |
| Sampling moment | 1 | 19.718 | **0.0487** | 425 |
| *Individual* | 3 | 8.0322 | 0.0626 | 9948 |
| Treatment*Sampling moment | 1 | 1.3884 | 0.3129 | 9182 |
| Treatment**Individual* | 3 | 0.39248 | 0.7632 | 9969 |
| Sampling moment**Individual* | 3 | 1.7913 | 0.3179 | 9966 |
| Residuals | 3 |  |  |  |
| Total | 15 |  |  |  |
| **(*E*)-linalool oxide pyranoid** | df | Pseudo-F | p | Unique perms |
| Treatment | 1 | 6.2985 | 0.086 | 419 |
| Sampling moment | 1 | 245.87 | **0.0298** | 421 |
| *Individual* | 3 | 2.9736 | 0.2075 | 9958 |
| Treatment*Sampling moment | 1 | 1.1336 | 0.3616 | 9300 |
| Treatment**Individual* | 3 | 0.57121 | 0.6639 | 9974 |
| Sampling moment**Individual* | 3 | 1.4682 | 0.3866 | 9974 |
| Residuals | 3 |  |  |  |
| Total | 15 |  |  |  |
| **Methyl salicylate** | df | Pseudo-F | p | Unique perms |
| Treatment | 1 | 0.79565 | 0.5643 | 402 |
| Sampling moment | 1 | 2.2513 | 0.2474 | 424 |
| *Individual* | 3 | 18.266 | **0.0397** | 9958 |
| Treatment*Sampling moment | 1 | 0.86608 | 0.4341 | 8172 |
| Treatment**Individual* | 3 | 0.33015 | 0.8175 | 9967 |
| Sampling moment**Individual* | 3 | 1.03E-02 | 0.9954 | 9974 |
| Residuals | 3 |  |  |  |
| Total | 15 |  |  |  |
| ***p*-Anisaldehyde** | df | Pseudo-F | p | Unique perms |
| Treatment | 1 | 0.87442 | 0.4395 | 207 |
| Sampling moment | 1 | 3.6873 | 0.1692 | 207 |
| *Individual* | 3 | 2.1332 | 0.2739 | 9923 |
| Treatment*Sampling moment | 1 | 0.87442 | 0.4188 | 7603 |
| Treatment**Individual* | 3 | 1 | 0.5038 | 9704 |
| Sampling moment**Individual* | 3 | 2.1332 | 0.2791 | 9961 |
| Residuals | 3 |  |  |  |
| Total | 15 |  |  |  |
| **(3*E*.7*E*)-4.8.12-Trimethyltrideca-1.3.7.11-tetraene** | df | Pseudo-F | p | Unique perms |
| Treatment | 1 | 1.3764 | 0.3478 | 425 |
| Sampling moment | 1 | 1.0629 | 0.3735 | 425 |
| *Individual* | 3 | 4.9471 | 0.1214 | 9952 |
| Treatment*Sampling moment | 1 | 2.3565 | 0.2278 | 9073 |
| Treatment**Individual* | 3 | 0.57054 | 0.663 | 9964 |
| Sampling moment**Individual* | 3 | 1.3524 | 0.4071 | 9969 |
| Residuals | 3 |  |  |  |
| Total | 15 |  |  |  |
| **Benzyl benzoate** | df | Pseudo-F | p | Unique perms |
| Treatment | 1 | 2.3056 | 0.243 | 20 |
| Sampling moment | 1 | 1.4091 | 0.4595 | 20 |
| *Individual* | 3 | 130.34 | **0.0206** | 9681 |
| Treatment*Sampling moment | 1 | 2.4115 | 0.2275 | 598 |
| Treatment**Individual* | 3 | 5.0852 | 0.1134 | 9953 |
| Sampling moment**Individual* | 3 | 78.674 | **0.0025** | 9970 |
| Residuals | 3 |  |  |  |
| Total | 15 |  |  |  |
| *Zeyheria montana* |  |  |  |  |
| **Ethyl 2-methylacrylate** | df | Pseudo-F | p | Unique perms |
| Treatment | 1 | 0.74752 | 0.5901 | 38 |
| Sampling moment | 1 | 0.35164 | 0.5855 | 38 |
| *Individual* | 5 | 6.4759 | **0.0467** | 9947 |
| Treatment*Sampling moment | 1 | 1.2808 | 0.3131 | 1363 |
| Treatment**Individual* | 4 | 1.1123 | 0.4503 | 9955 |
| Sampling moment**Individual* | 4 | 1.3307 | 0.3775 | 9964 |
| Residuals | 4 |  |  |  |
| Total | 20 |  |  |  |
| **1-Hexanol** | df | Pseudo-F | p | Unique perms |
| Treatment | 1 | 1 | 0.6298 | 3 |
| Sampling moment | 1 | 1 | 0.6393 | 3 |
| *Individual* | 5 | 1.1179 | 0.3571 | 9930 |
| Treatment*Sampling moment | 1 | 1 | 0.4687 | 12 |
| Treatment**Individual* | 4 | 1.0387 | 0.4746 | 9969 |
| Sampling moment**Individual* | 4 | 0.70073 | 0.6736 | 9964 |
| Residuals | 4 |  |  |  |
| Total | 20 |  |  |  |
| **2-Methylbutyl acetate** | df | Pseudo-F | p | Unique perms |
| Treatment | 1 | 2.1419 | 0.237 | 572 |
| Sampling moment | 1 | 2.6493 | 0.1969 | 584 |
| *Individual* | 5 | 1.4776 | 0.3016 | 9960 |
| Treatment*Sampling moment | 1 | 1.8123 | 0.256 | 8607 |
| Treatment**Individual* | 4 | 0.4961 | 0.7588 | 9960 |
| Sampling moment**Individual* | 4 | 0.16267 | 0.9479 | 9961 |
| Residuals | 4 |  |  |  |
| Total | 20 |  |  |  |
| **Benzaldehyde** | df | Pseudo-F | p | Unique perms |
| Treatment | 1 | 4.1416 | 0.1238 | 644 |
| Sampling moment | 1 | 3.1644 | 0.1492 | 644 |
| *Individual* | 5 | 4.5759 | 0.0862 | 9957 |
| Treatment*Sampling moment | 1 | 4.3053 | 0.11 | 9301 |
| Treatment**Individual* | 4 | 2.0665 | 0.2597 | 9966 |
| Sampling moment**Individual* | 4 | 5.5538 | 0.0649 | 9966 |
| Residuals | 4 |  |  |  |
| Total | 20 |  |  |  |
| **1-Octen-3-ol** | df | Pseudo-F | p | Unique perms |
| Treatment | 1 | 2.0214 | 0.2715 | 5983 |
| Sampling moment | 1 | 5.0983 | 0.0927 | 5994 |
| *Individual* | 5 | 7.4871 | **0.0464** | 9950 |
| Treatment*Sampling moment | 1 | 4.1346 | 0.1122 | 9782 |
| Treatment**Individual* | 4 | 9.5851 | **0.0233** | 9971 |
| Sampling moment**Individual* | 4 | 0.89409 | 0.5431 | 9960 |
| Residuals | 4 |  |  |  |
| Total | 20 |  |  |  |
| **3-Octanone** | df | Pseudo-F | p | Unique perms |
| Treatment | 1 | 1 | 0.6328 | 3 |
| Sampling moment | 1 | 1 | 0.6436 | 3 |
| *Individual* | 5 | 6.5347 | 0.0639 | 9966 |
| Treatment*Sampling moment | 1 | 1 | 0.462 | 12 |
| Treatment**Individual* | 4 | 8.0283 | **0.0303** | 9955 |
| Sampling moment**Individual* | 4 | 1 | 0.5075 | 3058 |
| Residuals | 4 |  |  |  |
| Total | 20 |  |  |  |
| **(*Z*)-3-Hexenyl acetate** | df | Pseudo-F | p | Unique perms |
| Treatment | 1 | 1.3482 | 0.4331 | 38 |
| Sampling moment | 1 | 2.55E-02 | 0.8656 | 38 |
| *Individual* | 5 | 13.089 | **0.0232** | 9953 |
| Treatment*Sampling moment | 1 | 2.55E-02 | 0.8698 | 1352 |
| Treatment**Individual* | 4 | 16.361 | **0.0106** | 9972 |
| Sampling moment**Individual* | 4 | 1 | 0.5089 | 9815 |
| Residuals | 4 |  |  |  |
| Total | 20 |  |  |  |
| **Benzyl alcohol** | df | Pseudo-F | p | Unique perms |
| Treatment | 1 | 6.7591 | 0.063 | 6090 |
| Sampling moment | 1 | 0.19742 | 0.7099 | 5943 |
| *Individual* | 5 | 23.657 | **0.0047** | 9965 |
| Treatment*Sampling moment | 1 | 0.98742 | 0.381 | 9829 |
| Treatment**Individual* | 4 | 1.7354 | 0.3107 | 9965 |
| Sampling moment**Individual* | 4 | 9.5879 | **0.0234** | 9972 |
| Residuals | 4 |  |  |  |
| Total | 20 |  |  |  |
| **Lavender lactone** | df | Pseudo-F | p | Unique perms |
| Treatment | 1 | 0.1063 | 0.7746 | 643 |
| Sampling moment | 1 | 0.42458 | 0.5262 | 642 |
| *Individual* | 5 | 0.36 | 0.8514 | 9965 |
| Treatment*Sampling moment | 1 | 0.38781 | 0.567 | 9271 |
| Treatment**Individual* | 4 | 1.0686 | 0.4783 | 9960 |
| Sampling moment**Individual* | 4 | 0.99169 | 0.502 | 9967 |
| Residuals | 4 |  |  |  |
| Total | 20 |  |  |  |
| **(*Z*)-linalool oxide furanoid** | df | Pseudo-F | p | Unique perms |
| Treatment | 1 | 2.6795 | 0.1871 | 590 |
| Sampling moment | 1 | 1.3687 | 0.4357 | 501 |
| *Individual* | 5 | 4.3594 | 0.1193 | 9962 |
| Treatment*Sampling moment | 1 | 0.76052 | 0.4586 | 7846 |
| Treatment**Individual* | 4 | 1.54E-02 | 0.9997 | 9964 |
| Sampling moment**Individual* | 4 | 0.47184 | 0.7969 | 9954 |
| Residuals | 4 |  |  |  |
| Total | 20 |  |  |  |
| **(*E*)-linalool oxide furanoid** | df | Pseudo-F | p | Unique perms |
| Treatment | 1 | 1.06E-02 | 0.8628 | 38 |
| Sampling moment | 1 | 1.06E-02 | 0.8691 | 38 |
| *Individual* | 5 | 0.8 | 0.5913 | 9437 |
| Treatment*Sampling moment | 1 | 2.6374 | 0.1796 | 1352 |
| Treatment**Individual* | 4 | 1.655 | 0.316 | 9959 |
| Sampling moment**Individual* | 4 | 1.655 | 0.3171 | 9963 |
| Residuals | 4 |  |  |  |
| Total | 20 |  |  |  |
| **Linalool** | df | Pseudo-F | p | Unique perms |
| Treatment | 1 | 2.6569 | 0.1997 | 38 |
| Sampling moment | 1 | 0.74056 | 0.5889 | 38 |
| *Individual* | 5 | 1.521 | 0.3547 | 9955 |
| Treatment*Sampling moment | 1 | 0.74056 | 0.4667 | 1347 |
| Treatment**Individual* | 4 | 1.5529 | 0.3428 | 9962 |
| Sampling moment**Individual* | 4 | 1 | 0.5042 | 9826 |
| Residuals | 4 |  |  |  |
| Total | 20 |  |  |  |
| **Methyl benzoate** | df | Pseudo-F | p | Unique perms |
| Treatment | 1 | 1 | 0.6405 | 3 |
| Sampling moment | 1 | 1 | 0.6336 | 3 |
| *Individual* | 5 | 1.5298 | 0.2705 | 9955 |
| Treatment*Sampling moment | 1 | 1 | 0.4675 | 12 |
| Treatment**Individual* | 4 | 1.9062 | 0.2307 | 9973 |
| Sampling moment**Individual* | 4 | 1 | 0.5201 | 3057 |
| Residuals | 4 |  |  |  |
| Total | 20 |  |  |  |
| **2-Ethylhexoic acid** | df | Pseudo-F | p | Unique perms |
| Treatment | 1 | 1 | 0.6369 | 3 |
| Sampling moment | 1 | 1 | 0.6399 | 3 |
| *Individual* | 5 | 120.38 | **0.0099** | 9959 |
| Treatment*Sampling moment | 1 | 1 | 0.4677 | 12 |
| Treatment**Individual* | 4 | 145.38 | **0.0002** | 9966 |
| Sampling moment**Individual* | 4 | 1 | 0.5172 | 3043 |
| Residuals | 4 |  |  |  |
| Total | 20 |  |  |  |
| **(*E*)-4.8-dimethyl-1.3.7-nonatriene** | df | Pseudo-F | p | Unique perms |
| Treatment | 1 | 1 | 0.6435 | 3 |
| Sampling moment | 1 | 1 | 0.6417 | 3 |
| *Individual* | 5 | 0.93768 | 0.4344 | 9955 |
| Treatment*Sampling moment | 1 | 1 | 0.469 | 12 |
| Treatment**Individual* | 4 | 1.005 | 0.4801 | 9953 |
| Sampling moment**Individual* | 4 | 1 | 0.5212 | 3000 |
| Residuals | 4 |  |  |  |
| Total | 20 |  |  |  |
| **Benzyl acetate** | df | Pseudo-F | p | Unique perms |
| Treatment | 1 | 0.98109 | 0.5829 | 33 |
| Sampling moment | 1 | 0.98105 | 0.5901 | 33 |
| *Individual* | 5 | 0.80378 | 0.5139 | 9949 |
| Treatment*Sampling moment | 1 | 1.0191 | 0.3195 | 1107 |
| Treatment**Individual* | 4 | 1.0124 | 0.491 | 9957 |
| Sampling moment**Individual* | 4 | 1.0076 | 0.4949 | 9970 |
| Residuals | 4 |  |  |  |
| Total | 20 |  |  |  |
| **Linalyl acetate** | df | Pseudo-F | p | Unique perms |
| Treatment | 1 | 0.47499 | 0.5883 | 38 |
| Sampling moment | 1 | 0.47402 | 0.59 | 38 |
| *Individual* | 5 | 0.8 | 0.5377 | 9900 |
| Treatment*Sampling moment | 1 | 1.6646 | 0.2711 | 1324 |
| Treatment**Individual* | 4 | 1.2658 | 0.3999 | 9969 |
| Sampling moment**Individual* | 4 | 1.2665 | 0.388 | 9960 |
| Residuals | 4 |  |  |  |
| Total | 20 |  |  |  |
| **Tridecane** | df | Pseudo-F | p | Unique perms |
| Treatment | 1 | 3.7026 | 0.1393 | 643 |
| Sampling moment | 1 | 2.2797 | 0.2181 | 642 |
| *Individual* | 5 | 1.2516 | 0.4335 | 9965 |
| Treatment*Sampling moment | 1 | 2.2797 | 0.2102 | 9220 |
| Treatment**Individual* | 4 | 1.5645 | 0.342 | 9963 |
| Sampling moment**Individual* | 4 | 1 | 0.5021 | 9958 |
| Residuals | 4 |  |  |  |
| Total | 20 |  |  |  |
| **Geranyl acetate** | df | Pseudo-F | p | Unique perms |
| Treatment | 1 | 2.2769 | 0.2265 | 38 |
| Sampling moment | 1 | 2.2769 | 0.2266 | 38 |
| *Individual* | 5 | 0.8 | 0.581 | 9455 |
| Treatment*Sampling moment | 1 | 2.2769 | 0.2077 | 1348 |
| Treatment**Individual* | 4 | 1 | 0.4985 | 9836 |
| Sampling moment**Individual* | 4 | 1 | 0.5035 | 9808 |
| Residuals | 4 |  |  |  |
| Total | 20 |  |  |  |
| **α-Copaene** | df | Pseudo-F | p | Unique perms |
| Treatment | 1 | 1.5147 | 0.3925 | 38 |
| Sampling moment | 1 | 2.0752 | 0.2332 | 38 |
| *Individual* | 5 | 3.5804 | 0.1417 | 9968 |
| Treatment*Sampling moment | 1 | 2.0752 | 0.2213 | 1352 |
| Treatment**Individual* | 4 | 4.4755 | 0.0843 | 9957 |
| Sampling moment**Individual* | 4 | 1 | 0.5039 | 9841 |
| Residuals | 4 |  |  |  |
| Total | 20 |  |  |  |
| **Tetradecane** | df | Pseudo-F | p | Unique perms |
| Treatment | 1 | 0.75803 | 0.5427 | 644 |
| Sampling moment | 1 | 2.353 | 0.2261 | 645 |
| *Individual* | 5 | 5.0568 | 0.0873 | 9953 |
| Treatment*Sampling moment | 1 | 1.3964 | 0.3058 | 9312 |
| Treatment**Individual* | 4 | 7.9086 | 0.0323 | 9961 |
| Sampling moment**Individual* | 4 | 0.84943 | 0.5752 | 9967 |
| Residuals | 4 |  |  |  |
| Total | 20 |  |  |  |
| **β-Caryophyllene** | df | Pseudo-F | p | Unique perms |
| Treatment | 1 | 1 | 0.6249 | 3 |
| Sampling moment | 1 | 1 | 0.6406 | 3 |
| *Individual* | 5 | 1.2872 | 0.3119 | 9957 |
| Treatment*Sampling moment | 1 | 1 | 0.4666 | 12 |
| Treatment**Individual* | 4 | 1.606 | 0.2953 | 9967 |
| Sampling moment**Individual* | 4 | 1 | 0.5087 | 3017 |
| Residuals | 4 |  |  |  |
| Total | 20 |  |  |  |
| ***p*-Acetylacetophenone** | df | Pseudo-F | p | Unique perms |
| Treatment | 1 | 1 | 0.6343 | 3 |
| Sampling moment | 1 | 1 | 0.6379 | 3 |
| *Individual* | 5 | 74.018 | 0.0098 | 9957 |
| Treatment*Sampling moment | 1 | 1 | 0.4581 | 12 |
| Treatment**Individual* | 4 | 88.031 | **0.0006** | 9969 |
| Sampling moment**Individual* | 4 | 1 | 0.5121 | 3027 |
| Residuals | 4 |  |  |  |
| Total | 20 |  |  |  |
| **1-nor-Bourbonanone** | df | Pseudo-F | p | Unique perms |
| Treatment | 1 | 1.0001 | 0.4423 | 13 |
| Sampling moment | 1 | 1.0002 | 0.4491 | 13 |
| *Individual* | 5 | 2.0076 | 0.2213 | 9959 |
| Treatment*Sampling moment | 1 | 1.0002 | 0.3345 | 325 |
| Treatment**Individual* | 4 | 2.5095 | 0.1607 | 9957 |
| Sampling moment**Individual* | 4 | 1 | 0.5049 | 9570 |
| Residuals | 4 |  |  |  |
| Total | 20 |  |  |  |

**Table S7.** Pairwise comparisons of the effect of ‘Treatment*Sampling moment’ for the relative amount of 6-methyl-5-hepten-2-one in *Tocoyena formosa* flowers. Statistically significant results are marked in bold. SM: Sampling moment.

| ‘Treatment’ effect | | | | ‘Sampling moment’ effect | | | |
| --- | --- | --- | --- | --- | --- | --- | --- |
| Comparison | T value | p-value | Unique Perm | Comparison | T value | p-value | Unique Perm |
| Control SM1-SM2 | 2.617 | 0.1009 | 425 | SM1 Control-Before florivory | 0.97278 | 0.3996 | 255 |
| Florivory SM1-SM2 | 4.9992 | **0.0303** | 424 | SM2 Control-After florivory | 2.599 | 0.1161 | 425 |
